# A process for developing a sustainable and scalable approach to community engagement: community dialogue approach for addressing the drivers of antibiotic resistance in Bangladesh

**DOI:** 10.1186/s12889-020-09033-5

**Published:** 2020-06-17

**Authors:** Rebecca King, Joseph Hicks, Christian Rassi, Muhammad Shafique, Deepa Barua, Prashanta Bhowmik, Mahua Das, Helen Elsey, Kate Questa, Fariza Fieroze, Prudence Hamade, Sameena Huque, James Newell, Rumana Huque

**Affiliations:** 1 Nuffield Centre for International Health and Development, Leeds Institute of Health Sciences, University of Leeds, Worsley Building, Leeds, LS2 9NL UK; 2 Malaria Consortium, The Green House, 244-254 Cambridge Heath Road, London, E2 9DA UK; 3 ARK Foundation, Suite C-3 &C-4, House 06, Road 109, Gulshan2, Dhaka, 1212 Bangladesh; 4 Centre for Health and Population Sciences, Hull York Medical School, University of York, Heslington, York, YO10 5DD UK

**Keywords:** Community engagement, Antibiotic resistance, Antimicrobial resistance, Intervention development, Bangladesh

## Abstract

**Background:**

Community engagement approaches that have impacted on health outcomes are often time intensive, small-scale and require high levels of financial and human resources. They can be difficult to sustain and scale-up in low resource settings. Given the reach of health services into communities in low income countries, the health system provides a valuable and potentially sustainable entry point that would allow for scale-up of community engagement interventions. This study explores the process of developing an embedded approach to community engagement taking the global challenge of antibiotic resistance as an example.

**Methods:**

The intervention was developed using a sequential mixed methods study design. This consisted of: exploring the evidence base through an umbrella review, and identifying key international standards on the appropriate use of antibiotics; undertaking detailed formative research through a) a qualitative study to explore the most appropriate mechanisms through which to embed the intervention within the existing health system and community infrastructure, and to understand patterns of knowledge, attitudes and practice regarding antibiotics and antibiotic resistance; and b) a household survey – which drew on the qualitative findings - to quantify knowledge, and reported attitudes and practice regarding antibiotics and antibiotic resistance within the target population; and c) drawing on appropriate theories regarding change mechanisms and experience of implementing community engagement interventions to co-produce the intervention processes and materials with key stakeholders at policy, health system and community level.

**Results:**

A community engagement intervention was co-produced and was explicitly designed to link into existing health system and community structures and be appropriate for the cultural context, and therefore have the potential to be implemented at scale. We anticipate that taking this approach increases local ownership, as well as the likelihood that the intervention will be sustainable and scalable.

**Conclusions:**

This study demonstrates the value of ensuring that a range of stakeholders co-produce the intervention, and ensuring that the intervention is designed to be appropriate for the health system, community and cultural context.

## Contributions to the literature

- Research has shown that community engagement approaches to health can be effective. Those that have impacted on health outcomes are often time intensive, small-scale and require high levels of financial and human resources.
- We explore a process of developing an embedded approach to community engagement, which was co-produced by researchers, policy makers, programme managers, practitioners and communities.
- This study is a contribution to the literature on intervention development, which shows how linking an intervention into existing health system and community structures and ensuring it is appropriate for cultural context optimises its potential to be scalable and sustainable.

## Background

Community engagement approaches to addressing individual and population health can be effective [1–13]. A recent review identified influences on the effectiveness of community engagement approaches for communicable disease control. These included the application of key principles during the development of community engagement interventions, with shared leadership and tailoring being influential in determining the effectiveness of engagement on proximal and health outcomes [14]. However, a critique of community engagement is that approaches that have impacted on health outcomes are often time intensive, small-scale and require high levels of financial and human resources. They can be difficult to sustain and scale-up in low resource settings. Given the reach of health services into communities in low income countries, the health system provides a valuable and potentially sustainable entry point that would allow for scale-up of community engagement interventions. A challenge is to ascertain the balance between the inputs that are available within routine health service delivery contexts and the inputs that are required to ensure that community engagement is meaningful and effective.

Interest in implementation research [15], getting research into policy and practice [16], and embedded development and research [17] has tended to focus on health service delivery within facility settings. There has been less focus on how to embed interventions that aim to engage community stakeholders within the existing health system and community infrastructure. We understand an embedded approach to refer to two interrelated concepts: 1) that researchers, policy makers, programme managers, practitioners and communities co-produce the intervention and that, through this process of co-production and the subsequent experience of implementation, capacity is developed for researchers as well as within the health system and within communities; and 2) that the intervention is designed to be linked into existing health system and community structures, is designed to be appropriate for the cultural context within which it will be implemented, and therefore has the potential to be implemented at scale. Taking this approach increases local ownership, as well as the likelihood that the intervention will be sustainable.

This study explores the process of developing an embedded approach to community engagement taking the global challenge of antibiotic resistance as an example. We understand community engagement to mean a participatory process through which equitable partnerships are developed with community stakeholders, who are enabled to identify, develop and implement community-led sustainable solutions using existing or available resources to issues that are of concern to them and to the wider global community. Antibiotic resistance poses a significant threat to health and the World Health Organization warns that “without urgent action, we are heading for a post-antibiotic era, in which common infections and minor injuries can once again kill” [18]. In Bangladesh, resistance has been detected in most tested pathogens and many first-line drugs have been found to be ineffective [19]. Social mobilisation is one of a plethora of strategies recommended to address antibiotic resistance in Bangladesh [20].

The intervention described here brought together two existing initiatives, one focusing on provider behaviour, the other on user behaviour. First, the Revitalization of Community Health Care Initiative in Bangladesh, which aims to improve access, utilisation and equity of healthcare, was established by the Ministry of Health and Family Welfare in order to enable community clinics (CCs) in rural areas to deliver an essential service package to the approximately 6000 people in their catchment areas. Around 14,000 CCs have been built across the country and each has a community group (CG) and three community support groups (CSG) that form part of the management structure of the community clinics, deliver targeted health education, and provide links between the CCs and communities. A key part of this package involved training community health care providers (CHCPs), situated in CCs, to prescribe antibiotics correctly. An evaluation showed that 89% (95%CI 87–91) of consultations resulted in the correct prescription of antibiotics [21].

Second, we identified the Community Dialogue Approach (CDA) as having the potential to address antibiotic consumer behaviour through community engagement. The approach involves training community volunteers on a health issue and group facilitation techniques. Equipped with a set of visual tools, the volunteers host regular Community Dialogue sessions in their communities to explore the health issue, identify solutions and plan for taking action. This approach has been used in a range of contexts, including integrated community case management (iCCM) of malaria, pneumonia and diarrhoea in Uganda, Zambia and Mozambique, and prevention and control of neglected tropical diseases in Mozambique. A description of the approach has been published in an implementation guide [22]. Community Dialogue has been shown to be effective in filling health information gaps and helping communities make collective decisions for improved health practices [23], and a study evaluating the use of Community Dialogues to improve prevention and control of schistosomiasis indicates that the approach is feasible in resource-poor settings, well-received by the population and improves knowledge at population level [24].

**Fig. 1:**
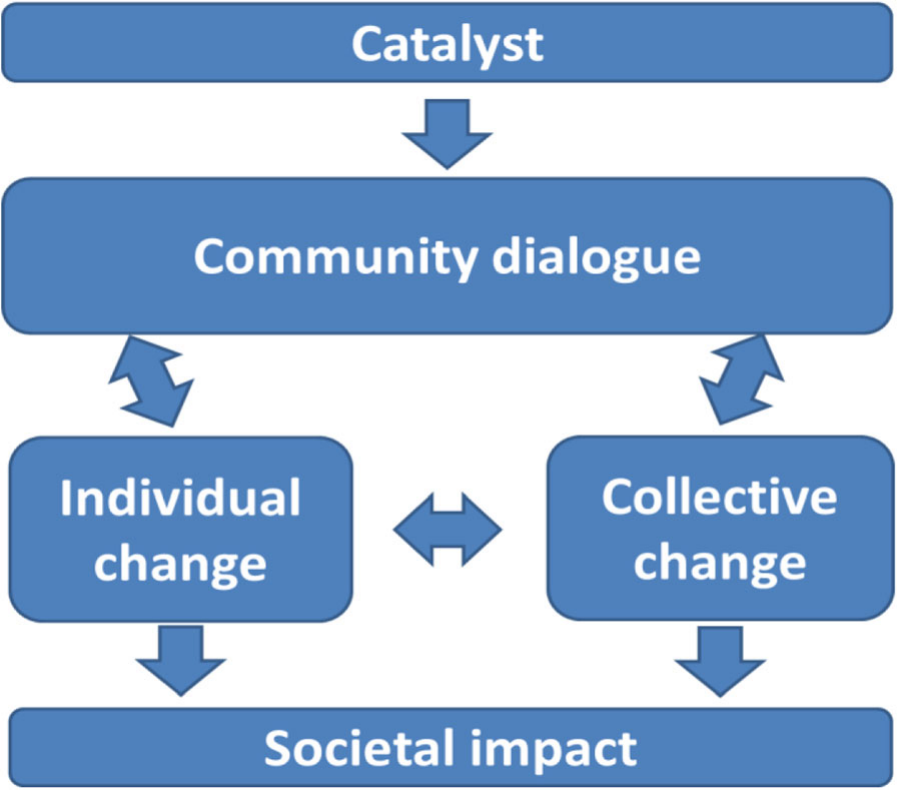
Adapted from: Figueroa, M.E., Kincaid, D.L., Rani, M., Lewis, G. (2002) Communication for Social Change Working Paper Series: No.1. New York: The Rockefeller Foundation

The CDA was adapted from the Integrated Model of Communication for Social Change [25]. The model assumes that a stimulus is required to trigger dialogue among community members about issues that are of concern for the community. Dialogue is understood as a dynamic, iterative process that results in collective decision making to resolve those issues. It is theorised that this process results in social change through increasing individual and collective self-efficacy, strengthening community ownership and shaping social norms. In the CDA, the stimulus is both external (provision of training and tools) and internal (selection of volunteers, volunteers mobilise participants to attend community dialogue sessions) to the community. While volunteers are given the flexibility to tailor each community dialogue session to the specific needs and requirements of the community, the sessions are designed to be highly participatory, giving all participants the opportunity to share experiences and voice concerns. Each Community Dialogue session concludes with participants committing to a course of action. Participants are also encouraged to spread information through word of mouth, set a positive example among family, friends and neighbours and to hold each other to account for applying decisions reached during Community Dialogue sessions [see Fig. 1].

The aim of this study was to adapt the CDA in order to address antibiotic resistance in Bangladesh. The hypothesis is that potential for impact, sustainability, scalability and value for money will be enhanced if the intervention is co-produced by key stakeholders and is designed to be appropriate for the health system, community and cultural context. Specific objectives were:

1. To conduct formative research to inform the content of and the processes for delivering Community Dialogues to address antibiotic resistance in Bangladesh;
2. To adapt the CDA to ensure that the content of and processes for delivering the intervention are appropriate for the setting, with particular emphasis on embedding the approach within the existing health system and community infrastructure.

## Methods

The study design was informed by critical elements drawn from frameworks on intervention development [17, 26, 27]. Specifically, the intervention was developed using a sequential mixed methods study design [28]. This consisted of:

1. exploring the evidence base through an umbrella review, and identifying key international standards on the appropriate use of antibiotics;
2. undertaking detailed formative research through a) a qualitative study to explore the most appropriate mechanisms through which to embed the intervention within the existing health system and community infrastructure, and to understand patterns of knowledge, attitudes and practice regarding antibiotics and antibiotic resistance; and b) a household survey – which drew on the qualitative findings - to quantify knowledge, and reported attitudes and practice regarding antibiotics and antibiotic resistance within the target population;
3. drawing on appropriate theories regarding change mechanisms and experience of implementing community engagement interventions to co-produce the intervention processes and materials with key stakeholders at policy, health system and community level.

Detailed findings from the umbrella review and from the investigation into knowledge, attitudes and practices regarding antibiotics and antibiotic resistance are reported elsewhere. This paper focuses on reporting on the process of intervention development as well as the most appropriate mechanisms through which to embed the CDA within the existing health system and community infrastructures of rural Bangladesh.

### Study setting

The study was conducted in one *upazila* (sub-district) of Comilla, a peri-urban district about 100 km south-east of the capital, Dhaka, with a population of 5.4 million. The district has around 410 functional CCs. Comilla was selected in consultation with the Ministry of Health and Family Welfare (MOHFW), based on the fact that members of the research team had previously worked with the MOHFW to enable the community health care providers in CCs in Comilla to deliver a basic package of essential care. The *upazila* in which we conducted the study was selected purposively, due to ease of access for researchers. The five CCs included in the study were selected purposively based on prior information regarding the functionality of the CG and the CSG. There is one CG and three CSGs per community clinic. Each group has 17 members. The members of the groups are clearly specified within policy, and they are supposed to represent a broad spectrum of the population. They encompass males and females, different ages, different socio-economic groups, and different professions. The members have been selected based on the information that is provided from the *upazilla* health complex regarding the categories of people to be included. The key responsibilities of these groups centre around managing the community clinic regarding issues such as opening and closing times, medicine supply, resolving problems related to electricity and other infrastructural issues. We selected two CCs with highly active groups, two with moderately active groups, and one with a relatively inactive group in order for us to better understand variation in the potential to embed the community engagement approach within the existing infrastructure of the CCs.

### Qualitative study methods

A formative qualitative study was conducted in order to: a. inform intervention design, by exploring potential key issues and implementation strategies, including the most appropriate mechanisms through which to embed the intervention within the existing health system and community infrastructure; and b. to understand the accessibility of health services, and patterns of knowledge, attitudes and practice regarding antibiotics and antibiotic resistance in order to inform the design of the household survey.

One interview was conducted with the Union Health and Family Planning Officer (UNFPO). Interviews were conducted with each of the five CHCPs who work within the five CCs. Ten focus group discussions (FGDs) each with 6–8 participants were held with community members. Participants were purposively sampled from the CG and CSG, two per community clinic catchment area, one male and one female. By selecting community members from these pre-existing groups, which are representative of a broad spectrum of the population, the variation of the sample was maximised in terms of gender, age, education, employment and socio-economic status and, therefore afforded us with a wide range of opinion within the limited resources and time available to us.

Interview and focus group discussion guides were developed collaboratively by the research team. Reviews of key international guidance on antibiotic stewardship, peer-reviewed literature, and existing knowledge of the health system and cultural context informed the design of the guides. Translations from English to Bengali were undertaken by the Bangladesh-based research team, who discussed the most appropriate terminology to convey critical concepts. Guides were pre-tested with two respondents per guide in a different CC catchment area from the five in which the study was conducted and minor adjustments made after the pre-testing.

All interviews and FGDs were held in private rooms within CCs. Interviews were conducted and focus group discussions facilitated by male and female researchers experienced in qualitative data collection (PB, DB, FF, SH). They were audio-recorded and detailed notes were taken. Interviews and focus group discussions were transcribed by two Bengali speaking research assistants and then translated into English by bilingual researchers. Transcriptions and translations were checked by FF, PB and SH. Data was managed using NVivo 11. Analysis of the data was undertaken using a framework approach [9] using the following steps: Familiarization - key themes were identified during a meticulous review of the transcripts; Thematic framework construction - themes deriving from the study objectives and other key issues that emerged from the data were identified and used to assemble a coding/thematic framework – this process was undertaken by two researchers with the coding frame being developed through a review of a sub-sample of transcripts; Indexing - the data were coded according to the thematic framework by target group and re-organized into sections under each theme - transcripts were coded by one researcher and the coded transcripts independently reviewed by another, with any disagreements discussed; Interpretation - each thematic area was compared between respondent groups, similarities and associations between themes were identified and findings were interpreted.

### Survey methods

The aim of the survey was to quantify knowledge and reported attitudes and practices regarding antibiotic use and antibiotic resistance, in order to inform the focus of the key issues to be addressed within the intervention. The survey tool was informed in part by the findings of the qualitative study.

We attach our questionnaire as supplementary material. We developed our questionnaire after reviewing findings from the qualitative study and conducting a rapid literature review of relevant studies [29–31]. Our questionnaire contained 85 questions for females: 42 in relation to themselves, 19 in relation to their children and 24 in relation to their husbands. We chose to focus on females, as time and resources prevented us from also surveying males and because our piloting demonstrated that females were able to respond to questions regarding themselves, their husbands, and their children on this topic. Prior to surveying we pilot tested the questionnaire twice to check it was understandable, feasible and acceptable for the respondents and interviewers, and adapted as necessary. The testing took place in Comilla district, but outside the study area, to ensure similarity in context. Women in five households participated in the pilot. Trained data collectors conducted the survey. We recruited four data collectors and one supervisor who were provided with two and half days training.

To obtain rapid responses to our survey we used a non-probability cluster sampling approach slightly modified from the WHO approach used in their Expanded Programme on Immunization (EPI) vaccination coverage surveys [32] which is known to generally achieve its aims in terms of providing reasonable estimates [33, 34], but it does have significant limitations and risks of bias [20]. However, as the primary aim of the survey was to provide rapid, cost-effective information to inform the development of the intervention the compromise was felt justified.

We aimed to survey a total of 245 women, as this would allow us to estimate outcome percentages and their 95% confidence intervals with an absolute margin of error of ±10% (suitable for our purposes), assuming an outcome of 50% (the least precisely estimable outcome percentage) and a design effect of 2.5 due to the clustered sampling.

We analysed the data using the R version 3.4.2 [35] and where necessary the “Survey” [36, 37] package. We first described the sample’s characteristics in terms of common socio-demographic variables. We then produced estimates of outcomes as either percentages (for categorical variables) or means (for continuous variables) with their associated 95% confidence intervals, adjusted for the clustered sampling design.

### Structured approach to developing intervention processes and tools

#### Interactions among the study team

The intervention was co-produced through a structured process of engagement with the wider research team, as well as with key stakeholders at policy, health system and community levels. A document outlining key issues, implementation strategy, and intervention tools was developed and updated throughout the intervention development phase.

When preliminary results from the formative research phase were available, a workshop was conducted, which brought together the wider study team to review preliminary findings, discuss implications for intervention development and refine key issues. It also served to identify knowledge gaps and means of eliciting required information. Throughout the intervention development phase, a small intervention development working group had weekly calls to review progress, discuss emerging findings from the formative research and recommendations from stakeholders, and make executive decisions. The wider study team was kept informed and provided feedback during monthly team calls.

#### Co-production of intervention with key stakeholders

First, a one-day a workshop was conducted with mid-level policy makers and practitioners with an interest in antibiotic resistance and community engagement in Bangladesh. In this workshop, participants refined tailored messages regarding antibiotic resistance and adapted them to the local context. Participants also provided feedback on aspects of the intervention design, particularly in relation to mechanisms to embed the intervention within the existing health system infrastructure. Second, a half-day workshop was conducted with representatives from the villages in the study area. This was used to validate key issues and obtain feedback on the proposed intervention design. Results, insights and recommendations from each workshop were summarised in a comprehensive workshop report. The Bangladesh-based study team continued to engage informally with key stakeholders throughout the intervention development period.

#### Development of intervention materials and pre-testing

Following the stakeholder workshops, a local artist developed visual materials illustrating the intervention’s tailored messages in an iterative process of drafting images and refining them based on feedback and suggestions from the study team. The images were used to develop a flipchart and a leaflet to support information sharing and stimulate discussion among Community Dialogue participants. The images were pre-tested in two focus group discussions with community members from a community in Dhaka (one with females, one with males). Discussions focused on establishing whether the drawings were understood as intended, whether they were culturally appropriate and whether community members liked their design. A range of non-visual tools to support sensitisation, training, community dialogue sessions, supervision, monitoring and evaluation were developed by the study team. All intervention materials were developed in English and subsequently translated into Bengali.

## Results

First, we present the key findings from our umbrella review, which are reported in detail elsewhere [14]. Second, we present four major themes, which emerged from the qualitative study and stakeholder feedback, and which informed the adaptation of the CDA, tailored for the health system, community and cultural context of rural Bangladesh. Third, we present key issues to be explored through the Community Dialogue.

### Key findings from umbrella review: context, mechanisms of impact, sustainability and scalability

The systematic review of reviews identified key contextual influences and mechanisms leading to a change in proximal or health outcomes, as well as factors leading to sustainability and scale-up. Contextual influences on the effectiveness of community engagement interventions identified in the review included the existence of wider partners, conducive socio-political context at community level and at state level, conducive place and social structures, nature of the health issue and its prevalence, strength of existing social cohesion and collective identity, and implementing organisation characteristics. Mechanisms identified as leading to change in proximal or health outcomes were increasing critical consciousness, a sense of ownership, autonomy and leadership by the community and building strong social cohesion, capital and trust often through increased networking. Other identified mechanisms included strengthened capacity for action particularly through skills and knowledge and engagement with wider partners and changing social norms and attitudes towards health behaviours and care seeking. Factors affecting the sustainability and scalability of the intervention were combining with the local health care system, multisectoral collaboration, generation of resource / economic incentives, aligning with cultural and social norms, community involvement in design and implementation of the programme, adequate finance/man power, incentives to retain volunteers, and continued contact between facilitators and community.

### Implementation strategy: culturally sensitive mechanisms through which to embed the CDA into existing health system and community infrastructure of rural Bangladesh

Analysis of the data from the qualitative study and stakeholder interactions generated four themes, each of which informed the design of the intervention. These are presented below.

#### Administrative and social organisation

It was very difficult to unpack the administrative and social structure, as slightly different responses and terminologies were used in different settings and by different participants (which may, of course, reflect differences). Rural communities are organised through both administrative and social units, which may or may not overlap. The administrative levels of *upazila* (district), union, and ward correlate with health facility levels of *upazila* health complex, union health complex and community clinic. The CC may serve several villages within the ward. Each CC is staffed by a CHCP, a health assistant and a family welfare assistant. The CC has a CG, and three CSGs, and each CSG is approximately linked to a village. However, the smallest unit is the *para* or *mohalla* or *bari* and there are usually several within each village. This is a social unit, which may – but does not always - consist of several related households.

Implications:

- given the size and expected sense of community cohesion, the village was selected as the appropriate unit of community dialogue implementation. However, explicit care was taken to ensure that each *para* was included;
- volunteer facilitators were selected by village and conducted Community Dialogues in their respective villages;
- the CSG was selected as the appropriate mechanism through which to embed the CDA into the formal health structure, given its link with CCs and overlap with villages. This was achieved by identifying CSG members to act as supervisors for volunteer facilitators.

#### Community meetings

In most settings, general meetings occur on an ad hoc basis. This may be during an election campaign or if there are particular issues facing the community such as crime, disputes, road construction, or natural disasters. These meetings tend to be organised by community leaders and they seem, according to most participants, to be attended mainly by males. Depending on the nature of the meeting, they could be held in public spaces such as the school grounds, or at the mosque. In some settings, “courtyard meetings” (*uthan bhoitak*) (are supposed to) take place. They are a forum for discussion of health issues. Some participants said both males and females attend these meetings, but not always at the same time. Generally, there did not seem to be a set schedule for running these meetings, although in one community clinic catchment area, the dates and topics were apparently set in advance. It was unclear whether they really took place across all community clinic catchment areas.

Implications:

- Community Dialogues were held separately for men and women;
- Volunteer facilitators were encouraged to liaise with local leaders to organise Community Dialogue sessions and mobilise participants.

#### Health education

There was general agreement that health education is (supposed to be) delivered by CHCPs, as well as the health assistant and the family welfare assistant. Some also stated that the CHCPs provide information to the CG and CSG members, who are responsible for delivering health education at the level of the para. Some NGOs also deliver health education. Most participants noted that health education is delivered verbally, usually in the form of a lecture and sometimes with the use of flip-charts and posters. Most participants also suggested that this mechanism is one that people in the community are comfortable with and should be utilised. Many participants emphasised that pictures are useful.

Implications:

- the CSG and its members were identified as a suitable mechanism for embedding the intervention into the existing health system and community infrastructure. This was achieved through providing supervision to volunteers as CSG members are often familiar with health issues and health education;
- it was recognised that communities were used to visual tools and printed materials to support health education; but there was also a critical need to emphasise the difference between uni-directional, specialist-led health education and Community Dialogues as a participatory, community-owned space for exploring health issues and taking action.

#### Facilitators

When asked what types of people might make good facilitators, responses were fairly consistent. Most participants noted that they would need some education and interest in the topic, and would also need to have some time available. Some mentioned characteristics, such as patience, humility, “good behaviour” and the capability to explain things well. Some suggested that more “powerful” or “influential” people (such as teachers, imams) would be best, but also noted that they might not have the time required to deliver the intervention. Some noted that it needs to be someone who is acceptable to all the community so that they are willing to participate in the meetings. Most participants were clear that it is important to have male and female facilitators. Some suggested minimum numbers (e.g. 3 of each within one community). Some suggested facilitators could be identified with the support of the CHCP, union parishad members, or community group and community support group members. Several participants suggested that the community support group members and the CHCP can support the supervision of facilitators. Some community support group members emphasised their willingness to be involved in this process. The issue of motivation / incentives was raised. Most participants felt that financial incentives would be important and several seemed to suggest that it would be difficult to motivate people without financial incentive. Some suggested that at a minimum any expenses need to be covered e.g. for transport and food. The UHFPO suggested 500 taka in a month if they worked 2–3 h per week. However, others noted that some people might volunteer because of the social prestige it may offer – one participant noted that “you will be known as a good person, everyone will honour you”. Some mentioned that some food and refreshments can be offered. Some noted that regular communication can keep volunteers motivated, one mentioned the importance of maintaining good relations with volunteers, and one noted that it is important to take their opinions into account when arranging meetings. When asked how the facilitators could potentially be linked into the health system, the following suggestions emerged: By involving the CHCP in supervision and monitoring and by involving the facilitators in the community group and community support group meetings.

Implications:

- the suggested characteristics were incorporated into the selection criteria provided to communities during sensitisation;
- male/female volunteers facilitated Community Dialogues with participants of the same sex;
- there were 2–3 pairs of volunteers per village, depending on the number of households; volunteers were unpaid, but some non-monetary incentives were provided.

### Developing key issues to explore through community dialogue: antibiotics and antibiotic resistance

Four key themes emerged from the component of the qualitative study that explored patterns of knowledge, attitudes and practice regarding antibiotics and antibiotic resistance. The household survey quantified this within the target population and helped to prioritise key issues to explore through the CDA. The major themes that emerged were on 1) antibiotics: knowledge, attitudes and practices; 2) antibiotic resistance; 3) accessing antibiotics; and 4) appropriate use of antibiotics. The key findings are presented elsewhere. Here, we present the priority issues that were identified through the formative research, through the stakeholder engagement and through our review of international standards on the appropriate use of antibiotics to explore through the CDA.

**Table:** 

1) Knowledge and awareness of antibiotics	• Different diseases have different causes. • Many diseases are either caused by bacteria or viruses. • Different types of medicines work for the diseases caused by bacteria and viruses. • If you take the wrong type of medicine, they will not cure the disease. • Antibiotics are medicines used to prevent and treat bacterial infections. • Antibiotics do not treat infectious disease caused by viruses. Common cold and sore throats are often caused by viruses and therefore antibiotics do not work against these diseases. • The antibiotics provided in regular health facilities pass through various quality controls and are very effective to treat the diseases caused by bacteria.
2) Knowledge and awareness of antibiotic resistance	• Many people use antibiotics often, even though they cannot prevent and treat all infections. • If used inappropriately, antibiotics may stop being useful for fighting infections in the future. This is called antibiotic resistance. • This is very dangerous as people may be sick more often or even die from infections that we have previously been able to control. • You can prevent infections and avoid taking antibiotics by regularly washing your hands, handling food in a clean manner, washing hands after contact with sick people and covering your mouth when you cough. • Do not throw left-over or expired antibiotics in the open environment as they may harm the good bacteria.
3) Accessing antibiotics	• Sometimes the symptoms of diseases caused by bacteria and viruses can be similar. • Only a qualified health care provider can diagnose what causes your disease and which medicines you need to cure it. • If you are severely ill, always go to a community clinic or another qualified provider for diagnosis and treatment. • Only use antibiotics when advised by a qualified provider to ensure you get correct treatment for your disease. • Do not take any antibiotics by yourself and only buy them from a pharmacy if advised by a qualified provider. • You can help ensure that antibiotics remain effective by only taking antibiotics when advised by a qualified provider. • Sometimes diseases may be mild – if you only feel mildly ill, you may not need any medication at all.
4) Appropriate use of antibiotics	• Always follow the advice of Community Healthcare Providers or other qualified providers about how antibiotics should be taken. • It is important to use antibiotics at the right time for the right duration. This will ensure they remain effective in the future. • Always complete a full course of antibiotics as advised by a qualified provider, even if you feel better. Sometimes people start feeling better before the infection is completely cured, but it’s important to get rid of the bacteria altogether. • By taking a full course of antibiotics as advised by a qualified provider, you help to ensure that lifesaving antibiotics will continue to stay effective for us, our families and everyone in the community. • Never save antibiotics for later or share them with others, as this poses risks for you and others.

## Discussion

The process of adapting the CDA to address the drivers of antibiotic resistance in Bangladesh was informed by well-established frameworks on intervention development [26, 27]. We have described a detailed process of stakeholder engagement, which contributes to discussions on the co-production of interventions. An exploration of the evidence base through an umbrella review, detailed formative research through a qualitative study and a household survey, and a series of formal and informal interactions with key stakeholders at policy, health system and community level resulted in the co-production of a community engagement intervention that has been explicitly designed to be linked into existing health system and community structures and be appropriate for the cultural context within which it will be implemented, and therefore has the potential to be implemented at scale. We anticipate that taking this approach increases local ownership, as well as the likelihood that the intervention will be sustainable and scalable. We use a modified version of the TIDieR checklist [38] to present the intervention that was subsequently implemented [Table 1].

**Table 1:** Community Dialogues Approach for addressing the drivers of antibiotic resistance in Bangladesh

ITEM	
**WHY** The CDA was adapted from the Integrated Model of Communication for Social Change. The model assumes that a stimulus is required to trigger dialogue among community members about issues that are of concern for the community. Dialogue is understood as a dynamic, iterative process that results in collective decision making to resolve those issues. This process results in social change through increasing individual and collective self-efficacy, strengthening community ownership and shaping social norms. In the CDA, the stimulus is both external (provision of training and tools) and internal (selection of volunteers, volunteers mobilise participants to attend community dialogue sessions) to the community. While volunteers are given the flexibility to tailor each community dialogue session to the specific needs and requirements of the community, the sessions are designed to be highly participatory, giving all participants the opportunity to share experiences and voice concerns. Each Community Dialogue session concludes with participants committing to a course of action. Participants are also encouraged to spread information through word of mouth, set a positive example among family, friends and neighbours and to hold each other to account for applying decisions reached during Community Dialogue sessions.	
**WHAT** **Materials** A range of non-visual tools to support sensitisation, training, community dialogue sessions, supervision, monitoring and evaluation were developed by the study team:	
**Intervention materials**	**Purpose**
**Sensitisation**	
Sensitisation sheet (villages)	Introduces the study and outlines selection criteria and proposed selection process for the role of community dialogue facilitator
Sensitisation sheet (supervisors)	Summarises the role of supervisors and expected commitment
Candidate contact details recording form	Used to record contact details of candidates for the role of community dialogue facilitators
**Training**	
Training-of-trainers manual	Describes content and format of a three-day training of trainers
Training manual	Describes content and format of a two-day training for all community dialogue facilitators and supervisors
**Community dialogues**	
Community dialogue flipchart	• Visually illustrates the intervention’s key messages, with messages printed on the back of each page • Intended to be used by community dialogue facilitators to stimulate discussion among the community
Community dialogue discussion guide	Lists questions community dialogue facilitators could explore with communities during each of the phases of the community dialogues
Antibiotic resistance leaflet	• Uses a selection of drawings and messages from the flipchart • Intended to be handed out to community dialogue participants to share with friends, neighbours and family
Community dialogue facilitators’ guide	The guide summarises the format and purpose of community dialogue and explains community dialogue facilitators’ roles and responsibilities.
**Monitoring and evaluation**	
Community dialogue report template	• Captures basic information about each community dialogue conducted • To be completed by the community dialogue facilitator
Decision log	Used by community dialogue facilitators to record any decisions made by the community during the community dialogues
**Supervision**	
Supervision checklist and report template	Takes supervisors through issues to be discussed with community dialogue facilitators during monthly supervision exchanges
Monthly community dialogue plan template	Helps supervisors and community dialogue facilitators to plan community dialogues for the coming month
**Procedures** A set of procedures around sensitisation, training, community dialogue sessions, supervision, monitoring and evaluation were implemented:	
**Procedures**	**Purpose**
**Sensitisation**	The research team invited key stakeholders (including, for example, CHCPs and Union Parishad Chairs) from each community to a sensitisation meeting. The study was introduced and they were requested to introduce the study within their communities and to facilitate the selection of community dialogue facilitators (based on criteria derived from the formative research) and supervisor from the CSGs and CGs.
**Training**	Members of the research team delivered a three-day training of trainers session in Dhaka; after which the trainers delivered two-day trainings for the community dialogue facilitators and supervisors.
**Community dialogues**	Community dialogue facilitators delivered community dialogues over a period of 6 months. Male facilitators delivered dialogues with male participants, and female facilitators with female participants.
**Monitoring and evaluation**	Community dialogue facilitators completed a brief report after each community dialogue, and a decision log of any decisions taken by the community.
**Supervision**	Supervisors held review meetings with community dialogues facilitators every month, using a check list and report template to guide the process. The supervision meetings also included planning for the next month’s activities.
An implementation guide can be found here: https://www.malariaconsortium.org/media-downloads/1185/A%20guide%20to%20implementing%20the%20community%20dialogue%20approach	
**WHO PROVIDED** Community dialogue facilitators were selected from within the community, using the following criteria (which were developed through the formative research): • Candidates should be adults • Candidates should be literate • Candidates should be passionate about improving health at village level • Candidates should be of good standing within their community • Candidates should be comfortable talking and leading discussions with community members Supervisors were selected from within the existing CSGs and CGs.	
**HOW** Community dialogue facilitators delivered community dialogues to groups over a period of 6 months. Male facilitators delivered dialogues with male participants, and female facilitators with female participants.	
**WHERE** Community dialogue facilitators were advised to identify an appropriate public space, such as a school building, in which to deliver the community dialogue.	
**WHEN AND HOW MUCH** Community dialogue facilitators were advised to identify a time of day that was suitable for participants to deliver the dialogue. They were advised to ensure that each area within their community was reached at least once per month.	

There is growing evidence regarding the extent to which community engagement approaches can impact on proximal and health outcomes. For example, Women’s Discussion Groups have been effective in addressing a range of health outcomes [2]; Community Health Clubs – as part of a package of community-based interventions – have impacted on proximal outcomes such as knowledge and reported practice [39]; and Positive Deviance, an approach that builds on the existing strengths of the community, has been very successful in improving the nutrition and health outcomes in over 40 countries [40]. However, as noted, there is a concern that such approaches are often time intensive, small-scale and require high levels of financial and human resources. They can be difficult to sustain in low resource settings, particularly when they are delivered as part of a research or implementation project. The adaptation of the CDA to address the drivers of antibiotic resistance in rural Bangladesh has explicitly set out to explore how a community engagement approach can be embedded within existing structures, in order to begin a process through which the balance between the inputs that are available within routine health service delivery contexts and the inputs that are required to ensure that community engagement is meaningful and effective is identified.

The study had several limitations. First, time constraints resulted in reliance on initial rather than final analyses of data. For example, due to its scale, the umbrella review took much longer than initially anticipated to complete. We incorporated initial findings into the intervention development and reflected on the implications of subsequent more robust findings but the study was not conducted entirely sequentially. Similarly, we prepared a rapid report of initial findings from the qualitative study, to inform both the design of the survey tool and the stakeholder engagement process. However, the subsequent detailed analysis of the data did not produce any remarkably distinct findings. The qualitative study focused on CHCPs and community members. However, it revealed the critical importance of community-based drug sellers within this setting and a more complete study would have incorporated their perspectives too. Moreover, selection of participants from the CGs and CSGs may have introduced biases and this decision also risks the reproduction of existing power structures within the intervention design (although these groups are structured to represent different elements within the community). Finally, our household survey was conducted with females only, as time and resources prevented us from also surveying males. Involving males may have produced different findings.

## Conclusion

Our mixed methods approach enabled us to draw on the findings from a systematic review, a qualitative study and a household survey to co-produce an intervention with stakeholders including policy makers, health service providers and members of communities. This study is a contribution to the literature on developing interventions and on embedded research. It specifically emphasises commitments to a. ensuring that a range of stakeholders co-produce the intervention, and b. ensuring that the intervention is designed to be appropriate for the health system, community and cultural context and, therefore, has the potential to be implemented at scale. Moreover, our study attempts to provide specific details on processes for developing interventions. We recommend a thorough methodical approach to intervention development. This is one way it could be done, which allows for an iterative approach and brings multiple stakeholders into the process.

## Supplementary information

**Additional file 1.** SG Members Female (1). Transcript of focus group discussion with female members of the community support group, region 1.

**Additional file 2.** CSG Members Female (2). Transcript of focus group discussion with female members of the community support group, region 2.

**Additional file 3.** CSG Members Female (3). Transcript of focus group discussion with female members of the community support group, region 3.

**Additional file 4.** CSG Members Female (4). Transcript of focus group discussion with female members of the community support group, region 4.

**Additional file 5.** CSG Members Female (5). Transcript of focus group discussion with female members of the community support group, region 5.

**Additional file 6.** CSG Members Male (1). Transcript of focus group discussion with male members of the community support group, region 1.

**Additional file 7.** CSG Members Male (2). Transcript of focus group discussion with male members of the community support group, region 2.

**Additional file 8.** CSG Members Male (3). Transcript of focus group discussion with male members of the community support group, region 3.

**Additional file 9.** CSG Members Male (4). Transcript of focus group discussion with male members of the community support group, region 4.

**Additional file 10.** CSG Members Male (5). Transcript of focus group discussion with male members of the community support group, region 5.

**Additional file 11.** Union Parishad Chairman (1). Transcript of interview with union parishad chairman, region 1.

**Additional file 12.** Union Parishad Chairman (2). Transcript of interview with union parishad chairman, region 2.

**Additional file 13.** CHCP CC1. Transcript of interview with community health care practitioner, region 1.

**Additional file 14.** CHCP CC4. Transcript of interview with community health care practitioner, region 4

**Additional file 15.** CHCP CC5. Transcript of interview with community health care practitioner, region 5.

**Additional file 16.** CHCP CC2. Transcript of interview with community health care practitioner, region 2

**Additional file 17.** CHCP 14.04.17. Interview guide for community health care practitioner

**Additional file 18.** CSG 14.04.17. Focus group discussion guide for community support group

**Additional file 19.** Union Parishad Chairman14.04.17. Interview guide for Union Parishad Chairman

## Data Availability

All data generated or analysed during this study are included in this published article [and its supplementary information files].

## Abbreviations

CC: Community clinics
CG: Community group
CSG: Community support groups
CHCP: Community health care providers
CDA: Community Dialogue Approach
iCCM: Integrated community case management
FGD: Focus group discussion

## References

[1] Cornish F, Priego-Hernandez J, Campbell C, Mburu G, McLean S (2014). The impact of community mobilisation on HIV prevention in middle and low income countries: a systematic review and critique. AIDS Behav.

[2] Prost A, Colbourn T, Seward N, Azad K, Coomarasamy A, Copas A (2014). Women's groups practising participatory learning and action to improve maternal and newborn health in low-resource settings: a systematic review and meta-analysis. Lancet.

[3] Skevington SMS, Sovetkina EC, Gillison FB (2013). A systematic review to quantitatively evaluate 'Stepping Stones': a participatory community-based HIV/AIDS prevention intervention. AIDS Behav.

[4] Farnsworth S, Böse K, Fajobi O, Souza PP, Peniston A, Davidson LL (2014). Community engagement to enhance child survival and early development in low- and middle-income countries: an evidence review. J Health Commun.

[5] Atkinson JA, Vallely A, Fitzgerald L, Whittaker M, Tanner M (2011). The architecture and effect of participation: a systematic review of community participation for communicable disease control and elimination. Implications for malaria elimination. Malar J.

[6] Kerrigan D, Kennedy CE, Morgan-Thomas R, Reza-Paul S, Mwangi P, Win KT (2015). A community empowerment approach to the HIV response among sex workers: effectiveness, challenges, and considerations for implementation and scale-up. Lancet..

[7] Kerrigan DL, Fonner VA, Stromdahl S, Kennedy CE (2013). Community empowerment among female sex workers is an effective HIV prevention intervention: a systematic review of the peer-reviewed evidence from low- and middle-income countries. AIDS Behav.

[8] Nachega JB, Adetokunboh O, Uthman OA, Knowlton AW, Altice FL, Schechter M (2016). Community-based interventions to improve and sustain antiretroviral therapy adherence, retention in HIV care and clinical outcomes in low- and middle-income countries for achieving the UNAIDS 90-90-90 targets. Curr HIV/AIDS Rep.

[9] Gale NK, Heath G, Cameron E, Rashid S, Redwood S (2013). Using the framework method for the analysis of qualitative data in multi-disciplinary health research. BMC Med Res Methodol.

[10] Salimi Y, S K, M H, L N, K A, J E, et al. Is Community-based Participatory Research (CBPR) Useful? A Systematic Review on Papers in a Decade. Int J Prev Med. 2012:386–93. PMC3389435 22783464

[11] Okwundu CI, Nagpal S, Musekiwa A, Sinclair D (2013). Home- or community-based programmes for treating malaria. Cochrane Database Syst Rev.

[12] Musa BM, Iliyasu Z, Yusuf SM, Uloko AE (2014). Systematic review and metanalysis on community based interventions in tuberculosis care in developing countries. Nigerian J Med.

[13] Medley A, Kennedy COR, K. Sweat M. Effectiveness of peer education interventions for HIV prevention in developing countries: a systematic review and meta-analysis. AIDS Educ Prev. 2009:181–206. 10.1521/aeap.2009.21.3.181 PMC3927325 19519235

[14] Questa KD, King M, Everitt R, Ferdous M, Barua T, Rassi D, Snell C, Putnis AC, Cartwright N, Huque C, Newell R, Elsey JH. Community engagement interventions for communicable disease control in low- and lower middle-income countries: evidence from a review of systematic reviews. 2020;19:51. 10.1186/s12939-020-01169-5. 10.1186/s12939-020-01169-5 PMC7137248 32252778

[15] Peters DHAT, Alonge O, Agyepong IA, Tran N. Implementation research: what it is and how to do it. BMJ. 2013;347. 10.1136/bmj.f6753 24259324

[16] KA WJ, Karam Shah S, Witter S, Wei X (2007). How to get research into practice: first get practice into research. WHO Bull.

[17] Walley JKM, Witter S, Huque R, Newell J, Wei X (2018). Embedded health service development and research: why and how to do it (a ten-stage guide). Health Res Pol Syst.

[18] WHO. Antibiotic Resistance: Key Facts 2018 [Available from: https://www.who.int/news-room/fact-sheets/detail/antibiotic-resistance.

[19] Ahmed I, Rabbi MB, Sultana S (2019). Antibiotic resistance in Bangladesh: a systematic review. Int J Infect Dis.

[20] Lemeshow SRD (1985). Surveys to measure programme coverage and impact: a review of the methodology used by the expanded programme on immunization. World Health Stat Q.

[21] Huque R, Ahmed F, King R, Walley J, Hicks JP, Elsey H (2016). Improving the quality of care of children in community clinics: an intervention and evaluation in Bangladesh. Public Health Action.

[22] Malaria Consortium. A guide to implementing the Community Dialogue Approach. London: 2018.

[23] Martin S, Leitao J, Muhangi D, Nuwa A, Magul D, Counihan H (2017). Community dialogues for child health: results from a qualitative process evaluation in three countries. J Health Popul Nutr.

[24] Rassi C, Martin S, Graham K, de Cola MA, Christiansen-Jucht C, Smith LE (2019). Knowledge, attitudes and practices with regard to schistosomiasis prevention and control: two cross-sectional household surveys before and after a community dialogue intervention in Nampula province, Mozambique. PLoS Negl Trop Dis.

[25] Figueroa MEKD, Rani M, Lewis G (2002). Communication for social change: an integrated model for measuring the process and its outcomes.

[26] Wight D, Wimbush E, Jepson R, Doi L (2016). Six steps in quality intervention development (6SQuID). J Epidemiol Community Health.

[27] Craig P, Dieppe P, Macintyre S, Michie S, Nazareth I, Petticrew M (2008). Developing and evaluating complex interventions: the new Medical Research Council guidance. BMJ..

[28] Cresswell JW, Plano Clark VL (2018). Designing and conducting mixed methods research.

[29] Padget M, Guillemot D, Delarocque-Astagneau E (2016). Measuring antibiotic consumption in low-income countries: a systematic review and integrative approach. Int J Antimicrobial Agents.

[30] Roess AA, Winch PJ, Akhter A, Afroz D, Ali NA, Shah R, Begum N, Seraji HR, El Arifeen S, Darmstadt GL, Baqui AH, Bangladesh Projahnmo Study (2015). Household animal and human medicine use and animal husbandry practices in rural Bangladesh: risk factors for emerging zoonotic disease and antibiotic resistance. Zoonoses Public Health.

[31] Roess AA, Winch PJ, Ali NA, Akhter A, Afroz D, El Arifeen S, Darmstadt GL, Baqui AH, Bangladesh, P. S. G (2013). Animal Husbandry Practices in Rural Bangladesh: Potential Risk Factors for Antimicrobial Drug Resistance and Emerging Diseases. Am J Trop Med Hygiene.

[32] WHO (1991). Training for mid-level managers: the EPI coverage survey.

[33] Henderson RHST (1982). Cluster sampling to assess immunization coverage: a review of experience with a simplified sampling method. Bull World Health Organ.

[34] Lemeshow STA, Tulloch JL, Dowd JE, Lwanga SK, Keja J (1985). A computer simulation of the EPI survey strategy. Int J Epidemiol.

[35] Team RC. A language and environment for statistical computing. R Foundation for Statistical Computing. 2013.

[36] Lumley T. Survey: analysis of complex survey samples R package version 3.322017. 2004.

[37] Lumley T (2004). Analysis of complex survey samples. J Stat Softw.

[38] Hoffmann TC, Glasziou PP, Boutron I, Milne R, Perera R, Moher D (2014). Better reporting of interventions: template for intervention description and replication (TIDieR) checklist and guide. BMJ..

[39] Boone PE, D. Fazzio, I, et al. Effects of community health interventions on under-5 mortality in rural Guinea-Bissau (EPICS): a cluster-randomised controlled trial. Lancet Glob Health 2016;4(5):e328-ee35. 10.1016/S2214-109X(16)30048-1 27102196

[40] Pascale T, Sternin J, Sternin M. The power of positive deviance: how unlikely innovators solve the World’s toughest problems: Harvard Business Press; 2010.

