# Supplementary material for: A process for developing a sustainable and scalable approach to community engagement: community dialogue approach for addressing the drivers of antibiotic resistance in Bangladesh
Source: BMC Public Health. 2020 Jun 17;20:950. doi: 10.1186/s12889-020-09033-5 (PMC7302129; doi:10.1186/s12889-020-09033-5)
Supplement: Supplementary file 1 — Additional file 1. SG Members Female (1). Transcript of focus group discussion with female members of the community support group, region 1. [file 12889_2020_9033_MOESM1_ESM.docx]

| **Study Name: Community Dialogue for preventing and controlling antibiotic resistance in Bangladesh: Case for Support** | **Focus group discussion:CC1 – Female community group members(CG) and community support group(CSG) members** |
| --- | --- |
|  | **Date of Interview:**  **09/04/2017** |

Information about the interviewee:

Gender: Female

Occupation:

- UP member
- Housewife
- Landless poor
- Teacher
- Health worker
- Widow
- Student

M = Moderator

P = Participants

M: Assalamualaikum (greetings). Thank you very much for agreeing to be part of this discussion. We are from ARK Foundation, which is a research organization, and we are working on behalf of the Government of Bangladesh, particularly from Community Clinics Unit. [Within the Ministry of Health and Family Welfare, as well as two organizations from the UK - the University of Leeds and Malaria Consortium]. We are providing some technical support to the Government of Bangladesh to help your community. For example, we will provide some training and materials that will help the government to provide some health education about the use of antibiotics. Participation in the study is an opportunity for you and other people to tell us your experiences, opinions and ideas. There is no right or wrong answers. All your experiences and ideas are important. During the discussion you can leave at any time and you do not have to explain the reason. We will not identify anyone by name in anything that we write, so please feel free to speak openly. As it is a group discussion so want you to talk one by one in a particular issue otherwise it will be a mess. Furthermore, for our convenience we want to record the discussion in audio recorder. If all of you are agreed then we will collect your sign in the consent form and will proceed for the discussion.

Let’s start-

**M: I would like to understand this area. Please can you explain the administrative breakdown of the area?**

P_4_: As far as we know, it is only after any district that is the upazila, then the union, then the wards and the villages. There are some localities in a village. In some places, village is the last administrative area but in other places small localities exists.

**M: Can you tell me, how many small localities make a village?**

P_3_&P_4_: We have 17/18 small localities in our area.

P_5_: I think we have 18villages.

**M: If you have 18 villages then what about wards?**

P_3_: There are 9 wards in our area. Each ward consists of 2/3 villages. We are under ward no 7 and this ward consists of 3 villages. We belong to Doiyapara village.

P_5_: And each village consists of 8-10 small localities.

**M: Well, don’t you have any meeting here (I mean in your village)?**

P_4_+P_5_+P_6_: Yes, there are meetings here in every month.

**M: I am not talking about community clinic meeting. Are there no meetings arrange here for any other reasons?**

P_6_: We have never seen.

P_5_: Generally large meetings are arranged in a good place such as in playgrounds or in school grounds.

**M: Well when and why these meetings are usually organized?**

P_5_&P_4_: Generally during the national election or for the arbitration meetings, those arrangements were in big. During that time all the people of village were gathered for discussion on an important issue.

**M: How are they informed about this big meetings and who does this responsibility?**

P_5_: Everyone is informed by miking. Those who usually lead are responsible for this arrangement, for example, chairman, members of the union parishad.

P_4_: Especially the members of the union council have information about all the inconvenience of the village people. If there is any problem or accident, everyone goes before the member then they went to the chairman.

**M: Who report the inconvenience to the member?**

P_3_&P_5_: The person who have any problem he makes his own complain to the member of the union parishad or sometimes someone else help him to report. After discussing the problem with member, he (the member of union parishad) advised what would be good to do.

**M: What I am trying to convey, how do you gather all people in a specific place? (Once you mentioned about miking; I want to know about other ways)**

P_2_: Suppose, we requested the chairman that we want to discuss a problem with him. Then he fixed a specific day and time to discuss the problem with other important persons. Then we tried to find out a solution by discussing it all together.

**M: Who are the important people in this area?**

P_2_: Some people, such as chairman, members of union parishad who are politicians, who do something for the country, they usually take the responsibilities of informing entire village people through miking about those different meetings. That is when, where, what will be the meeting-usually informed about it.

**M: What is the duration of these meetings?**

P_6_: Approximately 1 / 2 hours, depending on the type of work and issues.

**M: Well, do you have any meeting about health related matters in your village?**

P_2_: No such thing happens to us here. Health related meetings are usually organized in the upazila.

P_3_: Once a meeting was held in the upazila at the Barkhola school ground. The chairman called us then we went there and participated in the meeting.

P_4_: If the meeting is announced beforehand, it is easy for everyone to get together. As an example, you will come from Dhaka, it told us earlier. Where will this meeting be, what will be discussed in the meeting, how long this meetings will be conducted, we have been told all the time so we all can be in one place easily.

**M: We cannot come from Dhaka all time. When we do not come, do the Community health care provider, Upazila family planning officer, organize a health related meeting at Upazila health Complex?**

P_5_: Here we do not have any health related meetings with the common people.

P_6_: Here, at the meeting, people come from all the above labels. When Kausar vai asked us to come to the meeting, we try to present in the meeting.

**M: Who are these “we”?**

P_6_: “We” are Community group members and Community support group members, who are in the committee.

**M: Well, if your common people have to give some health related knowledge, how do you give them?**

P_5_: We usually go to one person’s house and inform people about various health related information. Our responsibilities are fairly divided as I have been told to visit some house-holds. Similarly, others were also told. But no one is invited to sit at someone’s house.

P_1_: Whenever there is a local election, politicians organize meetings at any school ground or in a big place. They inform all the people of the village about being present in certain places, at certain times through miking. The people of the village go to the meeting and participate in discussion with the Chairman or members.

**M: The meetings which are held here, whether they are arranged for men and women together or separately?**

P_6_: Meetings are for everyone but most of the time men are present in those meetings because women are usually busy with their household chores. So they don’t want to participate in those meetings.

**M: Women don’t want to come too much in the meeting or they are not asked to come-**

P_6_: Actually women can’t come to the meeting due to their household works but they are not asked to stay in the meeting in the same way as the men are asked. Because many people in the village do not like the participation of women in the meeting. Besides this many women are criticized if they express their opinion in the meeting.

**M: As long as you talk about local elections. Now if you have a meeting or a court yard meeting** **to talk about health issues, how do you think it will be?**

P_4_: It would be good if we take such initiative.

P_5_: Sometimes we arrange court yard meetings. When we went to a house for any health related discussion then other people come from nearby homes.

**M: Do you need go to everyone’s house and arrange these court yard meetings?**

P_4_: No, this area has been divided into 8 centers to hold a court yard meeting.

P_6_: These meetings are organized in certain homes on a particular day of the month. In our village we have set the centers at different houses.

**M: Based on what, you have divided the whole village into 8 units for your convenience?**

P_6_: Our village is very big but there is only one community clinic. Everybody comes to this clinic for their problems. So, it is not possible to provide all types of services here. Hence, we have fixed someone’s house on a fixed date in a month for special services such as immunization of children, discussing different methods of family planning or distributing content of different family plans. In this way, 8 centers have been made in the village and the courtyard meetings are arranged in those centers according to pre-fixed time and place alternatively.

**M: Do you only conduct the meeting or accompanied by someone else?**

P_3_: We also have health workers with us. He/she talked about health related issues with women in the village.

**M: Are these courtyard meetings organized separately for men and women or do they get together?**

P_2_+P_3_+P_4_+P_5_: No, not for men. Only women come to the meetings.

P_6_: No. men also come in these courtyard meetings. If the women are busy with house-hold work then the men bring their child to vaccination. Sometimes a child’s grandmother or aunt comes too. All types of people come but women come in more because women related health issues are discussed more.

**M: Well, now I want to ask you a link to your work with the community clinic. That is, what is your main job with the community clinic?**

P_6_: If any person in the village gets sick, they come to us first for advice. Then we advised them to go to this community clinic. Earlier, everyone did not know about the clinic. People have come to know about it for us. Now fairly good patients come to the clinic.

P_5_: There are free treatments here. Many people in the village cannot buy the medicine. Although there are upazila health complexes but it costs 50 rupees to go there. My husband is an Upazila Health Assistant When people come to my house, he also tells them to go to the community clinic. The patients can collect medicines very easily from there.

**M: So, what you mean, the people of the village did not know about the community clinic before, but because of yours’ preaching now they know about it. The number of patients is also growing faster than before. It is your job to tell people about the services of this community clinic, or to inform people about community clinics**.

P_5_+P_6_: Yes, Yes.

P_6_: I give you an idea, when there is a disease of the people of the village, we talk to them about the community clinic. We say, there is a community clinic and there are medicines or treatment available there. Now the people of the village come to the clinic before any illness and then need to go somewhere else if needed.

**M: Well, now I want to know that here you are members of two groups, namely community group and community support group; is there any difference between your works?**

P_6_: I can’t talk about their work separately.

P_5_: The job of Community Group is to find out whether the medicines are available at the Community Clinic, then entering the names of those who are being given medication and writing the instructions for taking medication on those.

P_5_: Community Support Group members do not have much work to do. Their main work is to inspire the people of the village to go to the Community Clinic - that is, to be treated for medical treatment, to go there for medicines. In general, their responsibility is to inform the people of the village about the clinic.

**M: Well, do the members of the community group get some rewards for their work?**

P_5_: No, they are like volunteers; usually work for the benefit of the people. They do not get anything in return.

**M: They are working like a volunteer, how they are getting the interest to work from themselves for others?** **What I want to say, members of this community support group perform their responsibilities so well, but they do not get any money for this. Yet how do they perform their duties?**

P_7_: Actually, our mission is that, all the poor people in our area can get the right treatment at the right time. There are many people in the village who are not treated due to lack of money. We ask them to come to this community clinic because free medical and medicines are provided here. Many people do not know this fact, we have told them to come to the community clinic. In my opinion, by doing this we benefit them. People know us very well and also respect us. To do something for the poor people, we feel proud and honored.

**M: This is really a big deal. Now we want to know, what is the rule of hiring you in this work system?**

P_7_: I think, regarding recruitment, Kausar vai and Helal vai can give you the best information.

P_5_: No, the recruitment is largely settled by discussions in this community clinic. The person is elected as members in the village by checking their status and their ability of taking responsibilities.

**M: Who selected those members?**

P_6_: Kausar vai

**M: Kausar vai! He alone took all the decisions!**

P_6_: Actually Kausar vai usually organizes any meetings-

P_5_: In the meeting, there are respected people of the area and also people from the upazila hospitals. Actually we all know from Kausar vai. He told us everything he has done in the meeting.

P_6_: Kausar vai used to present in the meeting. Besides him, a health assistant, a field worker, and the honorable people of the village present in the meeting and decide who will be elected as the members of community group and community support group.

By sitting in the meeting, they decide who will be responsible if they give them any responsibility. There are some women in the village who do not go out of their house. If you chose them for this work that should not be wise. Keeping these things in mind, they choose members. No recruitment notice is given to them. Everything is fixed through discussion. It is mandatory to have a member of Upazila, freedom fighters, school teachers in this group.

P_5_: Now I'm sitting here leaving my child in the house; here I am not coming for my own sake, I am coming for the betterment of the health of common people.

**M: Well, do you have any time limit for your work here? I mean, whether you have to work for 1 or 2 year in this community?**

P_5_: There are no such rules. However, after the local elections, members of the community group were changed. Because when the new chairman and member will be elected, they will later join to the committee.

P_6_: Or if any teacher of the school is transferred then the new teacher is replaced in the committee. Otherwise, the elected members remain same.

**M: Are there any meetings for your work-plan? Are all members present at that meeting?**

P_7_: Yes, we always have a monthly meeting. Not everyone can attend all the time, but most of the members try to participate in the meeting.

**M: Well, even if a member is not present in three or four consecutive meetings, then how do you handle that matter?**

P_4_: In such a way, his membership will be cancelled.

**M: In this way the membership is canceled! Is anyone else employed instead of that member?**

P_5_: Yes, another is recruited.

**M: Thank you. Now I come to the real issue. You are shaking your head; after hearing that we have not come to the original issue? We will come back to the real issue now. We are trying to do something from the Health Ministry. That's why we need some helpers. You're already giving time at the Community Health Center, is not it? So, considering the interest of the area, if we want to take some volunteers, what do you think will be good and based on what?**

P6: What do you mean by the volunteer work?

**M: Volunteers' main work is to give health related knowledge to others. Of course, this CHCP is doing her job very well. But the issue is only disseminating knowledge about antibiotics. Do i make you understand the matter? The fact is that their (volunteer) work is not much. Their main task will be to provide an inclusive view about antibiotics among the common people. That is why we want to know your opinion, who will be better if we take a person as a volunteer.**

P7: Will you take the volunteer from committee person or take someone from the outside?

**M: Actually we can take the Volunteer from you and take it from outside. If you get from your community, it is easy for us to know how well it can be done. But in this case you may have the possibility of increasing work pressure. On the other hand, if we recruit them outside of your committee, you have to supervise their activities like a teacher. That is why we want to know from you which person will be best as a volunteer and why?**

P4: I think everybody who is here has completed their education up to secondary level. I think this work requires educated women; especially those who have studied but are unemployed. Is it not good to take such educated girls? Girls will work unselfishly. A woman can arranged a court-yard meeting, and after that, she will do this work for some remuneration.

P5: Well, the volunteer's job is to be done for a period of time or for a specific period of time?

P6: I think it would be better if some educated girls are taken as volunteers.

P5: At first, the whole have to narrate to them properly and then they should be trained. Without training, nothing will happen.

**M: Why do you repeatedly talk about girls? What happens if you take the boys?**

P6: A girl can easily open up her mind to another girl; what they are ashamed to say in front of a man. And a girl worker can talk to a woman or a man in a house. And you have to say everything you want to talk about. All of them have to be told. Elder-younger people will have to know the message.

P5: There are more people like you who have done such work in different district upazila. They kept numbers in the house from different areas. These things they do with money if you give some money, this will be done by all the boys and girls in the area. Again everyone will not do this work (changed her opinion here). Those who are illiterate, they will do the job.

**M: (Then the Moderator asked everyone by looking at the Topic -guide) – If you want to add anything else to this discussion then you can do it.**

P6: I think we have nothing to say about this.

**M: That is, if I do not get it wrong, you think that the voluntary we want to take will be educated. Besides, what do you think they need to have?**

P6: Educated but unemployed

P5: Those who are educated but unemployed will be better off. Educated married girls do not get time because they have children, they have a family. Again, the girls who are in education also do not get time because they have school-college, tuition.

P3: In the simple words, the name of the medicine you have told about we should aware people when they should use it or not. We should not carry those medicine from door to door. Those medicines should be distributed from this community clinic, isn’t it Apa?

**M: It is the key to giving this knowledge to your common people, because the CHCP brother has many responsibilities. And also all of you have many responsibilities.**

P7: Do you need any educated person for this purpose?

**M: Of course, now a day an educated person is needed for everything.**

P5: Well, now educated people are need for all the takes because if you select an illiterate woman, she can’t tell anything about antibiotics. Everyone does not have the ability to make other people to understand something.

**M: But if I ask you as an area, I mean if I ask people from Dhaka, to talk with the people of Doiyapara. So how do you think it will be?**

P6: No, no, they cannot. Here People will not listen to them.

**M: This message should reach the ordinary people. There are many people who are not educated at all, you know it very well.**

P7: Many people cannot write their own name properly.

**M: we have to convey the message to those illiterate people. In that case, the volunteer we will take, what kind of volunteer you think will be better?**

P6: Another thing, suppose Apa - if you give the job to young boys or girls they will not do that properly. In my opinion, the adult will have to take this job. To make the illiterate people understand, sometimes we have to treat them as our parents and also behave with them very friendly so that they must enter the message inside their heads. Then the work will be successful. But this will not work with young boys or girls.

P5: Those who go to college, they will do this job for very little time

P4: Hey, when we went to the college, we used to go for any kind of work related with mankind.

P3: But now our children do not want to go to these jobs.

P4: (She asked the interviewer) I think, you will be younger than us? When we used to study in college, arsenic was a complex disease. Now everyone knows about this disease. But then we did not get so much money. We used to be given only travel cost. We used to do this enthusiastically. There was a 5-day training for the area. After giving us training we asked to go to different areas. I did not know how to do this work before but it has become easier now.

P2: Listen, I remember, once a cholera vaccine was invented.

P5: Yes, Yes

P2: They did the job of vaccination finding the students. I have to remember. I read it in class nine. They selected me as community health worker. Then they gave me training on how to give the vaccine to humans. After training, how much of the spindle in the area that this vaccine will cause infertility to human being. I saw no one wanted to take this vaccine. Later, we had to explain the benefits of this vaccine at door to door. While doing this work, I thought that if I have had a vaccine, then maybe the wrong conception of the people will be gone. So once I went to a home and took a vaccine breaking the ampule. Everyone in the area used to eat those vaccines after this incident. In order to do this, I got the first prize. They (authority) tried to keep me in the office but my mother did not give permission.

P5: Before people did not want to understand easily.

P2: At that time, I was not married so people considered my initiative seriously. After this, everyone agreed to take the vaccine

**M: Let me now say that if we choose the Volunteer in such a way that a man volunteer for the male, a woman for the female or men / women any of them who will talk to everyone.**

P6: Actually, if a man and a woman join a team, then it will be better to work.

**M: Then, what else?**

P6: We are but wife of this area. We cannot even discuss all the issues to all the men. In this case, a man can speak freely with another man. So to talk to people, both men and women will need it. For this purpose, they should be trained properly and be included in a team. To make their (volunteers) work easy, their visiting places should be divided into a small community or in a village.

**M: What if, their working places are given based on village?**

P6: I think it would be better if their working places are given based on ward. There are many people in a union.

P7: In my opinion it is best if the volunteer is taken from us. It is better to share the members among us as their respective villages or households.

**M: Basically, I want to know from you, what would be the best thing to do?**

P5: May be, they (the research team) want to work with the volunteers.

**M: No, no, I do not mind what you say. I really want to know how it works effectively. Now do you understand that?**

P4: I think it is best to work through us.

**M: Well, in your opinion, it would be better to work through this Community Support Group. In that case, the house of all the members of one of your group is in this village of Doiyapara?**

P5: We are 3 of this group from Doiyapara village, but our house is different.

P6: If you take us for the duty to do it well, it will be done very well. If I took the responsibility given by you, but I did not do it properly, then the work will not be done properly.

**M: I just like your words. However, I would like to make you understand that this work should not be confined to Doiyapara village only. We want to do this work in other places. But believe me, the members of community groups of all other villages are not like you. Look, there is no difference between their and your opportunities but they are not working properly. You told me a while ago that you are working unselfishly but why are the members of other places not working? Why did they leave the job midway? It is very important for us to inform general people about antibiotics. If there is a community group or not, we do not want to stop the work. So, we want to include community group members to monitor this work. And if we choose the Volunteer, then should they give some money or tell them to work unselfishly like you?**

P6: They will not work unselfishly. Do you know why they don’t do so; the children of this area have some demand. Now everyone is old enough to understand about the betterment of the country like us. We may consider that this village is my in-laws' neighborhood. We will get advantages if the area is developed. Everyone here in the village has made a place, there is a clinic, so it is a place of honor. In this interest, some people in our village are benefitting. Here we also have an interest.

**M: Suppose, what they can be given instead of money? What is your opinion on this?**

P6: Apa, what I want to say briefly - do not leave this decision to us, and you decide who will you select as a volunteer. We people will have different opinion and that will mess up everything. But if you decide, everyone will accept it.

**M: Well, I realized it would be better to make the decision from lower level, and then share with the top level? How does a tree grow up - from bottom to top, not so; And see, the tree's root (bottom) is very tough.**

P6: Not all systems are but the same way.

**M: So, we want to know from you, how do we do it?**

P4: I also want to know from you about how you would like to work. After listening to you, I will give my opinion.

P6: Actually, what will be the job?

**M: The task is to give general public knowledge about antibiotics.**

P6: What kind of knowledge?

**M: Knowledge of good and bad about antibiotics - that is, what happens when we take antibiotics?**

P6: Cannot we know about this?

**M: Of course, you can.**

P6: Actually, we think that antibiotics will recover our disease perfectly.

**M: Antibiotics are good medicines but in some cases-**

P6: But again think that it will cause damage to our body by taking more. Doctors say this medicine can be taken to a specific time, such as 3 days, 5 days or 7 days. If there is a gap falls in the middle of the treatment, then the course of this medicine have to starts from the beginning.

**M: You're not saying anything about community clinic**

P6: Antibiotics cannot be taken with gaps

P5: It has to be taken on time

P6: Otherwise you have to start the course from beginning

P3: Apu, is this true? The doctor prescribed me antibiotics, 4 times in a day that is 6 hourly one capsule... If I use to take a medicine for more than one hour, what will be the effectiveness of antibiotic?

**M: If someone could give a cup of tea to the member Apa, then it would have been very good. She will just fall asleep.**

P4: no, no

**M: Member Apa, don’t you want to talk anymore?**

P4: I'm just telling you what I want to say. You guy (the members) think about the fact that Madam said. I have a little work. I have to go to the market.

**M: I will not take my time anymore - and only 10 minutes I'll talk to you. I know you came here with a lot of trouble.**

What I want to mean is that, members of the Community Support Group cannot always go door to door to talk about this antibiotic. We want to give this knowledge through someone else.

P5: That's what we understand.

**M: Well, then how and who will select these volunteers for this job?**

P5: Apa, how many people will be selected based on the village?

**M: What do you think regarding this issue?**

P6: Our village is a big one

**M: Yes, it is. How many would be better? Whether based on the size of village or based on the community clinic?**

P5: I think it would be better to take the volunteers on clinic area based. That means the area should be divided on the basis of the wards surrounding a community clinic. Otherwise you need a large workforce to accomplish the target.

P6: Suppose, this sister in law is a teacher. When she talks about something important people usually listen to her. Everybody in this area will listen to the words of Member Apa. This sister is a daughter in law of a house, family members will listen to her. In fact, you have to choose people to whom other people will pay attention.

P7: Yes, yes, the main fact is that.

P6: It was found that you gave this responsibility on a young child but nobody heard of it. You have to select someone educated so that he/she could make people understand the issue very well.

**M: Well, you said who would be better as a volunteer. Now, how will it be, if we assign you with the responsibility of monitoring them after selecting these volunteers? Can you do that?**

**Actually, this monitoring is mandatory in all systems. Look, my madam has come from Dhaka to see if I can take your interview properly.**

P6: They are coming to see this meeting! That's why they are taking note of everything.

**M: Exactly, this is what I want to say….**

P5: What I want to say, after end of the meeting, you sit with the Kauser vai and the member of the union parishad and discuss whom you want to take as a volunteer. Everyone will agree with your decision.

**M: Member Apa, you said that there is a lot of work to do after the meeting. Now everyone wants to give this responsibility to you. Your opinion-**

P6: There are also male members. This apa is a member of ward number 7. But this community clinic is in ward no 8. Ward No. 8 has a male member. It is good to discuss with them.

**M: Now my last question to you is - do you talk about health issues only through words or do you have a flip chart or book?**

P2: We talk to people at home and face to face. (Everybody agree with her statement)

**M: How many centers are there?**

P4: 8 centers

**M: Based on what these 8 centers have been decided?**

P4: There is division in it. For example, at one home, there is one center. It is usually visited once in a month in any home. There are 6 villages - Dulia, Doiyapara, Sarker Pool, Hadia, Bulirbag - 8 centers in these 6 villages.

**M: Is these six villages under a ward?**

P5: These six villages are divided under three wards. Previously, all these six villages were under ward no 3. But now the local Government divided the old big ward and made three separate wards.

**M: Well, now tell me how many villages are there in a ward?**

P5: Among the three wards in this area, one ward consists of a village, another ward consists of two villages, and the third ward is made up of three villages.

**M: Now if you select the voluntary by calculating the population, what would be better if you take it as a ward or if you take it as a village?**

P6: In my opinion, ward-based selection will be best because then you can take volunteers from the village within that ward.

**M: Well, today I come to know with many things through the discussion with all of you. Thank you all for being friendly and for managing your valuable time. Now, do you have any question?**

**Okay. No question! Then we will stop our discussion here.**
