# Supplementary material for: A process for developing a sustainable and scalable approach to community engagement: community dialogue approach for addressing the drivers of antibiotic resistance in Bangladesh
Source: BMC Public Health. 2020 Jun 17;20:950. doi: 10.1186/s12889-020-09033-5 (PMC7302129; doi:10.1186/s12889-020-09033-5)
Supplement: Supplementary file 2 — Additional file 2. CSG Members Female (2). Transcript of focus group discussion with female members of the community support group, region 2. [file 12889_2020_9033_MOESM2_ESM.docx]

| **Study Name:** **Community Dialogue for preventing and controlling antibiotic resistance in Bangladesh: Case for Support** | **Interview ID: CC2 Female FGD** |
| --- | --- |
|  | **Date of Interview:**  **11/04/2017** |

M = Moderator

P = Participant

P1: Health worker (Community support Group)

P2: Member (Community Group)

P3: Student (Community support Group)

P4: Teacher

P5: UP Member

P6: Family Planning worker (Community Group)

P7: Member (Community Group)

M: Where do people in this community go?

P1: People of this community usually come to the community clinic first for getting their primary health care services. They also visit to Gauripur Hospital, Daudkandi hospital, Private clinics, Surjer Hashi clinic which is in Shohidnagar ( Sundolpur Union)

P6: People generally visit to paramedics in Shahidnagar.

P6: Sometimes they visit to paramedics in Shundolnagar

P6: People mostly visit homeopathy clinic in Dashpara (DMF Chamber) for treatment

P1: People visit to Shurjer Hashi clinic in Shundolpur union

P5: We also visit some private hospitals at Gauripur

P4: People usually visit Upazila health complex at Gauripur union

P7: They go to Gauripur and Daudkandi where two public hospitals are situated

P1: Sometimes they visit Dish, Mukti, Shapla, Ibne sina hospital at Guaripur

P2: We visit several private clinics and hospitals at Shahidnagar and Shundolpur

M: Why people visited these places?

P1: Most of the people of the community usually come to the community clinic of Shaatpara for their primary health services because it is nearer to them and services are very good.

P3: We rarely go to the other health care providers because of long distances from our locality.

P5: We only go to the other hospitals and clinics if the condition of the patients are serious and not treatable in the community clinic.

M: Are there any differences in types of people who visit these places?

P4: People of every age even the pregnant women and children come to community clinic for treatment.

P6: In case of emergencies people go to hospitals and other clinics in Gauripur

P5: Financially solvent/ rich people also go to the private hospital and clinics because they do not have faith on these free services.

M: Do people visit traditional healer?

P4: No one believe in traditional healer in this community

P6: No one believe in traditional healer in this community

M: Do you have any pharmacy close to community clinic?

P4: Yes, we visit a pharmacy which is in Sundornagar

P7: There are around 4 Pharmacies in Shahid nagar

P4: There are two paramedics in Shahid nagar and Daudkandi

P3: When the diseases are critical we visit Daudkandi

P2: There are two homeopathy doctors in Dashpara.

M: Let me know the distance from community clinic to other facilities.

P1: From community clinic to Upazila Health complex is 20 to 30 minutes

P1: Daudkandi is 6 kilometres away from community clinic

P2: Shundolpur is 15 to 20 minutes away from community clinic by CNG (this is a 3-wheeler vehicle which uses compressed natural gas as fuel- commonly known as CNG)

P5: Mukti, Desh hospital is 5 to 10 minutes from community clinic.

M: Where do people in this community go when their children become sick?

P6: People mostly visit to community clinic for treatment

P3: People visit to Gauripur Upazila Heath complex

P4: People also visit to Sundolpur.

P2: In case of emergencies we go to hospitals and other clinics in Shahidnagar , Gauripur or Shundolpur

P1: We usually visit to community clinic

M: What do you know about medicines? Are you aware of different types of medicines? Do you know the difference between antibiotics and other medicines?

P6: We have a clear knowledge about antibiotics and normal medicines such as Calcium, paracetamol, eye drop etc

P3: People have a knowledge about Fymoxil , antacid, Anti-helminthic drug for the children

P1: We know that there are some medicines called antibiotics which have various types.

P4: We have a very clear knowledge about the variation of medicines.

P7: Everyone knows about antibiotic

P6: People are familiar to the term ‘antibiotic’

P4: People know that they have to be more careful while giving antibiotics to children.

P1: We know about Antibiotic.

P2: We have a knowledge about vitamin and mineral supplements, Metronidazole, few ointments because these medicines are available and provided from the community clinic.

P3: We have a good knowledge about antibiotic, its use, effects of irrational use, courses.

M: What type of medicines given in this community clinic?

P1: CHCP provides paracetamol

P2: CHCP provides medicine for deworming

P3: She provides Metronidazole syrup

P4: CHCP provides Vitamin D complex

P5: CHCP provides us cold, cough syrup, Eye drop, saline

P6: CHCP provides Amoxicillin , Fymoxil

M: When people receive any medicine, does the provider explain them to the correct doses?

P1: Yes, The provider strictly advice to complete the course of the medicine

P2: She advises patients to understand the importance of taking the full course of medications for 7 days. She tells us that if we do not complete the course, it could come back again.

P3: Yes, CHCP mentions how much we will take the medicine.

P2: CHCP also tells ‘do not forget to take medicine on time’.

P4: CHCP advises the doses correctly such as in how many spoons we will take “at what time”.

P7: Yes, CHCP explains the process of medicine taking to the patients

P1: CHCP inform us that if we don’t complete the course it could reduce the effectiveness of the drug in future.

P2: CHCP advices to take good food such as milk, egg, vegetables etc.

P1: Before providing the medicines, CHCP councils us about the medicines.

P7: Two of the CSG (Community Support Group) members have got training so they have very clear conceptions about it.

M: When people receive any medicines, does the provider explain them about sharing medicines?

P6: There is no chance of sharing the medicines because the CHCP never provides extra antibiotics.

P4: Sometimes we share general medicines such as paracetamol but we never share antibiotics.

P3: We do not get extra antibiotic, we cannot share to any one.

P7: Now a day’s people are much educated and aware that’s why they do not share antibiotics to other in their locality.

P7: Sometimes unconscious people share medicines with others.

P6: We keep the medicines in freeze for use in future.

P1: We do not get extra medicines, we do not have to share with any one.

P2: People usually share other general medicines but rarely share antibiotics.

M: When people receive any medicines does the provider explain them about leftover medicines?

P5: There are less chances of having leftover antibiotics, but we store other medicines at home in freeze.

P6: People generally keep Paracetamol to serve others in their community.

P2: She (indicating CHCP) advises us to complete the course.

P1: Some people throw the leftover medicines.

P7: CHCP provides 7-days medicines, there is no leftover antibiotics to share anyone.

M: If the supply of medicine at community clinic is insufficient then what people do actually?

P1: In case of insufficient supply of medicines, the CHCP informs patients about the scarcity of medicines.

P7: In case the medicines are not available, she refers the patients to the Upazila Health Complex

P6: CHCP provide a slip mentioning the name of the medicines to get it from Upazila Health Complex. TLC will provide the medicines from UHC.

P3: If the supply is insufficient the provider give us a partial prescription and tell us to come again when we have finished taking the given medicine.

M: Where do people in this community go to get medicines?

P6: We get free medicines from community clinic.

P3: From community clinic, if not cured, then people go to Upazila health complex where they need to pay 5 taka for ticket.

P1: Very few antibiotics are provided free from Upazila Health Complex

P4: We also get medicines from pharmacy.

P2: We can buy medicines from pharmacy too

P7: CHCP provides 7 days Antibiotics- the full course. In case the medicines are not available, she refers the patients to the Upazila health complex.

P5: We get medicines from community clinic

M: If you are not feeling well and you visit a health care provider but are not given any medicine, then what people usually do?

P6: People usually do not accept it.

P5: CHCP gives some medicines such as vitamins, saline and refer them to other health care provider.

P2: Some time they do not agree with the provider and get angry

P3: They go to other provider for further treatment

P6: Sometimes people like to visit private clinic as well

P7: CHCP advises them to take rest, not to do tension at all and patients agree with her.

M: I would like to know whether people usually follow the instruction of CHCP?

P1: Yes, all follow any advice provided by the CHCP in relation to medicines or the disease.

P6: Patients follow the advice given related to use of antibiotics.

P3: Yes, people follow her instructions

P4: Yes

P2: Yes we follow the instruction

P7: Yes we follow

M: I would like to know do people usually complete the course of medicines?

P6: The provider gives information on completing the course of the antibiotics to the patients.

P3: Some patients do not take full course medicines from CHCP because of poverty.

P4: People who are conscious usually follow the advice and complete the course of antibiotics.

P1: But there are many people who stop taking medicine when they feel better.

P2: Few of the community people also do not even complete the course of their children because they have lack of knowledge.

M: I would like to know whether it is easy or difficult to get medicines.

P6: It is easy to get antibiotics from various providers.

P2: CHCP provides antibiotic only when it is needed

P4: In case of other providers they prescribe the antibiotics and the patients have to buy them from the pharmacy.

M: Is a prescription always required?

P1: Yes, we need a prescription to get antibiotics from the pharmacy.

P4: If they can tell the name of the medicine then the pharmacists usually sell those to them.

P6: If they can recall the name and can tell the name of the medicine then the pharmacists usually sell those to them.

P2: Pharmacist usually sell paracetamol without prescription.

P6: The pharmacy can give the antibiotics to the patients when the patients take the empty blister to the pharmacy.

P3: If we can tell the name of the medicine then the pharmacists usually sell those to us.

M: What people usually do with the medicines that are leftover?

P6: They share leftover general medicines such as Paracitamol, Antacid.

P6: Some people bring those back to the Community Clinic

M: Do you know about what is ‘Antibiotic Resistance?

P4: No, I am not familiar with the term “Antibiotic Resistance”

P6: I have never heard the term “Antibiotic Resistance”.

P3: I have an idea about the bad impacts of irrational use of antibiotics but have never heard the term Antibiotic Resistance.

P1: No, not familiar with the term “Antibiotic Resistance”

P5: I have received information from the CHCP and also from the other providers on the adverse effects of not completing the course of antibiotics.
