# Supplementary material for: A process for developing a sustainable and scalable approach to community engagement: community dialogue approach for addressing the drivers of antibiotic resistance in Bangladesh
Source: BMC Public Health. 2020 Jun 17;20:950. doi: 10.1186/s12889-020-09033-5 (PMC7302129; doi:10.1186/s12889-020-09033-5)
Supplement: Supplementary file 3 — Additional file 3. CSG Members Female (3). Transcript of focus group discussion with female members of the community support group, region 3. [file 12889_2020_9033_MOESM3_ESM.docx]

| **Study Name:** **Community Dialogue for preventing and controlling antibiotic resistance in Bangladesh: Case for Support** | **Interview ID: CC3 Female FGD** |
| --- | --- |
|  | **Date of Interview:**  **17/04/2017** |

M = Moderator

P = Participant

P1: Cleaner

P2: Social Worker

P3: Cleaner

P4: Family welfare Assistant

P5: Health worker

P6: Teacher

P7: Student

M: Where do people in this community go?

P1: People of this community usually come to the community clinic first for getting their primary health care services.

P7: Poor people can afford to go community clinic as it is in the locality.

P2: People usually come to community clinic because they get free medicines from here.

P6: Sometimes they go to Malay bazar because once the patient might have been cured going there.

P2: People visit Private Medical Practitioner at Gauripur Union where the Upazila Health Complex is situated and when referred from CHCP.

P6: Sometimes they visit Desh, Mukti hospital at Mahananda

P3: They go to Upazila Health Complex after 3 pm when Community clinic closes.

P4: Sometimes we visit Comilla hospitals.

P5: We also visit some private hospitals at Gauripur

P6: We go to Dhaka when the provider refers us.

P1: In Dhaka we mostly visit Shamoly, Dhanmondi hospitals

P8: Sometimes visit India if the cases are critical and referred by the providers.

M: Do people visit traditional healer?

P1: Yes, people mostly visit there for sickness of their children

P6: Mostly visit for when children suffer from stomach upset, when the children are crying too much

P4: They visit for Infertility problem, repugnance of child, Pox treatment.

P5: People believe in traditional healer.

M: Which type of people mostly visit traditional healer?

P6: People mostly visit traditional healer for sickness of children.

P5: Traditional healer gave them Tabiz (it is a locket containing verses from the Quran and used by the traditional healer)

M: Do you have any pharmacy close to community clinic?

P4: Yes, we visit a pharmacy which is in Malay Bazar

P7: Pharmacy in Malay

P2: MBBS doctors sit in the pharmacy

P1: Yes, we have a pharmacy which is in Mahananda.

P6: There are eight to ten paramedics in Malay Bazar

P4: There are two paramedics in Mahananda.

P3: When the diseases are critical we visit these places

P2: There are two homeopathy doctors in Malay.

P5: There are two homeopathy doctors in Mahananda (Mahananda is not a village, it is a Bazar at Laxmipur village)

M: Let me know the distance from community clinic to other facilities.

P1: From community clinic to Upazila Health complex is six miles

P6: It takes 10 to 15 minutes to travel by rickshaw from community clinic to Upazila health complex.

P3: Malay Bazar is 5 minutes away from community clinic by CNG (this is a 3-wheeler vehicle which uses compressed natural gas as fuel- commonly known as CNG)

P2: Mahananda is 5 minutes away from community clinic by CNG

P5: Mukti, Desh hospital is 5 to 10 minutes from community clinic.

M: Why do people visit to homeopathy clinic or what kind of symptoms they have when they access these sources of care?

P1: Mostly visited for cold, cough, fever

P2: Sometimes they feel that allopath does not work well, so they visit homeopathy doctor for better treatment.

P4: People mostly visit (homeopathy clinic) for breathing problem.

P6: Mothers visit with their children with erythematous rash (new born baby).

M: Where do people in this community go when their children become sick?

P6: They mostly visit community clinic

P3: Gauripur Upazila Heath complex

P4: They also visit Malay Bazar.

P2: They also visit Upazila health complex to get better treatment such as supports during child delivery.

M: What do you know about medicines? Are you aware about different types of medicines?

P6: We have a clear knowledge about antibiotics and normal medicines such as gastric, fever, antibiotic syrup for the children (which is a suspension – mixed with warm water)

P3: People have a knowledge about painkiller, calcium, iron tablets for pregnant women, vitamin, saline, eczema ointment, skin problem ointment, eye drop, and vaccines for the children and mothers.

P4: If the provider feel that they need antibiotics then she give them that. It depends on the health condition of a patient.

P5: People feel that antibiotic works quickly.

P6: They believe in antibiotics.

P2: People are familiar to the term Antibiotic

P1: People mostly have knowledge about antibiotic. If someone do not understand, they keep the strips (of medicine) and come back to CHCP, she explain them again.

P6: People have to wait minimum three days before starting antibiotics

P1: Frequent use of antibiotics can weaken the immune system

P7: When people suffer from chronic disease, CHCP advises for antibiotic use and when the patients suffer from less severe disease, CHCP gives general medicine.

P1: People can differentiate antibiotics with other medicines after using.

M: When people receive any medicine, does the provider explain them to the correct doses?

P6: Yes, CHCP provides advice related to the use of medicines or antibiotics to the patients.

P2: Health care provider mark to the medicines using seizer with symbols.

P3: Yes, CHCP mentions how much they will take the medicine.

P5: CHCP informs the patient about the full course with fixed time.

P2: CHCP also mentions about expiry date of antibiotics.

P1: CHCP marks in the medicines to remind them (the patient) how to take the medicines

P4: CHCP advises the doses correctly, like ‘how many spoons they will take’, ‘at what time’.

P7: Yes, CHCP explains the medicine taking process to the patients

P1: The community people were given appropriate information by the providers about correct doses of medicines.

M: When people receive any medicines does the provider explain them about sharing medicines?

P6: The CHCP does not provide any information regarding not to share the medicines with others.

P4: We tak all medicines those CHCP provides us.

P3: WE do not get extra medicines, we do not have to share with any one.

P1: There is no chance of sharing the medicines because the CHCP never provides us any extra antibiotics.

M: When people receive any medicines does the provider explain them about leftover medicines?

P5: In case of leftover medicines most of the patient bring the medicines to the CHCP, she keeps the medicines to the community clinic to serve others.

P6: People generally keep Paracitamol to serve others in their community.

P3: Sometimes they bring the leftover medicines to the pharmacy to exchange it with other medicines they need.

M: If the supply of medicine at community clinic is insufficient then what people do actually?

P1: In case of insufficient supply of medicines, the CHCP informs patients about the scarcity of medicines.

P2: They wait for the supply of the medicines to the community clinic for 2/3 days.

P4: In case the medicines are not available, she (indicating CHCP) refers the patients to the Upazila health complex with ticket.

.

P5: CHCP provides 5 days Antibiotics- the full course. In case the medicines are not available, she refers the patients to the Upazila health complex.

P3: CHCP does not provide 5 days medicine when the supply is inadequate, she provides 2 days medicines

P6: The poor people do not go elsewhere to collect medicine if the supply is inadequate, they wait for the supply of the medicines to the community clinic.

M: From where people in this community go to get medicines?

P6: We get medicines from community clinic.

P3: From community clinic. If not cured, then people go to Upazila health complex where they need to pay 5 taka for ticket

P1: people also get medicines from Private clinics at Mahananda or Malay Bazar

P4: We also get medicines from pharmacy.

P2: From Upazila health complex they provide prescription and we need to buy medicines from pharmacy (Consulting fee – BDT 300 -1000)

P1: Antibiotics can be bought easily at the pharmacy if the name of the antibiotics can be mentioned. But we have to mention the milligram.

P5: We can get easily iron tablets, folic acid, vitamins from pharmacy.

P6: From community clinic, we get Paracitamol, Antacid, Metronidazole, Ointment for skin allergy, nasal drop, eye drop, Napa extra, and vaccine.

M: If you are not feeling well and you visit a health care provider but are not given any medicine, then what people usually do?

P6: They accept explanations or suggestions they receive from the provider.

P1: They follow CHCP’s instructions.

P2: They follow provider’s instructions such as taking rest.

P3: Some patients would get angry but when they get good instructions from CHCP they become motivated and follow the instructions.

P4: CHCP instruct them in good manners, so patients follow her advice.

P5: Every one follow her advice.

P7 : Yes, every one follow her instruction.

M: I would like to know whether people usually follow the instruction of CHCP?

P1: Yes, all follow any advice provided by the CHCP in relation to medicines or the disease.

P6: Patients follow the advice of antibiotics.

P3: Yes.

P4: Yes, we follow.

M: I would like to know, do people usually complete the course of medicines?

P6: The provider gives information on completing the course of the antibiotics to the patients.

P3: Some patients do not take full course medicines from CHCP because of poverty.

P4: Some patients do not take full course in his or her own interest because they think they would get better even if they take partial medicine. When they don’t get cured, then will take all.

M: I like to know whether it is easy or difficult to get medicines?

P6: It is easy to get antibiotics from various providers.

P2: The pharmacy can give the antibiotics to the patients, without prescription if the patient can mention the name of the antibiotic.

P4: If they can tell the name of the medicine then the pharmacists usually sell those to them.

M: Is a prescription always required?

P1: No. The pharmacy can give the antibiotics to the patients, if the patient can mention the name of the antibiotic, even without prescription.

P4: If they can tell the name of the medicine then the pharmacists usually sell those to them.

M: What people usually do with the medicines that are leftover?

P6: They share leftover general medicines like Paracitamol, Antacid.

P6: Bring those back to the Community Clinic

M: Do you know about what is ‘Antibiotic Resistance?

P4: No, not familiar with the term “Antibiotic Resistance”

P6: They have received information from the CHCP and also from the other providers on the adverse effects of not completing the course of antibiotics.

P3: I have an idea about the bad impacts of irrational use of antibiotics but I have never heard the term “Antibiotic Resistance” before.
