# Supplementary material for: A process for developing a sustainable and scalable approach to community engagement: community dialogue approach for addressing the drivers of antibiotic resistance in Bangladesh
Source: BMC Public Health. 2020 Jun 17;20:950. doi: 10.1186/s12889-020-09033-5 (PMC7302129; doi:10.1186/s12889-020-09033-5)
Supplement: Supplementary file 4 — Additional file 4. CSG Members Female (4). Transcript of focus group discussion with female members of the community support group, region 4. [file 12889_2020_9033_MOESM4_ESM.docx]

| **Study Name:** **Community Dialogue for preventing and controlling antibiotic resistance in Bangladesh: Case for Support** | **Interview ID:**  **CC4 Female FGD** |
| --- | --- |
|  | **Date of Interview:**  **04/05/2017** |

**Potential Intervention:**

M = Moderator

P = Participant

P1: Widow

P2: The CSG member

P3: The CSG member

P4: Teacher

P5: Young

P6: The CSG member

P7: Poor

M: I want to understand the administrative layers of this area i.e.; what comes first Union or Ward or village? Could you please explain it to me?

P2: The Upazila Parishad comes first, and then comes Union Parishad.

M: Then?

P2: There are Wards under the Union Parishad. The Union Parishad is consists of several wards.

M: What comes first under a Ward?

P2: Villages.

M: How many villages are there in a Ward?

P2: Mostly, three or two villages. Sometimes one village represents a Ward.

M: How many villages do you have in your ward?

P2: There two villages.

M: Do you have a small unit of your village such as “Para” or, “Mohalla”?

P3: Yes, we have.

P2: We have Paras in our village.

M: Okay. Considering this administrative breakdown, I want to learn about the community meetings that are held in your area on various issues? So, could you please, explain how these meetings are conducted?

P4: Meetings are conducted in every two months.

P3: Those are community support group meetings.

M: Let me clear it. Have you heard about courtyard meetings or village arbitrary meetings where people discuss various issues of the village?

P2: A couple of days ago villagers have discussed about building a road and setting up of electrical pillars within the village. Since this area is a hard to reach area, we often face load-shedding due to the falls of the electric pillar. The villagers meet together and discussed about this. These meetings mostly happen in the school.

M: What are these meetings?

P2: These are called courtyard meetings. These are conducted at our school or at the market.

P4: They make an announcement through the mike. People are asked to meet at the primary school to solve any problem related to the community.

M1: What types of issues are discussed in these meetings?

P4: People discuss about the reconstruction of the roads by the Government and how can they contribute to the development of the village. The village Member distributes the relief to the community people provided from the government. Sometimes, the community people sit together to discuss how they can benefit the poor.

P2: Since our community is situated in a remote area, we face difficulties in communication as the roads are not developed. We often organize meetings to discuss about fixing the roads. We discuss about the reconstruction of damaged roads, construction of new roads. Actually, communication is our major problem. In addition, we also discuss about constant electricity facility, distribution of relief, old age allowance from the government and sometimes about the clinic (indicating the CC).

M: Who organizes these meetings?

P4: Basically, the leaders of the village organize these meetings. They have an influence in the community. For instance, you called me to participate in the discussion. I listened to you and participated in the discussion today. So, while attending the meetings they take one or two individuals with them from each Paras ( a small unit of the village).

P5: Each of the Paras or Baris has a leader. For instance, 'X' and 'Y' Baries have individual leaders and they always organize the meetings together. In addition, we have male and female Ward Members and some famous individuals who are responsible for organizing meetings.

P5: If any leader cannot be present in the meeting, he or she would send a representative. Everyone attends the meeting.

M: Who usually participates in these meetings—I mean do they have separate arrangements for men or women? Or, do they organize meeting separately for men and women?

P2: Females usually do not participate. Members or the chairman and males usually participate in these meetings. However, sometimes if required, the female Ward Member participates in the meetings with some of her followers.

M: In this situation, both males and females participate together?

P2: No, they participate in the meeting but sit separately. Mostly, the males participate in these meetings. Usually, females don’t participate.

M: But, you told us that female ward Member participates in the meeting.

P2: Yes, she participates if required. Or, in case if any participant is absent, we ask her to participate in the meetings. We conduct community group meetings in every month. She participates in those meetings every time call her.

P4: I work at the village level. The people of this area are very friendly. If you ask for any help, they will be there immediately. But females usually, don’t participate in the meetings. However, whenever we feel to ask the teacher and the CHCP to participate they partake in any discussion.

P2: Whenever we call them (the CHCP and the teacher) they come and whenever the CHCP call us, we visit the CC. We provide support whenever it requires; for instance, a few days ago we bought a fan for the CC.

M: Okay. Could you please tell me how often these meetings occur?

P3: Usually, these meetings are organized twice or thrice in a month.

P4: Sometimes, these are held once in a month but mostly, twice or thrice in a month.

M: Usually, during what time of the day these meetings are conducted?

P2: In the evening.

P4: Mostly, these meetings are conducted at night or in the evening.

P2: Sometimes, meetings are conducted after the evening prayers. Meetings are also conducted to solve fights or conflicts amongst the inhabitants. In the case of other issues, such as reconstruction of roads, meetings are held at night.

M: Okay. Usually, how long these meetings are held?

P4: It depends on the type of the problem.

M: Could you please explain it?

P2: Usually, these meetings are conducted for 2 or, three hours. Suppose, if the meeting starts at 8 pm, it will finish at around 9 or 10 pm. When these meetings are conducted we discuss about various issues. We conduct these meetings in the school yard so that everyone can join.

M: Okay. Now tell me one thing. Are there any places where the meetings are conducted?

P4: Yes, meetings are also conducted at Eidgah (The holy place where Muslims pray during the EID) twice in a year during every Eid. We collect funds twice in a year during the two Eids for the development of the Mosque, Community Clinic and for the school. Besides, we also discuss various issues informally in every Friday after the midday prayers.

M: Do you conduct meetings elsewhere, such as in a yard of a Bari (the place where two or more families live in separate houses)?

P2: No, we do not conduct meetings in a Bari.

P4: We usually discuss village issues at the primary school.

M: Okay. Could you please tell me, what motivates people to attend these meetings? For instance, the leader of a particular area asked some people to join in a meeting if they are interested. So, what inspires them to participate any meeting?

P2: It is essential to participate in the meetings and if requires I would give my opinion to the discussion. In addition, I would also interrupt if it is necessary but I would think twice before doing this.

P4: Suppose, someone has decided to donate some money and my presence might influence the person for taking this decision. May be if I discuss with 4 more persons, they might contribute the same.

M: What is its purpose? I mean donating money or something like that?

P3: For the development of the village.

P4: Yes, this is for the development purpose. However, there are some people who participate in these meetings to know the issues that would be discussed in the meetings. This is just to be aware of the issues discussed in the meetings. Some people participate in the meeting to become acquainted with others or to become famous in the locality. But most of the people participate in the meetings as they are very conscious.

P2: Some people attend there to learn and some attend to participate in the discussion. You also find some people who participate to become famous and some participates to witness the situation.

M: One of you told me that usually, men participate in the meeting and women don’t. Why is that? Why females do not participate in the meetings? What was the problem?

P2: Actually, there is no problem. Women would participate in the meetings to solve their problems organized by women. This is to uphold equal rights in the society.

P5: Women are given proper respect in this area. It is not necessary for the females to participate in the meetings. What would they do in the meetings? Men are capable of solving any problem.

M: Yes, but I am interested to know why females do not participate in the meetings like men?

P4: Actually it depends on their interest. One can participate in the meetings if she is interested to take part. Usually, females attend general meetings.

P5: Actually, it is not difficult for them. Native women attend meetings whenever it is conducted in an area. They listen to the discussions held in the meetings. Usually, men inform their women about the issues that had been discussed in the meetings and thus the women feel that they would know about the discussions anyway. We attend the meetings when it is necessary. For instance, we have attended this meeting. Similarly, when meetings are conducted for any issues related to the females, we participate there.

M: Now, could you please tell me, if I want to inform every inhabitant of this area about an issue, in your point of view, what would be the best way or, how they can be informed?

P2: They can be informed through making an announcement from the Mosque. People will be informed of the time and place of the meeting such as primary school yard etc.

M: Is there any other way to inform people?

P2: Sometimes a person will be sent to notify every home.

P4: Yes, there is another way. The Ward Member will send somebody to inform almost everyone about the meeting.

P5: It depends. When it requires they will announce from the mosque or, someone will be sent to the families.

M: Okay. Now, I want to know about the health services. How do people of this area are provided health services and who are the providers?

P2: Health services are provided at the community clinic. We conduct meetings in every 2 months in this CC where we inform members about medicines. Then they inform others. Those who need medicines for their children and for other family members will visit the CC. We make an announcement before the doctor visits the CC so that everybody can avail the health facility.

M: As you said, what are these meetings?

P4: These are community support group meetings held in every two months and community group meetings are conducted in every month. We fix these meetings after coordinating with community support groups.

M: What issues are discussed in these meetings?

P4: Keeping everyone clean—such as washing hands of the children, keeping the environment clean and particularly keeping yourself clean are discussed in the meetings organized by the support group.

M: Okay, you have learned about theses health issues from the meeting. What do you do after that?

P4: Then we provide this advice to others and encourage them to visit the CC to know about these issues more precisely.

M: How do you encourage them or inform others about the issues?

P2: Sometimes we inform them in person and sometimes, we ask others to inform their neighbours about this. People are informed in this way. Then they discuss the issue among themselves.

M: Who provides the health information?

P4: The CHCP.

M: How does she provide the information? I mean how does she deliver the health information?

P4: The provider delivers health information verbally.

M: Does she use anything during the delivery of the health information? Such as posters or leaflets?

P4: Yes, she provides posters on various health issues such as night blindness, worms. Sometimes, we bring the posters to them.

P2: Who wants to know about the health issues will read the posters.

P4: Posters are provided to everyone.

P2: Sometimes, we show the posters to others.

P3: We inform our neighbours about the posters. Everyone asks about the meeting—what happened or what did you discuss today etc.—then we encourage them to visit the CC or if we carry the poster, we explain it to them.

P2: Then they show the poster to their children and explain everything, such as washing hands with soap before having meals or washing hands with soap after using toilet etc. There are instructions for the parents as well. For instance, in order to maintain a good health parents should wash their hands each time before feeding their children.

M: Now, please tell me what people prefer the most? Discussion or materials, such as posters or leaflets?

P4: People prefer discussion or explanation. They understand and prefer explanations. Everyone is not literate. They visit the CC because they like discussing with the care provider.

P1: They prefer pictures rather than verbal explanation.

M: What is it in the posters?

P5: I was afraid of taking anti-worm medicines. After seeing the posters, I am not afraid of taking anti-worm medicines anymore. It was mentioned clearly in the posters that how worms develop in our body and how to prevent it.

M: As you mentioned not everyone is literate, does the preference depends on their education level?

P4: Yes. There are some old aged inhabitants who cannot read. Children are attracted to posters.

P2: Those who are illiterate would not be able to understand the discussion properly. If there are posters with appropriate pictures they would be able to understand. It will be easier to explain the message.

P3: They will understand if you show the posters.

M: Okay. Now, please tell me, do you have any existing volunteer in this area who provides health education to the community people?

P4: Yes, we have one. She is ‘A’.

M: What does she do?

P4: She works as FWA (Family Welfare Assistant).

P2: We had another volunteer who used to provide health education to every family of this community. Now, she left the job.

M: Okay. Could you please, tell me what did she do?

P2: She used to assist pregnant women during their pregnancy. She used to provide condoms and information related to family planning.

M: Do you have any volunteers in this area?

P4: No.

M: Could you please tell how the volunteer was selected?

P2: She was selected by the Government. She was selected from the Upazila Health Complex.

M: Could you please tell me about the selection process?

P2: We don’t know.

M: Do you think people are happy with services provided by this volunteer?

P4: Yes. The market place is far from this area and we go there whenever we need something. We used to go Daudkandi frequently for the needs of our everyday life. We are happy with her services since she is here. And now we do not have to visit Dauidkandi regularly for certain health requirements.

M: Okay. Do you know who is supervising the volunteer?

P2: The Health Inspector supervises the volunteer.

M: Okay. Could you please tell me, what motivates her to work?

P3: She is serving the community.

P4: This is her job and she is paid for it. No one would work without payment. Let me tell you. Along with her job she sells medicines in the community and makes some profits from it.

M: Imagine, we want some volunteers from your community—may be one or two—who would spend 2 or 3 hours in every week to educate people about issues related to health. Do you have someone who would be interested to work as a volunteer? How can they be motivated to be involved in this?

P2: We are eight members here. You can choose one of us who can give you time.

M: Do you mean to select one member from the community support group as a volunteer?

P2: If you want, you can choose one of the members from the community group or from the community support group. Or, you can also select from the village people. We will help you in selecting one or two persons who would be able to do the work.

M: In that case, what would be the selecting criteria?

P2: The volunteer should be educated. He or she should have the ability to explain.

P4: Yes, they should have the ability to explain it to others.

P3: It is important to select someone who is acceptable by all and the community people would listen to him or her.

M: Okay. After selecting one or two individuals from the community, how they can be motivated to work for 2 or 3 hours in a week?

P2: Suppose we are eight members of this committee (indicating CSG). One of us could be selected as volunteer if other members approve—who would be capable of doing the job. The other committee members would monitor him or her. The volunteer should have the ability to communicate with people and will be able to provide time.

P4: I think you are asking to select someone from our village.

M: Yes.

P2: In that case, the ward Member would be able to help you to select someone from the village. According to the criteria, you have to ask the Member to select someone from the village who would be able to do the work. Tell him about the selection criteria.

M: So, according to you if the Member asks someone from the village to do this he or she will listen to him. If we provide training and other support, do you think it will motivate them?

P2: You have to train them everything. You have to tell the member that you will need two or three male or female volunteers for this activity.

P4: In order to involve in this activity, females would require permission from their husbands.

M: Okay. If we want two volunteers from your village for this project then what would you prefer? Male volunteers or female volunteers? Or, one male and one female volunteer?

P4: I think it would be better to involve one male volunteer and one female volunteer for this project.

M: Why do you think that?

P4: Because a female can handle females. Similarly, a man can handle men in the community. Females would feel comfortable to share any issue with another female. They would not prefer to discuss with males.

M: Volunteers have to discuss about antibiotics with the community people.

P4: Then male volunteers would be better who could explain it properly.

P2: There is no problem in involving males but whoever it is, he should have the ability to explain properly.

P3: There is no problem with males. The person should have the capability to do this.

P2: This is about medicine. He has to explain the full course, correct dosages and proper use of medicines. He should have an idea about medicines—which medicine is for what condition. No problem; youngsters can do it.

M: So, only male volunteers.

P2: Females can do it but no need to involve them.

P1: Females can also do it.

P3: Yes, females can also do it. Females will be needed here.

P4: Those who have the ability can be involved here.

P2: Let me tell you. It would be better if you involve female volunteers in this. They would communicate with every woman in this community and explain about the correct usage of medicines. Then the females of the each family would explain it to their husbands.

P3: She has to inform not only her husband but also other families in the community.

P5: Everyone should be informed.

P4: Females have direct access to families, males don’t have. Females can speak to other females directly. Man can discuss with another man. It would be appreciated if females are also involved in this.

M: So, you want to involve female volunteers.

P4: Yes. Here in families, females are responsible for giving medicines to all the family members in time. So, if the volunteers do not explain the correct usages of the medicines and how to follow the instructions of dosages then it (indicating the research) would not work. Males usually bring the medicines to the family but they are not concerned about providing instructions to other family members or observing the use of correct dosages.

M: Since we are going to involve to two volunteers for this project, what would you like to suggest?

P4: It would be better if you involve one male and one female volunteer in this.

M: And why do you think that?

P4: Socialization will be maintained. Females have access to the families of this community and they have the capacity to explain. We will involve them in relation to their capacity to explain. I think females have equal skills about explaining issues related medicines like men.

M: Is there any challenge in your community for the women to be involved in this activity?

P4: No, there is no problem.

M: Okay. Now, I want to know about the community group and community support group of your area. I will talk about this. Do you have community support groups for a particular area? For instance, support group-1 will cover this area or support group-2 will cover that area—did you divide them according to paras or mohallas?

P4: There are three community support groups.

M: And how many community groups?

P4: One.

M: Are these three community support groups allocated for particular areas such as paras or mohallas?

P3: No, they are allocated for the whole village.

P4: There is a separate section for ‘Nandonpur’.

M: What is Nandanpur?

P4: It is a village. We have members in our support group from ‘Nandanpur’. They will take responsibilities for their village. Similarly, we have members from ‘Bhajra’ (this village) in the support groups and they will cover this area.

M: Okay. Now, tell me who are the members of the community group?

P4: The Ward Member. He is the president of the community group.

M: Who else are involved?

P4: The community group is comprised of a president, vice-president, treasurer, young, teacher etc.

M: Who is the vice-president?

P1: We have a former Ward Member who is the vice-president.

M: Is there anyone else in the community group?

P4: The CHCP (health professional).

M: Now tell me about the community support group? Who are the members?

P4: Community support group has a service holder, teacher, young, poor, widow etc. I can’t remember the rest.

M: How many members are there in the community group?

P4: There are 17 members in the community group.

M: And how many members in community support group?

P4: The community support also comprises of 17 members. Each of the three community support groups consists of 17 members separately.

M: Now, I am interested to know about the how the members of the community group were selected. Who is responsible for selecting the members?

P4: The chairman and Ward Member are mainly responsible for selecting them. The president will always be the Ward Member. In addition, the health inspector, teachers also play role in selecting the members of the community group. We organized a meeting and then we decided to select them according to their ability to work.

M: Now, tell me about the community support group. How the members were selected?

P4: The Community group members then select the members of community support group. They select them in relation to their ability and willingness to work for the community. They make a list from the community and then select them according to their ability.

M: Now please tell me, who has given the selection criteria; that is, the member would be the president, a young, teacher etc.?

P4: The CHCP.

M: Where did she get this?

P4: She had received it from the Upazila Health Complex.

M: Okay. How much time do they spend for the welfare of the community clinic? How much time do the members usually give for the activities of Community group and community support group?

P4: Whenever you ask them to come for any activities related to the community group or support group or for the community clinic, they will come.

P2: They give time whenever it requires.

M: Are there some members who are particularly active and some are less active?

P2: No. All the members are active and do their duties according to their convenient time.

M: How is that?

P4: Suppose, you asked someone to do something and you gave him time. He will try to do this within the time frame.

M: Are there any member who is less active?

P4: We don’t have any member who is less active. For instance, you asked all the members to participate and we are all here today.

M: Okay. Now tell me, who supervises and monitors the activity of the community support group and community group? For instance, organizing and participating in meetings, solving any problem of the community or, whether they are doing the work properly or not etc.

P2: The Ward Member supervises or monitors the activity of the groups. The member observes their activity that is whether the members are participating and conducting the meetings or not; or, whether they are doing the work properly or not and whether the health professional is coming regularly or not etc.

P4: The Health Inspector also supervises the activity of the community group and community support group. Sometimes he reminds them about their activities.

M: Okay. Could you please tell me about the meetings that are conducted in the community? Usually when these meetings are held and what issues do they discuss?

P3: Community support group meetings are held in every two months.

M: Community group meetings!

P4: Community group meetings are conducted in every month.

M: What issues do they discuss in these meetings?

P4: When they sit together they discuss about various issues and inform every member about current activities. For instance, last month anti-worm tablets were provided from here (indicating the CC). Iron tablets were provided to the young girls and pregnant women. They were also advised about hand washing techniques. Sometimes they discuss about the cleanliness of the environment.

For instance, a few days ago we have cleaned the grasses from this setting with the help of a volunteer.

P2: We have collected a small fund for this purpose. The community members provided a fan to the community clinic by collecting funds from all the members (indicating CG and CSG members). Whenever you ask them they will help and support you.

M: So, could you please tell me, what is the link between the community clinic, community support groups and community group? What works between them?

P4: The CHCP.

M: How is that?

P4: She informs everyone about the timing of the meetings and the place where the meetings would be conducted etc.

P2: Mostly the meetings are conducted at the CC. The community group and community support groups discuss any problems related to the CC and provide support in solving the problems.

M: Okay. I have learned from your conversation that the community support groups are very active here. Could you please tell me if the community support groups face any challenges during following their responsibilities?

P2: No. They never face any challenge.

P4: Sometimes, the supply of the medicines interrupts—particularly calcium tablets.

P2: Now, the supply of the calcium tablets has stopped.

P4: This is the problem.

M: Did you take any initiatives in solving this problem?

P3: Since this area is low and surrounded by water, skin diseases are very common here. We always need medicines for skin diseases.

P2: The government has stopped providing calcium tablets. We asked the CHCP about this. She said that she would provide the medicine when it arrives.

M: Did you take any step to solve this problem? For instance, contacting with the Upazila Health complex for this purpose.

P2: No, we did not do that.

P4: No, we did not do that.

M: Suppose, we have involved a volunteer from your village who is providing health education in this area. So, in your opinion, who should supervise the activities of the volunteer such as conducting meetings, providing health education etc.?

P4: The CHCP or the Health inspector.

P2: The president of the community group.

P4: Yes, the Ward Member could also supervise the volunteer. We the members can also do this. We can also provide advice or can help the volunteer.

M: Which members, community group or, community support groups?

P4: Community support group members are the main. Actually, they are actively taking care of everything.

M: In that case, do you think that the CSG members could keep the records of the activities of the regular meetings and feed them back to the community clinic?

P4: I think the support group can do this. Since they (CSG members) work at the field level, it would be better to involve them for this.

M: So, if we link the CSG between the facilitators of the regular meetings and the community clinics, do you think it would work?

P2: It would be a good idea.

M: How do you think it would work?

P3: They CSG members can observe or can supervise the regular activities of the volunteer. The volunteer will do this properly.

P4: Yes, the volunteer would become more serious about his or, her responsibility.

M: Thank you very much for your valuable information.
