# Supplementary material for: A process for developing a sustainable and scalable approach to community engagement: community dialogue approach for addressing the drivers of antibiotic resistance in Bangladesh
Source: BMC Public Health. 2020 Jun 17;20:950. doi: 10.1186/s12889-020-09033-5 (PMC7302129; doi:10.1186/s12889-020-09033-5)
Supplement: Supplementary file 5 — Additional file 5. CSG Members Female (5). Transcript of focus group discussion with female members of the community support group, region 5. [file 12889_2020_9033_MOESM5_ESM.docx]

| **Study Name:** **Community Dialogue for preventing and controlling antibiotic resistance in Bangladesh: Case for Support** | **Interview ID: CC5 Female FGD** |
| --- | --- |
|  | **Date of Interview:**  **07/05/2017** |

M = Moderator

P = Participant

P1: Family welfare Assistant

P2: Social Worker

P3: Member

P4: Service holder

P5: Health worker

P6: Teacher

P7: Student

**M: First of all, we want to know, when the people of this area getting ill then where are they usually going for treatment?**

P1: People of this community usually come to the community clinic first for getting their primary health care services.

P6: People usually come to community clinic for consultation

P4: People usually come to community clinic for treatment and advice

P5: Sometimes they go to paramedics in kushiara bazar

P2: People visit Private Medical Practitioner at Gauripur Union where the Upazila Health Complex is situated.

P6: Sometimes they visit Homeopath in Raipur bus stand

P3: They go to Upazila Health Complex in Gauripur Besides this, people also visit FWC (Family Welfare center)

P4: Sometimes we visit MBBS doctor in Gauripur bus stand

P5: We also visit Traditional healer in Raipur bazar

P1: People visit Upazila health complex for the delivery of pregnant women.

P2: When the diseases are at critical stage, people usually visit to City hospital at Gauripur Bazar.

**M: Which age people come in this community clinic most?**

P1: Starting from a child to old, all kinds of people visit this center for treatment.

P3: Now the pregnant women also come here.

P4: Now a days, there are more pregnant women here.

P2: People usually come here for getting free medicine.

**M: Do people visit traditional healer?**

P1: Yes, people mostly visit them for the treatment of infertility.

P6: People visit them for the treatment of measles

P5: People believe in traditional healer. They usually go to them for the protection of their child from evil eye.

P2: Traditional healer gave them Tabiz (it is a locket containing verses from the Quran and used by the traditional healer)

**M: Why do people visit to homeopathy clinic or what kind of symptoms they have when they access these sources of care?**

P6: Yes, those who are not satisfied with the treatment of allopathic medicines they go to homeopathy doctor.

P1: People visit there for the treatment of boil, tumour

P2: People mostly visit for the treatment of menstrual problem.

**M: The people who usually come to the community clinic, do they go for homeopathic treatment?**

P4: Yes, they go. If they don’t get well by taking the medicine from Community clinic, then they go to the homeopathic doctor.

**M: Let me know the distance from community clinic to other facilities.**

P1: From community clinic to Upazila Health complex is 4 to 5 kilometres.

P3: Kushiara Bazar is 1 and half kilometers away from community clinic

**M: Where do people in this community go when their children become sick?**

P6: They mostly visit community clinic

P3: Gauripur Upazila Heath complex

P4: They also visit Kushiara Bazar.

P2: They also visit Upazila health complex to get better treatment such as supports during child delivery. Pregnant mothers go to Upazilla Health Complex more because there are gynecologists.

P1: Usually go to Community Clinic for preliminary treatment and advices

**M: What do you know about medicines? Are you aware about different types of medicines?**

P6: We have a clear knowledge about antibiotics and normal medicines such as cheap and costly medicines.

P2: I don’t get your point.

P3: We know the term antibiotic. We classify them as antibiotics and other general medicines which have no side effects. But most people do not understand the difference between various drugs.

P1: Those who are educated have the idea about the difference. They can understand which is normal medicine and antibiotic.

P4: I know that one type is vitamin and other type which is stronger than vitamin named antibiotic

P5: People know that costly medicines are antibiotics which are usually prescribed for serious conditions

P2: We believe that Antibiotics have more power than other medicines.

**M: Do general patients understand why they are sometimes given antibiotics and sometimes other medicines?**

P1: Some people understand it. Rather, if the doctor prescribes antibiotics, they are afraid that they have something bad that’s why doctor gives them powerful medicine.

**M: Where do you usually collect these antibiotics?**

P1: The community clinic authority gives antibiotics to the patient. In addition, Kushiara bazar also available to buy antibiotics.

P2: MBBS doctors of Raipur bazar also prescribe antibiotics.

**M: When people receive any medicine, does the provider explain them to the correct doses?**

P6: Yes, all the health service providers’ advice people to complete the course

P2: CHCP tell us that if we don’t complete the course, we will have to start it from the beginning

P3: The people get advices about the correct dosage of antibiotic

P5: MBBs doctor gives prescription and write down the instruction.

P4: Others (drug sellers at kushiara bazar) cut the medicine strips according to the dose. But they verbally demonstrate when a medicine will be taken. Since MBBS doctors have written the rules for taking medicines.

P6: CHCP cut the medicine strips according to the dose those who do not understand the medicine process then he makes them understand through oral explanation.

P7: Yes, CHCP explains the medicine taking process to the patients.

P1: The community people were given appropriate information by the providers about correct doses of medicines.

**M: Well, suppose the doctor gave you medicine for five days. Three days after eating, you feel better and you did not eat the medicine for the next two days. Then what do you do with that medicine?(left over)**

P2: The rest of the drug is stored in the house and later if the same disease causes the medicines are then taken again.

P1: Return to the shop where I took the medicine. We ask the doctors, what do I do with the rest of the medicine now?

P2: When buying medicines from the pharmacy, they returned the medication again.

P5: We usually keep leftover at our home but we are advised to discard the leftover medicines if the date are expired.

P6: Some of us go to pharmacy to exchange them with other medicine we generally need.

**M: Does everyone return the unused medicine to the drug seller?**

P2: Those who understand, they return, but those who do not understand keep them in the house. Due to the long stay in the home, medicines are expired.

P3: It should have eat all the medicines. There is no rule not to take medicine . The person who did not take the medicine properly, could not understand why he was given medicine.

**M:** **Suppose, for a particular disease, the doctor gave me antibiotics. Now, if someone around me had similar problems, I asked him/her to take this medicine. Does it happen like this?**

P1: No, no, no….that doesn’t happen in case of antibiotics.

( Rest of the participants agree with P1 statement)

**M: When people receive any medicines does the provider explain them about not to share medicines with other people?**

P6: The CHCP does not provide any information regarding not to share the medicines with others.

P4: No one advices not to share the left over medicines because they provide the medicines of full course that should be completed

P1: CHCP advices us not to share any medicines with others.

P2: Yes, CHCP advices us not to take medicines after expired.

**M: Are there any pharmacy near the community clinic?**

P1: Yes, there are.

**M: Then people can buy antibiotics anytime from there-**

**P1:** The owners of these pharmacies are mostly paramedics. The villagers call them doctors. They write different medicines by examining people. So there is no medicine available from there.

**M: Now you tell me, if the supply of medicine at community clinic is insufficient then what people do actually?**

P1: In case of insufficient supply of medicines, the CHCP informs patients about the scarcity of medicines.

P4: CHCP tells them to come when the supply will be adequate

P2: If the supply is inadequate then visit to pharmacy. There is no lack of pharmacy here.

P3: CHCP provides 5 to 7 days Antibiotics- the full course. In case the medicines are not available, she refers the patients to the Upazila health complex at Gauripur.

P4: CHCP refers to Upazila health complex when the supply is inadequate

**M: It seems that the supply of medicines to community clinics is a little less. And the doctor gave you medicine for 7 days. But from the community clinic you have been served with 3 days of medication and been told that after taking those medications, you will take the medicine for the next 4 days. In this situation, do people again take the rest of the medicine?**

**P1:** Many come, many do not come. Those who understand that the medicine course will be finished they come. And those who are ignorant, only take those three days of medicines and do not eat the rest of the medicines.

P5: I did that once. I was given 3 days of medicines and the rest of the medicine was supposed to be brought later. But I feel better after taking medicines for 3 days so that I do not go to the community clinic to bring the rest of the medicine.

**M: Well, do you know if someone else did such a thing like you?**

P5: I don’t know about others.

**M: Now tell me the name of some place from where the medicines are available without prescription**.

P2: There are some pharmacies who give medicines to people without prescription.

P1: In those pharmacies, if we can say the name of the medicine, they give us medicine without prescription.

P3: If I said, I am not get well from fever after taking paracetamol then the drug seller will give antibiotics for recovery. Actually they want to sell drugs because that is their business. I saw this situation of drug shop in my own eye.

**M: From where people in this community go to get medicines?**

P6: We get medicines from community clinic.

P4: From community clinic.

P1: People also get medicines from Private clinics at Kushiara bazar

P6: We also get medicines from pharmacy.

P1: Antibiotics can be bought easily at the pharmacy if the name of the antibiotics can be mentioned.

P5: We can get easily paracitamol, vitamins, saline, iron tablets, calcium from pharmacy.

P3: We can get easily from paramedics at kushiara bazar.

**M: If you are not feeling well and you visit a health care provider but are not given any medicine, then what people usually do?**

P1: The people don’t accept the situation, they think that CHCP is not giving them antibiotics intentionally.

P2: People get angry and insist for medicines again and again

P3: People get angry and insist for medicines again and again

P4: CHCP instruct them in good manners, so patients follow her advice.

**M: I would like to know whether people usually follow the instruction of CHCP.**

P1: Yes, all follow any advice provided by the CHCP in relation to medicines or the disease.

P6: Patients follow the advice of antibiotics.

P3: Yes.

P4: Yes, we follow.

P5: Yes, those who are conscious, follow the instructions of CHCP.

P2: Those who are literate follow the instructions.

P6: Patients usually follow provider’s advice of taking medicines in correct dose.

**M: I would like to know, do people usually complete the course of medicines?**

P1: I stopped taking antibiotic in the 3^rd^ day of my course because I felt better and thought that there is no necessity to take farther dosage.

P2: Few careless and illiterate people don’t compete the course

P3: Illiterate people do not complete the course.

**M: I like to know whether it is easy or difficult to get medicines.**

P1: It is easy to get medicines from Community clinic. It is given to us for a period of 5 days.

P2: From Upazila Health Complex they will give a prescription

P3: From pharmacy very easy to get medicines

P4: If the patients mention the name of the antibiotics or can tell their symptoms the village doctor easily gives them antibiotic because they are the owner of the pharmacies.

**M: Is a prescription always required?**

P1: No. The pharmacy can give the antibiotics to the patients, without the prescription if the patient can mention the name of the antibiotic.

.

P3: If someone tells the pharmacist their symptoms then they provides medicine

**M: What people usually do with the medicines that are leftover?**

P4: They shared leftover of general medicines

P1: It rarely happens in case of antibiotics

P2: They mostly keep left over of other medicines rather than antibiotics.

**M: Do you know about what is ‘Antibiotic Resistance?**

P1: No, not familiar with the term “Antibiotic Resistance”

P5: We have no idea about antibiotic resistance.

P4: I have never heard the term “Antibiotic Resistance” before.
