# Supplementary material for: A process for developing a sustainable and scalable approach to community engagement: community dialogue approach for addressing the drivers of antibiotic resistance in Bangladesh
Source: BMC Public Health. 2020 Jun 17;20:950. doi: 10.1186/s12889-020-09033-5 (PMC7302129; doi:10.1186/s12889-020-09033-5)
Supplement: Supplementary file 6 — Additional file 6. CSG Members Male (1). Transcript of focus group discussion with male members of the community support group, region 1. [file 12889_2020_9033_MOESM6_ESM.docx]

| Study Name: Community Dialogue for preventing and controlling antibiotic resistance in Bangladesh: Case for Support | Interview ID: CC1 male FGD |
| --- | --- |
|  | Date of Interview: 09/04/2017 |

M = Moderator

P = Participant

P1: Secretory of CG group

P2: UP Member

P3: Chair of CG group

P4: Imam

P5: Village

P6: land lord

P7: Chair of CSG

M: It is not possible for health ministry to solve the problem. We will actually discuss about the medicines and supply of medicines that will be provided by the health ministry. Moreover, all these medicines are not same in nature. Presently, government is working on antibiotic. There are thousand types of medicines, isn’t it. Do you know here we provide around 33 types of medicines? I might not talk about these 33types of medicines either. So our brother will talk about a very few number of medicines. If you allow us we can start. But if any of you want to give us your opinions, doesn’t allow us to write your name in our research, we assure you, we will not do it. We can replace your real name by using imaginative name like ‘A’, ‘B’ or we can use any number.

P1: Thank you. Have you done? Can I tell something? He is our member. He is busy with his own office so that he can’t devote his time to any government work. I am also from same home. We are cousins. I am the chairman of supporting board. But he(CHCP) can meet me whenever needed. I usually come here with my children. Specially, I come here with my grandchildren to take the medicines. Our community center is situated at Bhuiyanbagh. I come here from Baghdak. Baghdak is approximately 10 and half kilometer of 7 miles far from here. Usually 60 to 70 patients come here per day; most of them are women (Mother) and children. I have noticed that, whatever medicines we are getting from here are good. These medicines have positive reactions, so far I know. Specially, I don’t buy any medicines for fever, cold, cough from any doctor. I usually take those from him (Pointing someone). First time I took those from him and found out these works better. So I take these from him. Afterwards, I have checked the statement and found out these have come here from Batakandi. What is our responsibility? IS it to advise someone, to say someone to go to clinic, to let someone know that here has good medicines? If we go to doctor we have to pay 300 taka visit fee. So we prefer to come here, so far we have noticed. We can say what we actually know. We don’t know anything additional than that. Now you have come here to brief us about good and bad. He has told about different types of antibiotics, some of those are for fever, for wound, for kidney infections and so many other infections. Some of those are Amoxicillin, Ciproxcin, Ciprofoxin and many other groups. Is it right? But all these antibiotics do not work better. Isn’t it? So you should provide us all these information as it is written here. You can discuss with us in a way so that we can understand. We all are with you and we are looking forward to work with you in future. We want to response at your call. Thank you.

M: Actually we will discuss about it. We wanted to talk about it. But we have few formalities before we begin. So we are trying to let you know about what we r working with, for whom we r working and what our goal is. Now the fact is whatever information you have just provided us, he also provided the same, which means this is our center of discussion. Now if we tell all these things earlier, then we might lose our consistency. So we want to begin from this point at a same flow in a consistent way. So we want to maintain some formalities before we began. Before we start we want to let you know that we value your time and opinions. Your times, opinions and experiences are very much important to our research. The reason we value your opinions is, we want to write a report to government from here. Something which is the real scenario of here. Have you got my point? Now I would like to request you one thing that, when we will talk each and every one has the opportunity to talk. So please do not that if someone is speaking then I don’t need to say anything. We don’t want anything like this, because every person’s experiences are different from another. As you have experiences as a member, he has experiences as a leader. He has experiences, you have experiences (point to the crowd). Everyone has different experiences, so everyone’s opinion also different. We do not need any particular person’s opinion rather we need everyone’s. Whoever knows anything he will tell, and we will give the equal floor to express everyone’s opinion, so that we will not miss any information. He has told an important thing that if someone participate in this discussion and tell his opinion after that if he thinks he has missed anything which could be important to the research then we are providing an address. You are free contact here anytime for any kind of quarry or advise. We have signed a copy of what he has said with us. We have made a mistake that we haven’t provided the copies to you. We should do it earlier. So do you want to keep the copy with you? Please give our signed copy back to us. So that you will be clear from your side and so do we. Alright? And there is a way to contact between us. If you need to ask anything or need to know anything or anyone have any kind of advice, you r welcome to contact with us. And so far I know if you talk to CHC of you can ask anything to CHC. After I joined this organization I have come here again and again and we are working on different issues. Today whatever services you are getting from this community clinic either that is any treatment or other than treatment, if you go there you will find a chart. We have made this chart. He is providing medicines to your children according to that chart. You are also collecting the medicines properly. We have provided them a training according to our government order. We did it because government ordered us to do so. So whatever we are doing here now everything is for your betterment. We have come here to ensure that this community clinic will provide you better services and roper treatment. With that we also thank you to come here. So here I begin, hopefully you will be able to read this projector.

Well, first we would like to know which type of services you require from here. We want to discuss about all of the health treatment we are getting from here. To do this first we will draw a map which will tell us about the places from where these community people are getting the health services. We need your cordial help to do this. Suppose it is a road, if it is our community clinic (pointing on monitor) okay. Where else do the people usually go for health services? So you need to find it out and help me to know about it.

P2: Is it government or non- government?

M: Everything. It is not important whether it’s government or non- government. It can be private also.

P2: Suppose I have come here for treatment. But doctor said treatment is not possible here. So he will refer me to another hospital. That might be Gouripur hospital or clinic.

M: Yes, that can be. But I want to know is, he (pointing someone) might go to Dhaka, but all of us will not go that far.

P3: If anybody comes here for initial treatment, after that he might go to Gouripur Health Complex. Initially he will be sent there.

M: How far that Health Complex is from here?

P3: Approximately 2 to 3 kilometer from here.

M: Which direction from here?

P3: Diagonally. It is straight diagonal way from here.

M: This corner (Pointing the place)

P3: This corner (Pointing the place)

M: Suppose it is upazila Health complex okay. We point it as UHC. How long will it take to arrive at community center from that place?

P3: By walk 1 hour. By CNG 20 to 25 minutes or by rickshaw it will take approximately half an hour.

M: Here we have found a UHC. Now as the location of this community clinic, more clearly the location this community clinic covers to provide treatment, we can assume that these area people will not go to Chandina to take treatment.

P3: No

M: He will definitely go to Tarail area to provide health services. People from Tarail area come here and the also go to Health complex. Where else can he go for treatment? This might be Aiyurvedic practitioner or a pharmacy. What else this might be?

P3: We have a clinic at Gouripur. Gouripur is this side. Direct this way.

M: Direct this way okay. How long will it take to reach there? Is it a government clinic or private?

P3: Private

M: The name is Gouripur. How long will it take to reach?

P3: It will take 15 minutes. If we take CNG or rickshaw it will take 20 minutes. We have everything here.

M: How long will it take to go by walk?

P3: By walk it will take 1 hour.

M: I have found 2 things over here. 1^st^ thing we have got a government hospital here which is pretty near and the second one is a private clinic. Is there any pharmacy in this area?

P3- P4: We have pharmacy at bazar

M: Which direction is it?

P3-P4: this one also beside the straightway.

M: this is pharmacy. How long am I taking to go there?

P3: you can go by walk or CNG or rickshaw. By walk it will take 10 minutes.

M: By CNG?

P3-P4: CNG will not go.

M: Will rickshaw go?

P3: By rickshaw it will take 5 minutes.

M: So I have found pharmacy. Is there any Paramedic or anyone like this who will provide health services?

P4: Dr. Mujibur Rhaman, he is a Paramedics.

M: Where his chamber is?

P4: From here to sarkari polli road. In this place (Pointing the location)

M: In this place.

P4: Paramedical doctor’s chamber.

M: Paramedic?

P4: Yes

M: Okay Paramedic. How far is it?

P4: It will take 10 min by walk from the community clinic.

M: How long will it take by CNG or anything else?

P4: By CNG it will take 5 minutes.

M: By rickshaw it will take 10 minutes right.

P4: Something like that.

M: So I take everything is okay for now.

So I have found here some important things. I have found pharmacy and paramedics. Do you have village doctor?

P3-P4: No we don’t have any.

M: There is no village doctor.

P3: No

M: So I have enlisted 4 ways from where people can take health treatment. Is there anything else like who is working under this community clinic location?

P4: Gouripur, Mujib market, Gouripur bazar or Health complex all these are 3 main places. 95% out of 100% people will go there. Rest of 5% is Shahid nagar. Shahid nagar also part of this.

M: I would like to clear one thing. Which 5% are you mentioning here?

P4: Shahid nagar is 5%.

M: Where is this shahid nagar?

P4: Straight West direction from here. It is just in the corner of Gouripur hospital.

P3: We consider our locations according to East West North and South. Form here, this corner, West- South corner or South- East corner.

M: How do you call this place then?

P4: It is Shahid nagar or Chatgaon.

M: Why do consider Shahid nagar as this kind of place?

P4: This one is beside the Trauma center. There are some doctors doing their business over there.

M: Is it a Clinic?

P4: No, it is a bazar. Here have some shops and beside these shops there are also some pharmacies. Some of doctors will sit here, some paramedical doctors.

M: Is it a pharmacy?

P4: Yes, it is.

P5: sometimes we see stations at bazar right.

M: So precisely, we can consider it as pharmacy.

P4-P3-P5: Yes, we can.

M: What is the distance from here? How far?

P4: The distance is same as Gouripur hospital from here.

M: Same distance.

P4: Yes, same distance only if you go by walk. If you go by CNG or rickshaw it will take bit more time then.

M: Do you agree that all these community clinic area people can get treatment from these places except community clinic? Is there any other places so far you know?

P3: Suppose the people out of community clinic they will come here for initial treatment. If they need furthermore treatment then they will go outside.

M: May be my question is not clear to you. Obviously community clinic is in the first place. But the thing is, we know we are providing enough treatment from community clinic and people are coming here for it. Are there any other places or more clearly are there any other potential places from where people can get treatment? We are trying to find out all these types of places. Like, community clinic is providing health services to these locations. Are there any other places from where people can avail the treatment facilities? Is there any possible area? Is there any particular place where people can go?

P2-P3-P4: In Gouripur there are more than 20 clinics. Along with diagnostic center there are around 50 clinics. The number can be more than 50. We have hospital as well as diagnostic center. The bottom line is there are more than 50 clinics near to this Gouripur road. We have diagnostic centers, primary clinics, hospitals, 4-5 paramedical centers and 1 homeopath.

M: Are you mentioning about 1 homeopath?

P4: Yes, 1 homeopath.

M: How many homeopath do you have here?

P4: we have 1 homeopath here. If we count then we have plenty in number. Shadhona, homeopath, ayurvedic, Humdard etc.

M: Shadhona, Humdard. What else have you said? Village doctor and homeopath. Do you think all these are just available for you or is it quite enough? So, let’s get into our next topic. Now we have an idea about the places where this area people usually go to get health services. We have idea about the potential places where people may go. Now my question is about those potential place. Why do people have only options to go there? Why don’t they go?

P5: There are plenty of reasons. They got to do X-ray, pathological test and they need a report. Doctor can suggest any medicine only. Last week we have seen that doctor referred a patient to Gouripur for TB test. From there they will collect the report. Then they will be sent to BRAC to collect the medicines.

M: Do people take health service from BRAC also?

P5: Yes, Only for TB. This treatment is free of cost. They will provide medicines also.

M: So people go to BRAC for TB test only. Do they give any treatment?

P6: Sometimes they do it free of cost.

P5: They provide financial support also. Sometimes they offer for eye treatment where patients can test their eyes with no cost or only they got to pay 30 taka. They arranges it in our educational or any private organizations. They usually arrange it in 1 or 2 months interval.

M: I would like to know 1 thing. How far the community clinic is?

P4: Approximately 1 mile or 1 and half kilometers.

M: How long will it take by walk?

P4: By walk it will take 30 minutes. It is on the way of Shahid nagar.

M: How long will it take by rickshaw or CNG?

P3-P4-P5: Here CNG or rickshaw cannot run on main road. Form main road it will take 5-7 minutes’ walk.

M: Again I am back to you uncle. Uncle has mentioned some reason why they will go there. What were those?

P4: Yes, patients come here, mostly they are children and women. Men usually don’t come. There are a lot of diabetic patients. If anyone come here in case of emergency chcp will check the diabetics by using a stick. By strips he checks it. So the patients can have an idea about it, that’s all. Then here they don’t have any facility for some other pathological test like blood, urine, or X-ray, echo etc. Where do people go then? The only place they can go is Gouripur. Doctors send them to Gouripur for checkups. If its not possible to handle or treat that patient then they will be transferred to Gouripur hospital. If the condition of patient is even more serious then they will be sent to Dhaka at PG hospital, Dhaka medical college etc. Here we can get the primary treatment for a time being. They are not able to give us any crucial treatment. There are enormous testes. So for any kind of test patients will have to go other than this place. Disease and the severity of disease will be recognized by different kind of tests and diagnosis. To do this we initially go to Gouripur hospital for 2-3 types of blood test, ultrasonography. Sometime all these test is not possible there. In that case we have to go other places. That time Gouripur clinic or any other specialist is the last hope. People go to specialist when they suffer from crucial disease. But the problem is most of the time these crucial report will not be right if we do it from here. In that case doctors will refer the patients to Dhaka. Patients will follow the suggestions as it is. From my knowledge I know PG hospital is good for diagnosis, treatment and the report also reliable. They charge even low cost. Dhaka medical college hospital also good for these. But doctor suggests the clinic as their own wish and the patients must have to follow it.

M: Does anybody want say anything but the things they have already said?

P6: That’s all actually whatever they have said about community clinic. There is a problem in community clinic. Suppose today someone’s delivery. But there is no arrangement for food or accommodation for the patients or visitors. So they are bound to go to private clinic. Cesarean operation or any kind of orthopedic test like X-ray to do all these there are no other places than private clinic. In this clinic we get little bit treatment for the cold, fever these types of light diseases. Some special test like ultrasonography for kidney disease, eco for cardiac issues, MR or anything like these are not available in this clinic. So doctor refers patients to Dhaka medical, or Padma, or Modern all these private clinics.

P7: I want to add one thing that community clinic has not provide us with complete treatment. This one is providing normal treatment for cold, fever or sometimes some treatment for children or women. People can get facility to test TB only. The way u are asking questions will not help you with concert answer. If this community clinic would have provided the complete treatment it could have been a complete hospital. Community clinic provides only primary treatments. You are not able to fulfill the entire demands.

P6: He is right. The thing you want to know is what kind of treatment or facilities other clinics are providing? For modern treatment community clinic is better place. For an example there is no nurse or doctor for delivery. So if a woman comes here and she is in a serious condition then it will be late for her to send her in Gouripur hospital. All these accommodation is absent here. Here we come for just fever, cold this type of problems. I am really pleased talking to all of you. You know the things, understand the issues and you can explain it very clearly. We have some bad experiences where people could not help us providing adequate information. The way you have made us understand about various issues, it proves that you are educated people. This is the reason we have come here. Now we have to leave. We don’t have enough time. Now if you could help us with some other information about the use of medicines, specially antibiotic. Mainly, we want to know about the use of antibiotic but if you could tell us their experiences of using other medicines. Do they have knowledge about it? I think we can get few more information from here.

M: Yes, I wanted to know about it. I would like to know about the medicines that are providing from this community clinic. Do you have idea about different kind of medicines? Do you know what types of medicines this clinic has?

P6: Here?

M: Yes, here.

P6: Here they provide antibiotic syrup for children, for the adult or old who comes with cough for them usually medicines for gastric or some other tablets.

M: What else will they suggest except tablet and syrup?

P6: they will suggest few more things like saline, medicine for blood pressure, acidity, cold- fever and sometimes they check the diabetic also.

P6: They give an ointment for pain. I live here, just beside this clinic. So if I feel any kind of illness I can easily come here for any kind of health services.

M: You are right.

P6: Beside this, I want to mention some other important things.

M: Okay we will give you another chance so that you can give your opinions to us.

P6: One thing is still remaining. Most important thing is they give vaccine to the children. They arrange vaccination program then provide vitamins for children. They provide vaccine for women. They give them a chart where they mention the dates. Like my grandchild has vaccination date on this 26^th^ date.

M: so the important thing is, can you separate these medicines? Do you have any idea which one is antibiotic and which one is other kind of medicines?

P6: Specially, those who are suffering from different kind of sever diseases for long period of time, they must have idea about medicines.

M: Can you separate the antibiotic and non- antibiotic?

P4: We have little bit idea about it.

M: Now, I want to know about the people living in this community. Do they understand it? Those who are talking here all of you are the supporter or member of this organization. But those who are general people living in this community, do they have any idea about the antibiotic and non-antibiotic? Are they able to separate it?

P3-P4-P5-P6: No, they can’t do it.

M: Okay. How do they recognize these medicines? Do they use any name? Suppose, you know this one is antibiotic. Do other people know it in an identical name?

P6: General people will recognize only medicine that is given for gastric problems.

M: Okay

P6: General people will recognize acidity tablet by seeing the mg like 20, 40, 12 etc. They can recognize the syrup and tablet for fever. They can separate the liquid and tablets. But there are a lot of group of gastric tablets. There are ranitidine, omeprazole. This omeprazole also made by hundreds of Pharmaceuticals Company.

M: So bottom line is, they can recognize the medicines but cannot separate the antibiotic and non-antibiotic. I have got it that you have little bit idea about antibiotic. At least u have known or heard about it. Do they have any idea about antibiotic? Do they have ever heard about it?

P6: Usually when patients come to doctors like paramedical doctor that time doctor will tell them about the medicine. All of us we 8-10 people will sit in a place and noticed, doctor will say u are suffering from fever so I prescribed you this 250mg tablet. Or you have infections so, I prescribed you for cyprocin which is 500mg or 800mg. our village doctors tell them this one is antibiotic.

M: Now you tell me your ideas about antibiotic. What do you know about it? Some of you are not participating in our discussion.

P4: he has a good position (pointing someone) so he must tell.

M: Not like that. You also have ideas. So you tell me your idea about antibiotic.

P4: Most of the time doctors prescribe this medicine. So we know it.

P5: So far I know about antibiotic is, suppose today I am suffering from headache. Initially I will take paracetamol. If it doesn’t work out then doctor will suggests us for antibiotic. Antibiotic is for the crucial time.

M: Do you think all the medicines those have more power are antibiotics? So, you uncle, what do you know about antibiotic?

P7: Antibiotic is a powerful medicine. Suppose, someone had undergone through operation, so to cure the wound doctor will prescribe antibiotic. This can be cefixim, ceftriaxone 0.1 mg or 0.4 mg. If the patient is unable to take it orally then the medicine will be pushed by injection. There are different types of antibiotic capsules. Initially doctors suggest less powerful antibiotic. If the disease like fever does not cure with that then they will suggest for some test to find out the nature of the disease. Once they get to know about the germs then they find out what type of antibiotics will be able to cure it. The patient will not be cured till the germs has fully distorted.

M: So antibiotic is that medicine which will be used to kill any germs completely. It depends on the severity of the infections. Certain power is adequate to destroy the germs. It can be different according to the nature of the germs. I have understood, all of you are familiar with the antibiotic based on it’s power. People use various antibiotic based on their power. I can share my own knowledge about antibiotic with you.

P5: One antibiotic is different from another.

M: Yes, probably you are saying the same thing. The thing is every germ has its identical name. You might have heard about virus and bacteria and they are different from one another. Now the important fact is this antibiotic only works to destroy bacteria. Not for virus. Have you got it? Antibiotic works to destroy bacteria or in other words antibiotic fights against bacteria. Usually we use the antibiotic to destroy all kind of bacteria or in other words for the treatment of those diseases caused by bacteria. So now we will jump to our next topic. Before we start I want to clear one thing, whatever I have understood that the common people of this community do not know about antibiotic. Now you tell me when any patient come to CC for treatment and CC gives any medicine or antibiotic, that time does he tell about the medicine to the patient? Does he clear about the use of that medicine to patient?

P5-P6: Yes, he tells it very clearly.

M: Can anybody describe it to me how CC do so? Suppose, CC is suggesting a particular medicine, so how does he describe? What important issues will he mention?

P5: Suppose, he gave 1 antibiotic. That time he will mention the cause of this antibiotic, like this one is for fever. Then he will tell the times like at morning, noon or at night this one will have to take. There are some capsules that have to take before meal, some have to take after meal. All these things he will tell.

M: does he mention about the particular period?

P5: Yes, usually 2 days or 5 days or 7 days. He prescribes it according to the nature of disease and power of medicine.

M: So you mean that doctor will prescribe the medicines according to the severity of the disease. It that case does anybody said you to complete the full course?

P5: Yes

M: What did they say?

P6: They initially give us medicine for 2 days or 3 days. The whole course might take 7 days or 10 days. But doctors tell us to take medicines for 3 days, then they ask us to visit their after 3 days. They don’t really give us full course medicine at a time.

M: do you mean they don’t give you full course medicine?

P6: Yes, you might take full course medicines. But after 3 days or 4 days or after 5 days you will have to check the doctor again. You will have to take it for 15 days. But doctor will give you 5 day’s medicines or 4 day’s medicines. Like this you will have to take it time to time.

M: I think you are trying to say it is a 15 days long course.

P6: Might be. Doctor might forecast that it will take 15 days to cure the disease. But they are not able to give the complete course of medicines at a time.

M: But the doctor suggests them to collect the medicines time to time. One more thing I want to know that, do they ever say the name of medicines. Precisely, do they ever mention that they are giving antibiotic? Do they clear it? Can you help me out telling this? And if they mention also how do they do? How do they describe? Do they mention that if the patient does not complete the full course then those left over medicine must not be share with anybody else?

P6: Not to use those medicine further.

M: Yes

P6: the thing is, the villagers they are not educated and concern enough. So what they do is, suppose someone is suffering from fever. So they will wait for 2 or 3 days. If in this period the fever got cure then they will not visit doctor. Or they will take the leftover medicines. Or they will not complete the course. It is not right to keep the leftover medicines.

M: Does the doctor warn about it?

P6: Of course they do.

M: Do you agree with him?

P4-P5-P6: Yes, we do.

M: You said that doctor warns you to not to take the leftover medicines. But what does he warn actually? You can take your leftover medicines, or you cannot take your leftover medicines, or you cannot give others your leftover medicines. Thing can be like this. So what exactly does the doctor warn about?

P6: No, he does not say like this. It is the responsibility of a doctor to tell this. Doctors only can tell suppose if I am cured now then am I able to share it with some other patient of not.

M: In that case this thing I am trying to know from you. Suppose, doctor gave him 3 day’s medicines (pointing somebody), but he got cure in 2 days. Now what does he do with that leftover medicine or that one day medicine? Does doctor say him not to give it to anybody else?

P6: Of course doctor does.

M: So, doctor tells. Now, as he said that if he has got the leftover medicines that time what he actually do with it. Does he come to CFC and tell about it to doctor? Does they ask to doctor before they share it with any neighbor or anyone else?

P4-P5-P6: No. No one do so.

M: Does doctor say not to share the medicines with anybody else?

P6: No, doctor does not say it.

M: So doctor does not say not to share it with anybody else. But he warn you not to use it further.

P6: Yes

M: Okay you have given me some important information. So it can happen that in case of scarcity or in case of insufficient storage of medicines if doctor ask you to come after 2 days and when you come and see there is not enough medicine in stock. What does doctor say under this circumstance?

P6: He will say we don’t have enough medicines to provide you. So it will come after 15 days. You can collect it after 15 days.

M: So he says so. Does he ever ask you to collect it from somewhere else?

P6: Not really. But once he said to me if I cannot wait for the medicines to come then he will write down the names and I can buy it from somewhere else.

M: What did he mean about “somewhere else”?

P6: Pharmacy

M: Is it here?

P6: Yes

P5: He doesn’t mention any particular pharmacy.

P6: He does not mention any pharmacy name. He just write down the medicine’s name and ask to buy those from pharmacy.

M: Where do they go in that case? Do they go to this pharmacy only?

P6: Yes, because it won’t take much time to go there.

M: Leave the pharmacy. Tell something else.

M: Okay. Now I would like to know, where do you get antibiotic from? Mention the place in your area. Can you show me?

P6: Here everywhere you can get it, At Gouripur you can get it. This one is also available at Mujibmarket. Sometime in a particular place you might not get all of the medicines. That time you need to look in some other places. In Gouripur it is available most of the time, at low price than Dhaka.

M: Now you tell me, if CFC let you know that there they have enough stock of medicines, and during that time doctor ask you to come after 2 days that time where do the people go? Do they collect it from here or will they go to somewhere else?

P6: Usually the poor people will come here. They will collect medicines time to time. They do not go anywhere else. There is another reason to come here. The quality of medicine is good here than the other places. It’s even good than that hospital. So people tend to come here even if they got to wait for 2-3 days.

M: So you have mentioned Gouripur. Do the solvent families come here or do they go somewhere else?

P6: Most of the time they also think like thay can spend money for the medicines. But they will not find good quality medicine out there. So normally everyone rely on this clinic.

M: If the medicines are not available specially during the period someone has started a course, what they do then? Will they move somewhere else?

P6: They might go. If the necessity is like that then they will go. That time they might go to Gouripur Health sheba kendro. Like, on today afternoon even I will go. Some people will request the doctor to write down the medicine’s name so that they can buy it when necessary. Specially after 2PM there is less possibility of getting medicines there, so they will go to hospital to collect it.

M: Does it ever happen here that antibiotic is completely out of stock?

P6: Sometimes doctor announces through mike that medicines have been reached or available.

M: Okay. What kind of medicines will fully be out of stock? IS there any particular medicines? Do they ever announce to not to come here because of the scarcity of any particular medicines?

P6: They will only inform us when medicines come. Once anybody come here then only he will know that particular medicine is out of stock. Other than this there will be no announcement.

M: Does he tell about any particular medicines?

P6: Not really

M: I would like to know, how is the availability of antibiotic at your area?

M: Easy to get it.

M: Easy

P6: Yes

M: Where is it available?

P6: At this market. As I mentioned earlier about which one is not available in Dhaka that one also available here.

M: Do you write any prescription for the patient who goes to buy antibiotic?

P6: Prescription in necessary.

M: Do they sell medicines without checking prescription? Or do they sell?

P6: those who are pretty familiar with the medicines like he needs Amoxicillin 500, they will ask the medicine directly.

M: Why so?

P6: Because this one is known to all. So whenever someone suffers from fever or cold they will simply go and buy.

M: Just now you have said they are taking it in a regular basis. Do they take it based on prescription or do they take it from their choice?

P6: Often he takes it. It works for ulcer and gastric also. For example, it will work for any pain. I used to go once in a while and buy one file of 5 days course. Then the pain like knee or back pain will be cured. I have talked to doctor also. He asked me to go for a surgery. Or else he advised me to take it for the relief of pain. There are quite a number of people like me.

M: Is it same for everyone?

P6: I cannot say like that. Specially those don’t suffer like me. It’s been 20-25 years I am suffering from ulcer.

M: You have said that, people usually buy medicines like this. Do they buy it without prescription?

P6: they have to get it showing prescription.

P5: Here, no allopathic doctors will suggest any medicines without prescription. Initially, doctors will ask about the problem and on that basis they will prescribe any cure or medicine. Sometimes, who are permanent patients and those who already have prescriptions from any specialist, our doctors will go through that and suggests any medicine. Or this may happen, they have prescription at home and they need some medicines those they know the names also, that time they can buy it easily.

M: So the thing is patient has the prescription. And he knows the names, so if he doesn’t show it also he can get medicines.

P6: Yes, because doctor has prescribe it. Or he knows this medicines is his cure. It happened like this.

M: Is there any particular reference? Like any particular medicine.

P5: It depends. There is no particular medicine like this.

M: Now I would like to know, you r visiting CHC. Someone comes with their illness and CHC checks it and after that CHC suggests none of medicine. Is it possible? What do people do if such thing happens? One patient goes there because of illness or because of not feeling well. But once he checked you he said you don’t need any medicine for this. You need proper rest and tell the usual routine. How do people react when they don’t get suggestion of medicines? How do they think? What do they do?

P5: Most of the people don’t want to take medicines right. If anyone have sound health he won’t ask for any medicines. If thing is that, without medicines he can be cured, it will be best suggestion for him.

M: Is it same for everyone?

P5: Those who are health conscious will definitely obey this.

M: If I mention the poor or those who don’t have proper knowledge, in a word, everyone of this community. Will they accept it?

P5: I have seen most of the people accept it. There is no reason to not to accept actually. Someone suggest to take liquid to cure the diseases.

M: We are almost finish today just few more question left yet. I would like to know, do people obey, when CHC advises anything? He can write down a prescription or he may not. Similarly, he can suggest any medicine or not. Sometime he will give some advice orally. Under these circumstances do the people obey his advice?

P5-P6: We listen to him.

M: Okay you do. Does everyone do so?

M: Can you give us clear example of what do they say or what do you listen?

P6: For an example, When a customer goes to pharmacy and tell the problem then they may advise him not to take anything sour, or they advise him to take hot vapor. Like this they will advise people.

M: Tell us about the medicines. What do they say about medicines?

P6: What?

M: About medicines. Do you listen to the doctors when they ask you to take medicines according to a schedule? Do you take it accordingly?

P6: Everyone will follow the doctor’s advice. There is no one in the world who doesn’t listen to doctor. Even if a worst person asked by the doctor to stop any habit, that person will stop it.

M: this this we are trying to know again and again. Once we get this answer we have no more questions to you. Does doctor tell all this things here or in upazila level? What do they exactly say about the antibiotic?

P6: Actually, doctors will not always tell that he is prescribing medicines. He will do it as a step of treatment. He will write it for the patient. Sometimes those who sale medicines at pharmacy they will clear it. Or sometimes patient will ask about the medicines. So they will clear that this one is antibiotic or paracetamol or this medicines works for this. Is it possible to ask all these things to doctor? If you pay his fee 500 taka also he will not tell it. He will only tell I have written it so you go n take it.

M: For the last time we want to know, what do you do with the leftover medicines? Does doctor advise anything what to do with this? Suppose when you bought 10 medicines, and u got cure when u have 5 medicines left.

P6: Suppose I am suffering from fever, and I have cured also. I have some leftover medicines; in that case I will not take it. Medicines expiry date might be 4-5 years. We will keep it at home. For normal fever no one will go to doctors. For cold and fever people will take Napa. So that time they will look for Napa at home. It happens for children also. Everyone knows for children half Napa is enough. We don’t have any illiterate here. So everyone knows the adequate dose for the patients based on age. So they will do like this.

M: What do they do in case of antibiotic? Do they do the same as Napa?

P6: Customer will tell it. Like for any wound or for someone else at home they need antibiotic.

M: When you have some antibiotic left at home as you said just now as Napa, what do you do with that antibiotic?

P6: Doctor has to say about it. If doctor does not say about the use of antibiotic then how do people know about it? They are poor uneducated people. They are really simple common people. They don’t know about antibiotic.

M: Actually, it is not my question.

M: Do they keep leftover antibiotic or will they give it to someone else?

P6: Doctors will advise antibiotic for children. Though at first stage they will not advise antibiotic for children but later on day do that in severe cases. They will initially prescribe Napa or Napa extra. Are those antibiotics? These will be the 1^st^ suggestions. Children’s antibiotic is that one, which you have to mix with the water. It will be given for 7 days. The thing is they ask us to not to use the rest of it after 7 days. They will not prescribe antibiotic capsule for children. This one is for adult person.

P5: Actually the thing is, doctors prescribe the antibiotic in a limited budget. Suppose, for gastric, they will advise to take a tablet before meal at morning. But antibiotic is not like this. In fever first people will take paracetamol. It may be napa or napa extra. They will take napa only if they suffer from fever. Doctors will tell that if you get fever only take this medicines.

M: I will ask 2 questions and expect for direct answer from you. I have understood all of you. What is the use of antibiotic? Suppose some people have antibiotic left. This might happen, doctor asked him to take for 5 days. He took for 3 days then went somewhere else and forget the medicines at home. What do they do with these extra medicines? Do they share it with the person suffering from same diseases?

P5: No, we don’t share our antibiotic with anyone else. We don’t use the leftover medicines.

M: Does every one of the area do the same?

P4-P5-P6: Yes, definitely.

M: Okay my last question, do you know anything about antibiotic resistance? Have you ever heard the term?

P4-P5: No, we have not.

M: Does other people heard about it?

P6: No, they don’t.

M: Okay, we will draw a finishing line here. But before that I will tell you about antibiotic resistance. We have said we take antibiotic to fight bacteria right. So doctors will suggest us to take for 7 days or 10 days. Now somebody took it for 3 days and leave it because he is feeling better now. Or doctor asks him to visit after 2 days and that time medicines were not available.

M: We are talking about the antibiotic and it’s course. Suppose you have not finished the full course. And you are doing it repeatedly. Every time you take medicines for 3 days and feel good then you leave the 7 days course and it is harmful. Do you know about it? Does anyone say it?

P5: Yes, one doctor said. He is my uncle and assistant professor of neurosurgery at Dhaka medical college.

P5: This is the reason we know it. We were asking about this thing actually.

P5: Uncle said, suppose you have typhoid. You are given a 7 days full course antibiotic. You have taken it for 3 days and got cured. So you didn’t complete the course. Further if you suffer from same disease, then not in 3 days if you take 14 days antibiotic also you will not cure. That time you will have to start the course from the very beginning.

M: Now you have got it.

M: Have you heard one thing? He has given an example right now.

M: Have you heard it?

P6: Yes. Doctors say it. They suggest the medicines for 5-7 days. Usually people will take it for 7 days. But mean time they will get cure. Even after 3 days or 7 days the same person will suffer from same disease. That time doctors will ask about the previous 7 days course. But patient will show the excuse of getting cure in 3 days. Then doctor will give the full course from the beginning.

M: What do they do with the leftover medicines from 1^st^ course?

P6: They will throw it out. If they keep it I will have no use. Not everyone understands the value of antibiotic. Not everyone knows the actions- reactions of antibiotic. Common people are unaware about the use of antibiotic. They will buy medicines for fever.

M: Which one will people buy?

P6: Napa or any other paracetamol for fever. But in case of serious issues they will visit doctors.

M: Now I would like to know from you, you have said your uncle talks about antibiotic resistance. Do they say it to you? Does CHC tell about it?

M: Do they? (Pointing to someone)

M: Do they tell it?

P6: Yes, they do.

M: Does your CHC tell?

P6: I don’t come here for this thing.

M: have you heard it from CHC?

P4: Yes, Kawsar Ahmed said.

M: Does he?

P6: Yes, Kowsar sir does. The course is for 7 days, n full course have to be completed. If you don’t do so then you will have to take it from the very beginning.

M: Will they tell like this?

P6: Yes, here the most senior is Kawsar Ahmed. He will give the correct advice to any patient. He will warn about the repetition of medicine of the course has not completed.

M: If you want to sign it in then you can do it. We will not stay for long here. We know that whoever working at CHC or upazila Health complex, are doing very good. Because of their cordial effort we have reached here. We are here only because of their performances. But we have to assure the people. They might have limitations. We haven’t settled any training program for CHC members yet. This might happen we will work here to make people aware and conscious. But the topic will be “Antibiotic and its Use.” Or if you can relate some other issues with it those we can go for. Those might be the use of medicines, or to make the people aware about the use of medicines. We will work on it also with your help. We will come again someday to talk to you. Actually we want to let people know about the use of medicines. We have CHC and they are working really hard. But we do have responsibilities too. So we will come for this. It our pleasure that you have made your time and attend here in this hot summer. We have a CC book, where top 5 clinics have been enlisted in the entire area. And these 5 clinics are working hard to support you. You will be glad to know that you clinic is one of them. So we are really happy to come here, and we have talked to you. If we work together and help our government then definitely our dream will come true. Does anyone want to say anything for last?

P6: What else can we say? You have come here and show us some hope to work for us. We will be able to know a lot of things from you. We will have enough medicines for more advances treatment at our community clinic. We hope that this community clinic will be more advanced clinic in the near future. Thank you.

M: Yes, our government doesn’t have sufficient medicines in stock. That’s the reason we report about the demand for the medicines time to time. If you help us by providing some information regarding this, then it will be easy for us to know.

M: Again I am asking to you if you have anything to know you can ask.

P5-P6: We have no questions. Thank you to all of you that you have come here for our sake.

M: I have noticed that you have less support or effort because we haven’t informed you that we are coming here. If would have known it from earlier then we could get more information from you.

M: Okay thank you every one for your effort. Thank you again.
