# Supplementary material for: A process for developing a sustainable and scalable approach to community engagement: community dialogue approach for addressing the drivers of antibiotic resistance in Bangladesh
Source: BMC Public Health. 2020 Jun 17;20:950. doi: 10.1186/s12889-020-09033-5 (PMC7302129; doi:10.1186/s12889-020-09033-5)
Supplement: Supplementary file 7 — Additional file 7. CSG Members Male (2). Transcript of focus group discussion with male members of the community support group, region 2. [file 12889_2020_9033_MOESM7_ESM.docx]

| **Study Name:** **Community Dialogue for preventing and controlling antibiotic resistance in Bangladesh: Case for Support** | **Interview ID: CC2 Male FGD** |
| --- | --- |
|  | **Date of Interview:**  **11/04/2017** |

**Potential Intervention:**

M = Moderator

P = Participant

P1: Huzur (Religious Leader)

P2: Teacher

P3: Businessman

P4: Ward Member (Community Group)

P5: Social Leader

P6: Businessman

P7: Landlord

I : I would like to understand the administrative breakdown of this area?

P1 : At first Upazila

P4 : After Upazila then Powroshobha

P2 : After powroshobha then Model union

P3: After model union then Ward

P5 : After ward then Village

P6 :After village then Para.

P7 : Each union has 9 wards, and each ward is divided into villages depending on the number of voters.

P1: 1 ward could contain 1 village or more.

M: Please can you tell me about any meetings that are currently held within the community to discuss health issues?

P1: Generally meetings are currently held within the community such as social issues like marriage.

P2: Meetings are held on sometimes discuss about natural calamities

P4: Yes, currently meetings are held on while preparing for a game such as football tournament in our locality.

P5: Meetings are generally held on any fights or clashes between villages and to meet up the conflict between them.

P6: Meetings are held on with influential people of our community such as discuss about prayers during Eid. I will be held on two times in the year (in 2 festival).

P7: Suppose setting up a tube well in an area meeting can be organize

P2: Discussing about roads meeting can be arranged.

M: Who initiates the meetings?

P1: CHCP initiates it

P3: CHCP organize these meetings

P5: CHCP and community group members initiates the meetings.

M: Who is responsible for organizing them?

P6: CHCP is responsible for organizing this meetings.

P3: CHCP

M: Who is involved in mobilizing participants?

P4: In case of Eid prayers, the people (men) get together for prayers. The time and venue are announced on the mikes present at the mosques.

P6: In case of a natural calamity, a small community of people, especially the influential people are informed, and then the others are gathered together by announcing the time and venue on the microphone present at the mosques.

P2: In addition, in case of any social event, people are gathered together through influential people by sending letters, spreading a word through other people, by spreading a word whenever anyone comes across on the streets, by giving calls.

P3: In case of any political event, the initiation in taken by the Union Parishad Chairman and Union Parishad Member, and the people are gathered with the help of influential people of the community.

M: Who usually participates in these types of meetings?

P1: Only the men of the village gather together when it’s for Eid prayers

P2: Only the women belonging to the community group and community support group attend meetings related to natural calamities, or issues such as getting a new tube well.

M: Are there separate meetings for males and females?

P2: When it comes to Eid Prayers, due to social restrictions, women don’t attend the prayers with the men

P3: In case of other meetings related to natural calamities, or issues such as getting a new tube well, only the women belonging to the community group and community support group attend these.

M: How often do they occur?

P1: Could last for an hour

P4: It occurs an hour and a half

P6: It depend on the discussions

M: Where are they held?

P7: Anywhere with adequate space the meetings can be held.

P2: Sometimes in Mosque

P6: In school ground the meetings can be held

P5: In schools playground can be held

P1: Meetings can be held any adequate space

M: How long do they last?

P7: Also depending on the people needed to be gathered.

M: What sort of challenges do you think prevents people from participating in these types of meetings?

P1: In case of Eid prayers, women don’t attend as the society doesn’t permit it

P2: In case of other meetings, women, apart from those belonging to the CG and CSG seldom attend, as they are not called, and are busy with their responsibilities.

M: I would like to know about the ways that people in this area currently learn about health issues? Who delivers health information?

P1: There have been health education sessions which have been conducted through Government Organizations (GOs) and NGOs

P4: Community Group and Community Support Group members delivers health information

P2: Shastho Shebika (Community Health Workers) from BRAC delivers health information

M: What is the format in which the information is provided?

P3: When the health education had been conveyed through the GOs and the NGOs, the people with influence were asked to gather other people at the field (playground), and the people responsible for giving the sessions delivered the information through lectures and flip-books.

P6: CHCP gathers the member of the CG and CSG and disseminates information which has been delivered from the Upazila Health Complex. The CG and CSG members then disseminate it after going home and informing people in their surroundings. The word spreads in this method.

M: What do you think people trust both in terms of the people delivering the information?

P3: Yes people trust the members of the CG and the CSG members

P5: People trust the Influential people

P7: Trust in People from Government and Non-government organizations

M: Do people prefer photos or drawings?

P1: Whatever’s delivered people can prefer

P2: Whatever deliver we can prefer

P4: We prefer whatever they delivered

M: Can you tell me if there are already people who work as facilitators or volunteers?

P1: No, there are not any volunteers

P2: No existing volunteers

P3: There aren’t any facilitators / volunteers

P4: No

M: What are criteria if we like to recruit any volunteers in this area?

P1: The main criteria should be educated

P4: Should be patience

P5: Able to work

P6: Able to speak well

P7: Should have the will to work

P2: Should be well mannered.

P3: Should be voluntary power

P4: Could be males and females

M: Who selects these volunteers?

P2: It would be preferable to recruit volunteers by deciding with the Community Group and Community Support Group members

P6: It would be preferable to recruit volunteers according to the community clinic, then the volunteers could be allocated to areas according to the villages later, through discussion.

P5: The volunteers could also be taken according to the number of voters.

M: Who supervises the volunteers?

P7: It would be best if the decision regarding supervision came from higher authorities. This is because in the absence of a decision from higher authorities, no method of supervision will function.

P4: Yes, it would be better if the decision came from higher authority.

P6: It would be better if the decision came from Community Group members

P1: It would be better if the decision came from Community Group members and Support Group members

P2: It would be best if it will manage through community clinic members.

P3: There will be maintain a routine that who will visit which area and how long they will stay there. The supervisor (community member) will check the routine.

M: What do you think motivates volunteers to work?

P1: Making sure that the volunteers have an opportunity to work i.e. as per their convenience.

P4: Hope of having a government job

M: What kind of incentives for their work might be required?

P2: I think a small amount will have given to them

P6: May be money

P4: I generally think that it may be conveyance

P1: Most importantly, if the person doesn’t have the will to work, no form of incentive will be helpful.

P7: Rest can be decided by the authorities (research team, whatever seems feasible)

M: How long they work on a week?

P1: 2 to 3 hours per week can be conducted

P5: Yes 2 to 3 hours per week may be possible for the volunteers

P6: Yes they will provide 2 or 3 hours per week.

M: Who belong to the community group and community support groups?

P3: Generally 17 members in the Community Group

P6: 17 members in each of the 3 Community Support Groups

P4 : There has to be a freedom fighter

P5 : Has to be a teacher

P2: Has to be a Disabled person

P7: Has to be widow

P1: There has to be a religious leader

P2: has to be a farmer

P6: has to be a Social worker

M: Who is responsible for selecting them?

P1: CHCP selects the community Group members

P3: Union Parishad member selects them

P4: The landlord selects them

P7: There could be a person from the Upazila Health Complex during the selection process

P2: CHCP selects them

P6: Landlord selects them

M: What are the regular activities of the group?

P 1: Informing people about the existence of the community clinic (CC)

P4: Motivating people to avail services at the CC, especially the pregnant women

P6: Motivating the people to follow the advice of the CHCP

C7: Following up on whether the people have gone for further check-ups, if advised

C3: Checking if the medicines have been sent from the government. On arrival of the medicines, the CHCP unpacks the boxes in the presence of the CG and CSG members.

P2: In case some medicines are inadequate, the members of the CG and CSG discuss the next step, and write an application to the Upazilla Health Complex stating the issue if it can’t be solved through discussions.

P6: Checking if the CHCP is providing services and medicines to the people of the community

P5: Advising pregnant women to be careful during the rainy season and have their supplements

P1: Advising pregnant women to get vaccinated

P2: Asking the pregnant whether they have thought of financial arrangements for delivery or Cesarean section, if required.

P3: Advising the to-be parents to start having savings for the future

P4: Checking if the medicines have been sent from the government. On arrival of the medicines the CHCP unpacks the boxes in the presence of the CG and CSG members.

P5: In case some medicines are inadequate, the members of the CG and CSG discuss the next step, and write an application to the Upazilla Health Complex stating the issue if it can’t be solved through discussions.

P6: Checking if the CHCP is providing services and medicines to the people of the community

M: Are some members particularly active?

P5: Everyone’s pretty much active. Some may not be able to attend meetings due to personal reasons.

P6: Apart from that, everyone pretty much does whatever’s expected from them.

M: What happens if a member of the community group or support group does not want to participate anymore?

P4: Generally a person can back out from the group, and the remaining members get together to discuss the recruitment of another person in his/her place

P6: Generally Members get together to discuss the recruitment of another person in his/her place

P7: Members get together to discuss the recruitment of another person

P1: If anyone wants to get out from the group he or she can do that of their own decision.

P2: Many people have interested to join this group. So replacement is not a problem.

M: Who supervises and monitors the work of the community group and support group?

P1: The members are called for monthly meetings by the CHCP

P2: CHCP monitors community group members

P3: Community group monitors community support group members.

M: What enables the functionality of the CSGs?

P1: The members of the CG and the CSG come together once every month to discuss about issues arising at the clinic

P3: The opportunity to work as per the convenience of the members

P4: The will of the members to work

P5: They motivated to work for the welfare of the community

P6: The feeling of responsibility to look after the less privileged, and seeing to it that they receive proper health care

P7: According to the landlord- the land has been given for the Community Clinic, however, it’ll be unworthy if the services and medicines are not provided accordingly.

P2: The feeling of responsibility of whether the government has given adequate medicines.

M: What makes it difficult for them to function?

P6: Due to lack of facilities from the government for the community, it becomes difficult for them to motivate people such as Inadequacy of drugs.

P2: Lack of proper roads, especially for the pregnant women.

P3: Lack of an on-call transport service, or any form of transport to bring the women to the Community Clinic

P4 : Unavailability of a doctor, at least a female doctor, who could attend the clinic at least two to three times a week to check on the patients, especially pregnant women.

P5: Lack of services for neonates and Lack of electricity

P1: Lack of foods and money

P7: The breaking of land around the Community Clinic

M: Who solve those problems?

P6: Lack of medicines are sometimes solved by the CG and CSG buying the medicines from the pharmacy, or giving the money to the person to buy some medicines, or advising the person to return the next week.

P2: The land has been breaking away, the roads around the CC has been damaged, so the members got it fixed.

P4: Problems related to the government are left as they are, such as lack of maintenance of the Community Clinic

P5: Dripping of the roof

P7: Lack of a doctor, at least once a week

M: Potential for CSGs to be involved in monitoring and supervising facilitators?

P1: If the volunteers were to be monitored and supervised, they could be chosen with the help of the CG and CSG members.

P2: There could be a meeting between the authorities (i.e. the research team), the volunteers and the CG and CSG members, where the volunteers would know that the CG and CSG members are responsible for supervising them.

P3: There could be a duty roster at the clinic with an attendance list for the volunteers, with the allocation of areas, and names of CG/CSG members who’d be responsible to supervise the corresponding volunteers.

P4: The CG and CSG members could follow up with the households to see if the volunteers have been working accordingly.
