# Supplementary material for: A process for developing a sustainable and scalable approach to community engagement: community dialogue approach for addressing the drivers of antibiotic resistance in Bangladesh
Source: BMC Public Health. 2020 Jun 17;20:950. doi: 10.1186/s12889-020-09033-5 (PMC7302129; doi:10.1186/s12889-020-09033-5)
Supplement: Supplementary file 8 — Additional file 8. CSG Members Male (3). Transcript of focus group discussion with male members of the community support group, region 3. [file 12889_2020_9033_MOESM8_ESM.docx]

| **Study Name:** **Community Dialogue for preventing and controlling antibiotic resistance in Bangladesh: Case for Support** | **Interview ID: CC3 Male FGD** |
| --- | --- |
|  | **Date of Interview:**  **17/04/2017** |

**Potential Intervention:**

M = Moderator

P = Participant

P1: Imam (Religious Leader)

P2: Student

P3: Ex-member

P4: Teacher

P5: Community support group member

P6: Landlord

P7: Member

P8: Health Worker

M : Our introduction session is over now. Here we have so many elderly respected person. I hope you will co-operate me by answering my question. so at first I would like to understand the administrative breakdown of this area?

P1 : At first Upazila, then union and then ward.

M: How many unions are here?

P3 : This Upazila is consisting of 16 unions. Every union consists of 9 wards.

M: How many wards in each union?

P3: Every union consists of 9 wards.

M: Is this according to the recent administrative breakdown?

P1: Not so recent. It is about 9 to 10 years when it was decided. Ward is consisting of 2 villages.

P4 : Each ward consists of 2/ 3 villages. It depends on the number of people living in the villages. Government has decided how it will be broken down.

M: What about the villages? How many clinics are in a ward?

P7: The rule is to have one community clinic in one ward. But we have one and people of ward no 1 and 5 come to our clinic because there are no clinic in their ward.

P8: People come from Moloy, Shayestanogor, Laksmipur, Tinchita, Dakkhola, Balikandi to this clinic for treatment because of the communication is good.

M: How many people come here for treatment?

P8: People of three wards come here for treatment. Around four thousand people.

M: Are there any other breakdown after village?

P4: No

P1: No

M: Are there anything called para or moholla?

P5: They recognize the smallest unit as “Bari”. They are maned by the surname of the head of the family.

M: How many families live in a Bari?

P1: There are 170 families in our village.

P6 : A small village is consisting of 17/18 Bari. We know each other in this village.

P7: Each Bari has 9 to 10 families (households).

M: Okay, I got your point. So the smallest unit is Bari but this is not included in the administrative breakdown process. Now please tell me how the people in this community how the people in this community get to know the health information? Please can you tell me about any meetings that are currently held within the community to discuss health issues?

P1: People get to know about the health issues (vaccination, family planning) from community clinic.

M: Okay… One source is community clinic. Then?

P8: Through Health Assistant and another is Family Welfare Assistant.

M: Are they both are related to this community clinic?

P8: Yes. When people go to the health complex for the treatment purpose, on that time they receive some health education from there.

P5: Another is TV media

P4: There are Tv media, internet.

M: Okay… I understood. But I wanted to know about some gatherings, meetings similar to the political meetings but where people come to learn about some health issues or discuss about it?

P5: In the Union Health Complex.

M: But I am talking about the general community people…

P3: Any meeting on any social issue is held in “Madrasa field”

M: How many people are gathered there?

P8: This is a gathering round 150 to 200 people. Sometimes the field becomes overcrowded.

M: What are the usual discussion agenda of the meeting?

P8: Any issue related to this village.

M: let’s come directly to the point. Are you familiar with the term Uthan Boithak?

P6: Yes, many sessions of Court yard meeting are held in our community.

P1: The Imam announces through the mike of the mosque about themeeting.

P8: How a pregnant women should be taken care, increasing the awareness are discussed there.

M: How a Court yard meeting conducted?

P6: They are held in the Bari.

M: Are they held regularly?

P8: Not regularly. Sometimes.

M: How many people usually attend a court yard meeting?

P8: 30 to 50 people.. Sometime it may reach up to 100.

M: Who attend the meeting? Are they arranged for females only?

P6: Male female both attend the meeting.

M: Who conduct the meetings?

P8: Health Assistant and another is Family Welfare Assistant.

P6: There are some other people who conduct court yard meetings.

P4: Meetings/ trainings by NGOs usually held on healthy lifestyle practices, family planning issues.

P3: The meetings of NGOs like UDDIPON only the members can attend the meeting

P5: Micro credit issue and few health related issues (Healthy lifestyle practices) are usually discussed in meeting of corresponding NGOs

M: Are there separate meetings for males and females?

P4: There is no gender preference

M: Who initiates the meetings?

P1: Upazila Health Complex, Community Clinic organize meetings(Health Assistant, Family Welfare Assistant)

P7: NGOs like BRAC, UDDIPON organizes meeting in their organization office.

P8: BRAC works on TB, Diabetes. They come here and train their health workers especially to the women.

M: How often do they occur?

P1: There is no specific day for meeting

P3: These meetings may organize in monthly

P4: Sometimes half yearly or even yearly the meeting occurs.

M: Where are they held?

P6: Meetings are usually held in Upazila Health Complex

P7: Sometimes in Community Clinic

P8: In NGO office

P3: NGOs have their health workers to conduct the sessions

M: What is the format in which the information is provided?

P3: Usually information are delivered through oral

P6: BRAC uses projector (Screen) and shows videos and pictures to deliver messages.

M: Do people prefer photos or drawings?

P1: It will be better if pictures, posters are shown while giving the health education.

P1: Anything

P4: We prefer whatever they delivered

M: okay… let’s go back to the point again. What do you think… who should be the facilitator of a courtyard meeting? Male or female?

P6: Male and female both.

P1: Nowadays we listen to the women more…girls are more educated than men. Men start to work at their early age.

M: What will help a person to be motivated to work as a volunteer? Is there some people who will gather people by their own and talk with them about health related issues?

P3: It could be done by government and nongovernment organization. Even a retired teacher can arrange a meeting. Everyone will listen to him and will give value to his/her opinion.

P7: If an important massage is delivered in the meeting, I am sure that people will take it seriously. But for that you have to choose an elderly person or respected person from the community like member and other retired person.

M: You said that courtyard meetings by other NGOs are held here. how long do they conducted..

P8: Half an hour or more. I t might be one hour too.

M: do you know the way of recruitment of the facilitator?

P6: Usually UP member recommend 10 to 20 people and they are trained and do their job.

P4: Usually the volunteers in the area are selected by the UP chairman and the members and others are selected by their own organizations.

P6: Generally People have no objection with the process.

P8: In case of NGO they provide training to some women for 3 to 4 days and thnen they start to work.

M: How their area of works is distributed?

P6: After training they are being instructed and their area of works is specified.

M: Now tell me that the people who are doing such works, how they become motivated to do this? What are the reason of being willing to do this work?

P1: Motivation comes with the work satisfactions. Mainly incentives can motivate them to work

M: What kind of incentives for their work might be required?

P1: Money can be given for food and conveyance but it will be better to give it as salary.

P3: May be money

P5: I generally think that it may be money

P8: Rest can be decided by the authorities

P3: actually it comes from your inside that you want to do something for the people , for the community.

M: Okay… Please tell me about the volunteers of your community. Can you please tell me about the total number of volunteer who are currently working on your community?

P4: No one..

M: None?

P4: We make people volunteer according to need and situation.

P7: I have no idea about volunteers. But it is not possible to cover all the area by the HA and FWA. There are 24 people working in 8centres. They might be the volunteers!

M: gentlemen… I want to explain my project in front of you and I am going to ask for some suggestions and advices from you [moderator explains the “community dialogue”]

M: so making this project happen we will need some volunteers who will voluntarily conduct the Uthan Boithak to aware the people about antibiotic resistance. I want you to give me some suggestions that how can

we convince people to a volunteer and work with us?

P7: Usually union parishad members and chairman select the volunteer. In that case I can help you out. Whenever you want, we will select few people to help you out. I am a member. I have a responsibility to work for my community people. I will make a list of people who will work as a volunteer and will give it to you.

P8: If you agree then it could be done.

M: If you make the list, what will be the criteria to select them according to you? Because we want to make volunteers according to your choice.

M: What are criteria if we like to recruit any volunteers in this area?

P1: The main criteria should be educated

P4: The facilitator should be healthy

P3: Have to knowledgeable

P8: Have to young/retired

P5: Able to work

P6: Able to speak well

P7: Have enough time to deliver the services

P3: Should have the capability to convince people

P7: In absence of chairmen member will make the list. He will give you the list of names.

P5: You will give us the responsibility to monitor the volunteers. We will take care of them.

M: Oh that’s great. Obviously you will make the list. But what about the characteristics that a volunteer should must have?

P1: A healthy, intelligent person who have enough knowledge.

P1: Retired person or teachers who have plenty of time to give.

P8: We have two freedom fighters here with us. I think people like them also can give you full time. Imam can also give you time.

P7: If you give someone the responsibility, he will surely co-operate with you and help you .

P4: This will only possible by an educated person

M: Do you have any gender preference? Male or female?

P4: Male volunteer for male participants and female volunteer for female participant.

P1: male and female volunteer both will be needed.

P8: Male female of all age groups can participate in the court yard meeting

P7: You will have to give them guidebooks

M: Yes of course, we will provide training to the volunteers and they will come from the community people so they will be needed some education related to it.

P7: If you work in our ward, I am ready to help you any time with manpower, labor.

M: Thank you… That is so kind of you. Now I would like to know about what will motivate a volunteer to work 2to 3 hours in a week? He or she will have to spend 2 to 3 hours in conducting court yard meeting.

P7: Actually even a child will not do a work if he gets any benefit of it. So we can’t expect someone to work for us without any benefit. No one will agree to spend his or her time in this project without benefit. I am a public representative. I have to work as volunteer. But the general people are not bound to. And they’re poor people.

M: Is there anything except money that can motivate people to work as a volunteer?

P4: There is no way to motivate people without money. They will work for you, give you their time, in return they can expect a minimum amount of money. After that if you give us the responsibility to monitor them that we can do as a volunteer.

P7: If you are not there to supervise them, just make a phone call. We will be there to monitor the volunteers.

M: So you are trying to say that the community group member and community support group members will monitor the volunteers?

P6: Yes, in your absence we can do that. We can also inform people to cone to tha meeting.

P7: If you tell us to gather 100 people in the meeting, we can arrange them and I am sure all will participate willingly.

P8: You just need to inform us timely.

M: Your support will be needed. To whom a volunteer should report about his or her work?

P8: we have already said that they can come to us and repost us.

P6: One more thing.. the person who are currently working will not be able to work for you.

P7: It will be better if you choose the young generation for this work.

M: Will they be willing to work as volunteer?

P1: Yes, School going young boys and girls. We are old enough now. We won’t have that energy to do this work.

M: Okay… lets come back to the point again.. Is it possible to monitor the volunteers if we link them up with community group and community support group?

P1: If you show us the way, we can make it happen.

M: I would like to know about the responsibilities of you all toward this community clinic and community people. What about the meeting? How often it held?

P1: Sometime.

P7: Let me tell you…. the CHCP and FWA inform us about the meeting, its time and agenda. We all try out best to be present at the meeting. Beside this, when the medicines arrive in the clinic, the CHCP unpack the medicines in front of any of the member of the group.

P6: Any issue related to community clinic are discussed in the meeting and we try our best to give solution of them.

M: would it be possible for you to monitor the volunteers through these meetings? Would it be feasible if they attend these meetings and report you?

P8: Of course possible.

P1: yes. We will verify if he or she facilitating the meetings properly or skipping.

M: This question should be asked earlier. I am sorry that I am asking this now. Who is responsible to choose community group and community support group members?

P5: there are one community group and two support group in this clinic.

P7: My uncle who was the former member of this union, he was the chair of this community group. He chose the other community group members with the help of CHCH and the people of the community.

P1: There has to be a social worker

P7: has to be a student

P8: has to be a freedom fighter

P1: Has to be a retired person

P6: Family welfare assistant

P6: There are some categories of members which were given by the upozila health complex such as land lord, teacher, widow, villagers, member etc.

P1: The UP member discussed with the other community people and selected the members of the CSG group

P3: But UP members have to follow criteria and category

P4: The members of CSG are selected by the member of the UP.

M: Do all the support group work together or they have got their work distributed?

P1: we are here to support the clinic. We don’t have that much vital work. Sometimes we do publicity for the clinic so that more people get to know about it and come here for treatment.

M: How much time can you spend for this clinic?

P7: As much as you want us to.

M: Not for us… for the people of community, for this clinic

P6: There is no fixed timing. When the need us we just reach here to help.

M: we want volunteers like you. You know that there is no personal benefit for you but still you are doing your work.

M: Are there anyone who monitors the activities of this CG and CSG group?

P1: There should be but there is no one.

P5: No one is responsible for monitoring and supervising the CSG and CG member. They are responsible for their own activity.

P6: No one is executing the process

P7: There should be some accountability to responsible someone.

M: If someone stays absent in the meetings for a long what will you do to him?

P6: People have their own work. We all live in the same community. If any member misses the meeting we call up for another member. But if he/she is frequently absent in the meetings than dismiss his membership.

P1: We never force anyone to stay or leave the group

P3: If someone is constantly absent in the regular activities or meetings the other members manage to divide the responsibility of that member within other members.

P5: Members get together to discuss the recruitment of another person

P8: But usually we don’t take this serious action.

M: we got to know that your groups are too active. How they are called so? In basis of what?

P4: The members of CSG sit together once every month to discuss about issues arising at the community clinic.

P5: we discuss the issues at the community clinic

P7: We are motivated to work for the prosperity of the community

P3: This is all about trust. People have faith on us.

P7: You said active…. Members of the groups live near to this community clinic. They all come to the clinic in different times and monitor the activity of CHCP.

P1: The CSG member deal with the problems arises in the CC and tries to resolve them

P3: We supervise the work of CHCP, Health Assistant and Family Welfare Assistant

P5: The also present at the time of unpacking the medicines when they arrive from the UHC

M: Would you tell me what type of problems can a volunteer face while working?

P1: My responsibility to call people for prayer. No one will create problem on it. In the same way the volunteer will also work for the betterment of the community people. So no one should have any problem with it.

P7: The volunteer will work smoothly. As a UP member I can ensure you that.

M: People might not co-operate with the volunteer.

P7: You need not to worry. People want to learn about the health issue. They will co-operate. There is a possibility that you won’t get 100% attendance but at least you will get 40% of them present in the meeting.

M: Another question for you all…. How many volunteers will be needed for one village?

P4: if there are 18 Bari in a village and each volunteer covers three Bari then 6 volunteer will be needed. How much time the will spend on meetings?

M: Three hours in a day one hour in three days of a week. It will completely depend on the volunteer.

M: I have said earlier that we need people who will work voluntarily. But you told me that no one will work without benefit. So if we want to give them an amount as the cost of transportation, food what will be the better way?

P7: intensive will be very much needed. No one will work for free of cost. But how you will give it to them is totally up to you.

P8: You will select volunteer from our villages. So the distance could be the walking distance.

M: So what can we do?

P8: Give it to them as a monthly incentive.

M: Would you please give me an idea about the amount? This time we are doing this project in a small scale. So we are not able to give a very handsome amount.

P5: you can give then 300 taka per week.1200 per month.

M: One last question that I have forgotten to ask. Did you ever face any difficulty to work for the community clinic?

P4: Never… everyone helped us, co-operate with us.

P7: we have never faced any challenges or barriers because they are working for wellbeing of the community people.

P3: We have never faced any difficulties

P7: No difficulties generally faced to function

M: Thank you so much for listening to us and giving us you valuable time.
