# Supplementary material for: A process for developing a sustainable and scalable approach to community engagement: community dialogue approach for addressing the drivers of antibiotic resistance in Bangladesh
Source: BMC Public Health. 2020 Jun 17;20:950. doi: 10.1186/s12889-020-09033-5 (PMC7302129; doi:10.1186/s12889-020-09033-5)
Supplement: Supplementary file 9 — Additional file 9. CSG Members Male (4). Transcript of focus group discussion with male members of the community support group, region 4. [file 12889_2020_9033_MOESM9_ESM.docx]

| **Study Name:** **Community Dialogue for preventing and controlling antibiotic resistance in Bangladesh: Case for Support** | **Interview ID:**  **CC4 Male FGD** |
| --- | --- |
|  | **Date of Interview:**  **04/052017** |

**Antibiotic Use:**

M = Moderator

P = Participant

P1: Landlord

P2: Member of the Ward

P3: Freedom fighter

P4: Young Member

P5: Student

P6: Religious Leader

P7: Poor

M: Could you please tell me, how many villages do this community clinic provides health services?

P1: 5 villages.

M: Including this community clinic, please tell me, where else do the people of this community receive health services? Do you have any pharmacy in your area?

P1: Yes.

M: Where else do you go to receive health services?

P2: We receive primary health care from the CC.

M: Do you have any paramedic in your area?

P2: No.

M: Homeopath?

P2: No.

M: Traditional healer?

P1: No.

M: So, only the pharmacy?

P3: Actually, it is not a pharmacy. It’s a grocery but they also sell medicines there.

M: So, you get medicines from the CC and the pharmacy. Like this two facilities, do you have any other places where you can get health services?

P2: No.

M: Do you have any medical representative who sometimes provides health services?

P4: No.

M: Okay. How far is the pharmacy from the community clinic?

P4: It’s just a grocery.

P5: It is nearby the CC. It’s in the bazaar.

M: Do you go elsewhere to seek health services?

P2: In case of a severe problem, we go to the Upazila Health Complex.

M: How far is the Upazila Health complex?

P4: It takes approximately 1 and a half hour from here (indicating the CC) to reach there.

M: Where else do you go?

P2: Gouripur.

M: What is it?

P4: Daudkandi Health Complex is situated at Gouripur.

P5: There are some private clinics also situated there.

P1: Private Hospitals are also there.

M: How far Gouripur from here?

P2: It takes an hour to go there.

M: Now, please tell me about all of these places. Why do you go to Upazila Health Complex, Pharmacy and Gouripur or, Daudkandi Health Complex?

P4: We visit the community clinic to seek primary health care. If someone suffers from fever, he or she goes to the CC. If the condition cannot be treated here, we go to the hospital. Women and children are given vaccines from the CC.

P3: General treatment has been provided from here (indicating the CC). In case of a critical condition, we go to the Upazila Health Complex or Daudkandi Health Complex. If the condition cannot be solved there, we go to Dhaka.

M: Who mostly visit these places to seek health care; children, pregnant women or old aged people?

P3: Mostly, old aged women.

P1: Pregnant women.

M: With which health conditions do they visit these places to seek health care?

P3: Mostly, the old aged women visit these places with the condition related to knee pain, back pain, difficulties in movement and walking.

P1: Knee pain, back pain, pain in legs. If they suffer from severe fever or severe cold, they visit the CC. If they condition is critical they have to go to the Upazila Health Complex.

P5: The medicines for skin disease provided from the CC are good. Medicines for skin disease and diarrhoea are very effective and we get cured of these medicines.

M: As you said, the pharmacy is nearby the CC and you get medicines from the CC. Then why do you go to the pharmacy?

P3: The CC closes every day in the evening. People have to go to the pharmacy after that to get medicines---mostly, at night when they face any health related problem.

P4: Some believe that since the medicines are provided from the Government, these might not be effective enough and if they buy the medicines from the pharmacy, it would work well. However, medicines are available at the CC and they can avail those anytime.

P5: The fact is that the doctor of this CC doesn’t have an MBBS degree and it’s a problem. People visit this CC for normal health issues. But I am not confident enough about the services of the CC and each type of medicines are not available as well. Besides the medicines are not effective enough which makes our life difficult.

M: Okay. Do you have any idea about different types of medicines?

P2: Somewhat.

M: Could you please give me some idea?

P2: ‘Napa’, ‘Diaclophane’; antibiotics like Amoxicillin etc.

M: As you mentioned about antibiotics, can you differentiate them from the other medicines-- which are the antibiotics and which are not?

P2: Yes, we can differentiate those.

M: How does it look like?

P2: Antibiotics are the capsules.

P4: Sometimes these are found in tablet forms.

M: How do you recognise antibiotics—I mean by name or by shape or by effectiveness---how?

P3: We recognise these by name.

P2: Such as ‘amoxicillin’, ‘cotrimoxazole’ or, ‘flucloxacillin’ etc.

P5: We can identify which one is antibiotic and which one is not. The doctor always mentions about antibiotics every time she prescribes them. We cannot mention the names of all antibiotics. Some people know about antibiotics while most of them have no idea—even some have no idea about the completion of the full course of antibiotics.

P4: Antibiotics are being prescribed for 5 days. Antibiotics cannot be prescribed initially. Normal medicines are prescribed to treat the condition initially. If the medicines do not work, then after 5 days antibiotics are prescribed to treat the disease. In that case, the doctor informs the patient that normal medicines were not working well and you have to take antibiotics.

M: As you told me about normal medicines and also about antibiotics, could you please tell me, when the doctor prescribes normal medicine and when antibiotics?

P1: At first the doctor provides normal medicines.

M: Why is that?

P1: Suppose, someone comes with body ache; the doctor will prescribe paracetamol, antacid, or, vitamin tablets etc. When these medicines do not work then the doctor prescribes antibiotics for 3 days.

P3: It is applicable for diseases that are caused by a virus.

P4: The fact is that when someone visits the CC with health issues, at first the doctor provides primary treatment with less powerful medicines. After 5 days, the patient is given the antibiotics when he or she visits again with the same condition. The antibiotics are prescribed for five days and also advised to complete the full course. For instance, if he or she stops taking antibiotics at 3 days before completing the course for 5 days, it will affect adversely. The antibiotics will not work and she has to start the course from the beginning.

M: As you said antibiotics have been given to the patients, could you please tell me, when antibiotics are required to treat the disease and when it is not required? Could please tell me about some of the diseases which require antibiotics?

P1: In case of severe fever, antibiotics are given to control the condition when normal medicines failed to do so.

P4: Antibiotics are also prescribed for a cough and cold. Primarily normal medicines are prescribed. When the sputum is accumulated in the chest antibiotics are prescribed.

P1: In the case of healing of the wounds or infections, antibiotics are provided too.

M: Could you please tell me, what are the different types of medicines available here?

P1: Capsule, tablet, syrup etc.

P4: There are some ointments available to treat allergic reaction on the skin.

M: As you said, what does the CHCP told you about antibiotics while prescribing them?

P1: Every time she advises to complete the full course of antibiotics. If the course is not completed, then it will not only harm the health but also the disease will not be treated.

P4: Sometimes, antibiotics are given to people to treat fever immediately. When they feel better they stop taking antibiotics or do not complete the full course. They think that they have cured completely.

P1: There are some old aged men and women who do not to return to the doctor when they feel the fever has disappeared. But the doctor (CHCP) advises to complete the full course of the medicine, otherwise, it will not work on.

P3: If someone forgets to take one medicine in time, he or, she will have to start the course from the beginning.

M: Who told you that?

P3: The physician or the CHCP told that.

M: When you go to the pharmacy do they provide advice regarding the medicines?

P2: Yes, they also advise the same thing like the CHCP advises.

P3: Suppose, if I go to a pharmacy with a prescription, they will advise me accordingly.

M: If you go to a pharmacy with a prescription, do they suggest you to complete the full course as mentioned in the prescription?

P5: Yes, they always say that.

P2: The instructions of the medicines are also mentioned in the prescription.

M: Do people share medicines? Suppose someone received medicines for five days. After three days he or she felt better and stopped taking medicines. At the same time, one of his or her family members or friends was also suffering from the similar condition and he or she had shared some of her medicines. So, do you think people share medicines with others?

P1: No, they do not share medicines with others.

M: What about antibiotics? Do they share antibiotics with others?

P3: No, they do not share antibiotics with others.

P4: Those who need antibiotics would visit the CC.

M: Do the care provider advise people about sharing medicines with others?

P6: We have an idea about this that if we share medicines with others, it will harm them. There could be doses for different age groups and if it is not maintained would affect their health. So, it is better not to share medicines with others.

M: So, do they (health care provider) ask you not to share medicines with others?

P6: Yes, the care provider of this CC always suggests us not to share antibiotics with others but most of the other care providers do not advice regarding sharing of medicines.

M: What about leftover medicines? As I said earlier, suppose, someone has given antibiotics for 5 days and after three days he stopped taking medicines. Do the care providers give instructions to the patients about leftover medicines?

P1: They always suggest us to complete the full course of the medicines.

P2: Mostly, people buy medicines for 2 or 3 days instead of buying the full course from the pharmacy.

P2: If people keep leftover medicines or antibiotics at their home they do not return or inform the CHCP about this. The CHCP is not been informed about this. That’s why she never told us anything about leftover medicines, and also the other care providers never asked or gave us advice regarding keeping leftover medicines at home.

P1: Actually, sometimes neighbours ask for the same antibiotics or medicines that have been prescribed to them and since the medicines were kept at home they share those with them.

P3: And the CHCP has no idea or was never informed about this too.

M: But, do the CHCP or other care providers’ advice people about leftover medicines?

P4: The CHCP always advises people to complete the full course of the medicines. She is not been informed whether the patient has completed the course or not---she has no idea whether people kept leftover medicines or not. They never bring back the leftover medicines to her.

M: Okay, now tell me about the doses of antibiotics and how the instructions are given to use them?

P4: At first the caregiver provides us normal medicines for 3 days. If the medicines do not work then after 3 days she provides antibiotics for 5 days to treat the condition and gives the advice to complete the full course.

M: How many antibiotics does the CHCP give a patient for 5 days?

P4: 10 antibiotics. 2 antibiotics each day—one in the morning and the other in the evening.

M: Does she instruct patients about the time interval between the doses of antibiotics?

P4: Yes, she does that. She always advises that and while giving medicines to the patients makes a mark on blisters of the medicines with a scissor which indicates when and how antibiotics should be taken.

P1: Sometimes, she mentions the instructions on a paper for the patients.

M: Does she provide the full course of antibiotics?

P1: Initially, she provides normal medicines for three days. When the medicines fail to treat the condition then she provides antibiotics for 5 days.

M: Does she provide antibiotics after 5 days?

P4: No, she does not provide medicines after 5 days. If she finds the condition is severing, she advises them to consult with a doctor or, to visit the Upazila Health Complex where the health problem will be examined and will be treated accordingly.

P7: If the patient is sick then will be treated initially with 3 days of medicines and if the problem cannot be solved with normal medicines then antibiotics are given for 5 days.

M: If the supply of the medicines is inadequate that is patients have visited the CC for medicines and they are given only 3 days of medicines instead of 5 days, what they are told to do?

P2: She says, “Take these medicines for now and come after 3 days when the medicine arrives.”

M: And if medicine doesn’t come after 3 days what would they do?

P3: Nothing.

P4: Then they have to start the course again from the beginning.

M: Do you have adequate supply of medicines at the CC?

P4: Yes, always.

M: What does she says when there are no medicines or the supply of medicines is inadequate?

P5: She advises to collect rest of the medicines from the Upazila Health Complex.

P2: If the prescribed medicines do not work or if there are a shortage of medicines, she refers to the Upazila Health Complex.

M: In that case, does she writes or mentions the medicine using a prescription or a paper?

P1: No, we go there and ask them to provide the medicine mentioning its name.

P4: In this situation, we tell them that we received three days of medicines from the CC and now, we are been referred to collect the rest of the medicines from the Upazila Health Complex.

M: In that case does she (the CHCP) write the medicine for you?

P4: No. She just mentions the name of the medicine.

M: Okay. Do people go elsewhere to collect antibiotics even if the supply of antibiotics is adequate?

P4: It varies from person to person. There are a few people who do not like the medicines provided from the CC as these are free—these are distributed from the Government. They believe that as the medicines are free; these will not work and might have less power. They usually consult with a private practitioner.

P2: Some people buy antibiotics from the pharmacy where the price may be 12 or 14 Taka per piece. They believe that since the medicines are costly, these are powerful and will be effective.

P4: These people visit the CC only when they suffer from normal diseases. If the condition is not normal then they would go to Daudkandi Private Hospital.

M: If antibiotics are available here (the CC), then?

P4: It doesn’t matter. They will go elsewhere to buy medicines.

P5: Earlier this was a very common problem. But now it has changed. People have started believing that the medicines provided by the Government are also effective.

M: As you said, who are these people in the community not interested in getting medicines from the CC?

P4: Mostly the rich and the educated persons avoid seeking health care from the CC as they think that they would not get proper treatment from here.

P2: They also fear that the care provider would not be able to diagnose their disease.

M: Is it easy or difficult to get antibiotics from every possible place?

P4: It is free here (indicating the CC) but if you have money you can get it from every possible place. It is easy to get antibiotics.

M: Where we can get antibiotics easily?

P4: Pharmacies at Daudkandi, private clinics and hospitals.

M: You mean it is easy to buy antibiotics from pharmacies and clinics.

P4: Yes. You may have to go a bit far from here.

M: Does it always require a prescription to get antibiotics from the pharmacies?

P4: Yes. They will not give you antibiotics without a prescription.

P2: Without a prescription, the pharmacist will not give you antibiotics because if they do this it would affect them.

P4: They will not give you antibiotics without a written slip mentioning the name and doses.

P1: They will give you medicines for primary treatment such as Napa, Histamine, Oral Saline etc.

M: Suppose, if you go to a pharmacy and ask the pharmacist to give you antibiotics by mentioning the name without the prescription, will he give you the antibiotics?

P3: In that case, if someone brings the blister of antibiotics to the pharmacist, they would provide it without a prescription. A couple of months ago, I went to a pharmacy and asked for some antibiotics by mentioning the name.

M: Okay, then?

P3: The pharmacist denied giving me antibiotics. He said that without knowing my health problem or complications he would not give me any medicine. After explaining my condition to him, he gave me antibiotics.

M: Was it without the prescription?

P3: Yes.

M: Okay. What people of this area usually do when the care provider does not provide any medicine or any antibiotics for the health condition they have visited the health care centre?

Or, if the care provider feels that they do not need any antibiotics and explains to the patients, do they accept the explanation or insist on giving antibiotics?

P4: They accept the explanation.

P1: Yes, they accept it.

P5: If people are given advice or explanations, they will accept it.

M: Why do you think that the people accept the care provider’s explanations?

P5: Because people have confidence in him.

P4: The doctor knows very well when a patient needs medicines and when it is not required.

M: Do the people of this area follow the advice or instructions provided by the care provider? Such as—completion of the full course, or not sharing medicines with others etc.

P3: They have to follow the advice.

P4: Most of the people follow instructions and some don’t.

P2: If they don’t follow the instructions regarding taking medicines and other advice, they will not be cured.

M: As you said some people follow instructions and some don’t, why is that and who are they?

P4: It varies from person to person. Actually, it is difficult to say who follows the instructions and who doesn’t. It is not even possible for the care provider to identify who doesn’t follow her instructions. Suppose, I have collected medicines for 5 days from the CC but after 3 days I stopped taking medicines as I felt better. So, it would not be possible for the care provider to know until I inform her. Similarly, it is not possible who follows instructions and who doesn’t.

P2: Mostly, the young people do not follow the instructions. They feel that it is better to consult with some specialists instead of the caregiver (the CHCP).

P4: Yes, they think that the medicines provided from the CC would not work.

P5: They want immediate results from any treatment.

P6: They assume that the medicines will not work as they are free.

M: Do the people of this area complete the full course of the medicines?

P5: Only the young people don’t complete the full course as they believe the medicines provided from here (the CC) will work slowly. They consult with an MBBS doctor again with the same health condition before finishing the full course provided from the CC.

M: As you said, they do not complete the full course then what they do with the leftover medicines?

P4: It is not possible to follow them.

P7: It cannot be confirmed whether they use or keep the leftover medicines or throw them away into the trash after consulting with the doctor.

M: Okay. What do you do with the leftover medicines or antibiotics?

P2: Actually, the care provider is providing medicines according to the patient’s requirement. For instance, if she feels that someone needs medicines for 5 days, she will provide only medicines for 5 days but not more or less than that.

P1: If there are leftover medicines at home, they will use them in future if they face the similar health condition without consulting with the care provider.

P2: Suppose, someone has given medicines for stomach upset and he could not finish them all. If any of his family members suffer from the same health related problem in future, it is likely that he would provide those leftover medicines to him or her. They share leftover medicines.

M: Could you please tell me what would happen if you do not complete the course?

P2: The disease will appear again.

P3: The virus will remain in the body if they do not complete the course. In future, no medicine would be able to cure the disease. The body will lose its immunity.

M: Have you ever heard about antibiotic resistance?

P2: We heard about antibiotics but never heard about resistance. This is the first time I heard about this term.

M: Okay. Does anyone have any experience that those antibiotics are not working now that has been used before to cure disease?

P1: No. We know that drugs do not work when expired.

M: Okay. Thank you for your time.
