# Supplementary material for: A process for developing a sustainable and scalable approach to community engagement: community dialogue approach for addressing the drivers of antibiotic resistance in Bangladesh
Source: BMC Public Health. 2020 Jun 17;20:950. doi: 10.1186/s12889-020-09033-5 (PMC7302129; doi:10.1186/s12889-020-09033-5)
Supplement: Supplementary file 10 — Additional file 10. CSG Members Male (5). Transcript of focus group discussion with male members of the community support group, region 5. [file 12889_2020_9033_MOESM10_ESM.docx]

| **Study name:**  Community dialogue for preventing and controlling antibiotic resistance in Bangladesh: Case for support | Interview ID: **CC- 5**  FGD (Male) |
| --- | --- |
|  | Date of Interview:  07/05/2017 |

**I= Interviewer**

**P= Participant**

**I:** So Could you please tell me what the name of this community clinic is?

P-1: x Community Clinic

**I:** Now you tell me, what is the system of administration? If we consider District at first, after that Upazila then?

P-1: Union

**I:** Then?

P-2: Ward

**I:** After ward?

P-2: Village. Previously here was 1 ward for 3 villages. Presently there is 1 ward for each village.

**I:** So ward comes after village?

P-2: Now a days each village is each ward. Presently, ward is village and village is ward.

P-1: Every village is different from another. There can be 2 ward in one village. There can be 2 village in 1 ward.

P-2: If it is large in size.

P-1: According to the number of voters.

**I:** Now I would like to know that how to segregate a village? Is there any ‘Para’ or ‘Mahalla’ ?

P-2: Yes, there are.

**I:** So you have para anad mahalla. How many para and mahalla do you have here? How many para and mahalla does each ward has?

P-2: There are 4 para in 1 ward.

**I:** So, there are 4 para. How have you divide those?

P-1: East side, West side, North side, South side.

**I:** Ok fine. Now I would like to know where and how the meetings about your health usually take place in this community. Does is held on?

P-1: Since the establishment of this clinic, so far we attended in 1 meeting only. We have discussed how to run this smoothly and how to help them.

**I:** One time?

P-1: yes, only 1 time

**I:** When does it take place? How long this community clinic has been established?

P-1: Approximately 3 years. But we have attended in 1 meeting only.

**I:** When did this meeting held on?

P-1: After 1 or 2 months once the clinic established.

**I:** So precisely there is no meeting usually arranged here. Do you arrange any meeting in your area if needed? For example, any kind of problem in village, any kind of development activities, or any kind of help arrives from outside which has to be distributed, like this.

P-1: We never had any meeting or discussion about health issue in the village. Like, we have mosque and graveCourt yard, or sometimes we get relief so we discuss about how to distribute it in para and mahalla. But we never had any discussion about health issue.

**I:** Okay we drop the health topic. As you said you discuss about natural calamities or relief or any kind of development activities. How do you arrange those meeting? Is it like “Uthan Type” (Court yard) meeting?

P-1: Yes it is something like Court yard meeting.

**I:** When does it take place in this case?

P-2: It’s not like always Court yard meeting. It’s something like when we go to market we meet each other and we discuss about different issues. Court yard meeting is one thing and when we discuss at market or bazar this is something different. When we do it at home it’s called Court yard meeting but when we gather at bazar it’s something different.

P-1: We usually arrange meeting based on mahalla or we villagers gather in a certain place.

**I:** Where does this meeting take place, the mahalla meeting? Is it take place at any particular house, Eidgah (where the Eid Prayer is held), any school any field or any bazar, where does it held on?

P-3: If it is about house matter then it will take place at house. Since we have our member and chairman in our village so if the issue is about village then we discuss with them. If it’s about the development of house then it will take place at house.

**I:** As you said the chairman topic, so where do you gather or arrange for meeting with chairman?

P-1: If the crowd size is large then it usually arranged in field.

**I:** School premises or playground?

P-1: school premises or madrasa premises

**I:** In that case which topic do you discuss in those meetings? What kind of discussion?

P-4: There are lot of problems in village. There are village arbitrations. Most of the time these sort of arbitration will take place. We usually discuss what kind or activities currently we have and how to distribute the responsibilities of the development activities that comes from union to mahalla. It is all about activities.

P-5: Different types of government work comes through chairman. We usually draw a finishing line of these meetings at chairman’s personal office or union parishad through discussion.

**I:** Which interval these meeting usually take place?

P-2: We have regular 3 meetings per week. Last Thursday of each month we have a meeting with all members.

**I:** This one is with member right. What I want to ask is, your own community meeting as you said about relief, about calamity these sort of meeting or anything about the development of village when does it usually arranged?

P-1: There is no particular time for this. If government provide any relief or if member brings these relief then only the meeting will take place.

**I:** It is about relief. If I consider anything about development of village or any monthly meeting, does it take place?

P-1: Not really, this sort of things does not held. It is usually discussed in union parishad.

**I:** Yes what are you saying?

P-5: If anyone think that they should gather with the honorable and senior person of village then they will arrange 1 or 2 meetings. But there is no particular time like per month 1 meeting it’s nothing like that.

**I:** What is the usual duration of these meeting? IS it like 1 hour or 2, 3 hours? What is the duration?

P-1: In our area the meeting will start around 3pm or 4 pm and it will continue till 5 pm or 5:30 pm.

**I:** Which means duration is 2 hours or 2and half hours. Does males and females gather together in this case?

Everyone: No, females and males gather separately.

P-5: Usually males take part, not females.

**I:** Does any separate meeting held for only males or for females? Does anything arranged like this?

P-2: If it is needed then members usually arrange this.

P-5: In our area we do not separately arranges this sort of thing for females.

**I:** What is the reason behind this system like no females will take part only males will? Is it anything your personal matter?

P-5: No one allow their housewife to let go outside.

P-2: No one will let the females come to the hall of audience.

**I:** You are talking about hall of audience. Suppose, I am discussing about family health service issue or anything about village development. Are females allowed in that case?

P-1: If the females come only. If your spokesperson or representative is female then our females will speak with those females.

**I:** But it will not arrange like meeting?

P-1: No, it is not like meeting. It is something like, some of your female spokesperson will come at one house, then some other village females will gather there and there will be a discussion or u can call meeting.

**I:** Whenever you come to meeting or announce for a meeting does everyone come?

P-2: What kind of meeting?

**I:** It can be anything. Suppose, it can be anything regarding village development like there need to construct a bridge, so everyone should gather together. This can be lot of things like this. How is the response or interest of villagers when you calls for meeting like heath issue? What kind of interest do you notice among villagers to attend those meetings? Is there any noticeable enthusiasm visible to attend there? Or anything like this is my personal matter whether I will go or not.

P-2: I give you one example, we do not need any bridge, we do not need any culvert. We have those in our village. But we need a Bridge made with bamboo (Shako). So we will call everyone’s help from both side of Kushiara. Some of them will provide bamboo, some of them will provide money, this is a process of stimulating everyone to take part for their own interest. But this thing is not so common in our area thus we don’t have any Shako.

**I:** What sort of interest are you finding here in this community clinic meeting arranged by me that you have gathered here?

P-2: my interest is to know and gather some knowledge how to run this clinic.

P-5: My uncle said we have got only one meeting where some of people will come from Dhaka. So we need to know what actually the thing is all about.

P-1: Definitely there is a reason behind this discussion. Why are they coming for? What reason? But we should know it from before. If our activities would carried out in a regular basis then we could have known it before. We could have known than some of guests are coming from Dhaka. They are coming for discussing about health consciousness or giving some solutions regarding health problems.

**I:** This one about today’s meeting that you are talking about. I have got it. But the reason I have thrown this question is suppose whenever you arrange a meeting, as you said meeting on relief, in that case people will gather there. Or if it is about development then they will gather or if any mitigation that time also everyone gather. Here I am going to every house to let them know there is a meeting so u guys come. None of us forcing anyone to come, everyone is coming from their own interest. Now my question is, what is that motivating or driving factor that energizing them to come?

P-1: Of course here is a reason. Like, today I have an objective to know, maybe there will be a discussion about clinic. Guests are coming from very far. Maybe they will enlighten us with various new information that we are unaware about.

**I:** It means interest to know why this meeting will take place. Now you tell me suppose, do the people face any obstacle when you call them to join? Or do they hesitate to come like, if the come they might face any problem? Have you faced this sort of obstacle?

P-1: No no. In our area everyone welcome and volunteer to any development activities. They will come with interest to know what fact is going on.

P-5: Most important thing is someone needs to invite us.

**I:** so u are telling about invitation. Now I would like to know from any of you how am I supposed to call you? Which one is the effective way to let everyone know so that they can attend? What am I supposed to do, any suggestion? Suppose we are going to arrange a meeting on next week (just for example) in this case haw can I let everyone know about this issue?

P-5: Here the important fact is, every one of us are the member of the community clinic committee. All the members of the committee along with the chairman and vice chairman of the council need to create an attention and everyone should shoulder the responsibility Responsibilities like, everyone should assemble to run a meeting from their own interest, or try to figure out whether everyone is getting the medicine at least once in a week, or is there any patient is depriving from getting treatment, if we can create an effective communication among us regarding these facts then I think we definitely can help to everyone whoever coming from outside for our sake.

**I:** okay fine. But u need to understand the question clearly. What I want to say is, I will arrange a meeting on tomorrow or on the next week. I want all villagers to know this information.

P-1: Whoever are associated with this committee, we need to join to spread it out.

**I:** Is there any other way to let everyone know?

P-1: All we need is the support of our committee.

**I:** How is that?

P-3: You are coming from Dhaka, whoever is responsible here….

**I:** Leave the topic of coming from Dhaka for a while. You just tell me, if I am also a member of this village, and I gave got a training from Dhaka, now I want to let everyone know about this on tomorrow or day after tomorrow. I have only 2 days in my hand. In these 2 days I will have to tell everyone about it. I will have to cover the whole area. How can I cover the whole area?

P-1: the responsible person of community clinic must spread out this announcement.

P-3: This may happen everyone got to know by us.

**I:** Okay tell me one method of this.

P-4: If any kind of meeting take place in the village that time the senior person of the area will tell everyone about it. Or any important issue will be announced at mosque.

**I:** Do you tell everyone at mosque?

P-1: through the imam of the mosque this sort of information will be announced. Imam will announce it using mike from the mosque that, today there is a meeting on health consciousness so everyone come and take part.

**I:** This one is by using mike. How do the people get information about the health issues? What are the sources of acquiring the information regarding health? Where will they get to know about it very first time?

P-3: Usually at very first time any announcement about vaccination or normal medicine people will get to know through mike.

**I:** you are speaking about mike but I would like to know that, where or whom the people get to know about the health information from?

P-6: initially they get to know from the community clinic. They get to know from the field officers.

**I:** who are the field officers?

P-6: Those who are responsible for health assistant. Or people can get to know from the members of FWA.

**I:** Is there any other way? Is there any pharmacy in this village?

Everyone: Yes there are pharmacies.

P-6: There are rural doctors.

**I:** Which means those who have opened a pharmacy?

P-6: yes

**I:** How have they become rural doctors? What is the reason that u call them rural doctor?

P-6: They have got training.

**I:** What kind of training do they have?

P-2 some of them might have completed LMF and after that they have done Diploma. Some of them might work with a doctor for long period of time.

P-6: Some of them have completed Paramedics course.

**I:** Are these the reason you guys call them rural doctors and they have pharmacies also?

P-2: Yes, some of them sits at pharmacy or some of them just have pharmacy.

**I:** is there any Homeopathy?

P-2: Yes there is.

**I:** Is there any Ayurvedic Practitioner?

P-1: There is no Ayurvedic Practitioner. People don’t believe on magic.

**I:** What are the other sources? Is there any possible way from where people get to know other than community clinic, pharmacy, and homeopathy?

P-6: People will get to know by their own effort.

**I:** Where they can get the idea of own effort? Who is providing it?

P-6: Through information technology.

**I:** What is information technology?

P-6: Through internet.

**I:** What else other than internet? Now you tell me, just you have said they will get different information about the health issues from different sources. How they the information? If we consider our CHCP, how do they provide the information, in which particular way? Do they just present it verbally? Do they use poster or leaflet? What is the particular way?

P-4: Most of those are provided orally.

P-6: Through calendar. There will be some message printed on calendar about child’s health. They will point it out.

**I:** Which of the source is mostly liked by the people?

P-6: Actually in case of remembering things we can use calendar. So people like this way the most. And people will easily forget the orally delivered information.

**I:** Which one is the most reliable way? Leaflet, poster or calendar? Which one is the most preferable?

P-6: Through Calendars.

**I:** Here is the picture and what else?

P-7: When I have to prioritize or give example from 2 point of view like if I hear, bird has taken my ear in that case before chasing the bird I need to check mu own ear out first. Another thing is there is a possibility of making mistake what people usually hear. But what people actually see they can remember it easily. So visualization is the effective way to remember.

**I:** We want to know, here you have said about the health officers who are appointed for this work. Some of them do their job for money, on the other hand some of them do it for their own wish which is volunteering. We call them as volunteer. Is there anyone who is a volunteer regarding health issue? Or is there any one volunteering for health in this community or in the entire village?

P-6: Usually those who are working as EPI officer they volunteer it. Are you talking about it?

**I:** Yes this is likely to happen that he/ she is working from his/ her own wish.

P-6: Another thing is suppose I know a lot of things regarding particular issue. So I can let others know.

**I:** This one also an example of volunteering.

P-6: This one is very few in number.

**I:** What is the responsibility of FWV or FWA? What does they do? What is the job of a health assistant or can call FWA?

P-6: The health assistant usually carry out the vaccination program and vitamin campaign. In these programs they provide initial treatment. FWA will assist them. They will provide monthly health treatment through this way.

**I:** You have said u have volunteers. How many volunteer do you have?

P-7: So far I know there is 1 volunteer.

**I:** Male or Female?

P-7: Female. Sorry we have 2 volunteers.

**I:** Are they females?

P-7: Yes, female

**I:** Is there any male volunteer?

P-2: No there is no male.

**I:** What is the job of these 2 volunteer? What usually they do?

P-2: They announce the vaccination program of the community clinic and they let everyone know irrespective of all areas. They will come to help pregnant woman specially if any woman suffer from labor pain at night. IF they are not able to give proper treatment to those woman in that case they will give them advises or they will go to clinic with patient.

**I:** Have you elected that person as your volunteer or she has just doing it by herself?

P-2: All of us support her for her working efficiency.

**I:** What is the reason behind supporting her?

P-2: Everyone rely on her and believe on her that she knows the work in proper way. If she has been called at the dark of night that time also she will go to help. She will be available at any time.

**I:** You have said u guys support her. Why do you support her, in which basis?

P-2: We just support her.

**I:** If u chose someone among you it refers you have elected her.

P-2: we can rely on her because she knows and perform the job better.

**I:** Does anybody supervise their job whether they are doing it in right way or not? Is there any system like that?

P-2: Yes we have our rural doctors presently. Like, whoever working at Kushiara bazar those who are giving initial treatment specially if they find any complicacy regarding treatment then they will call these 2 ladies.

**I:** Does anyone supervise these 2 ladies? Does anyone monitor their work whether they are giving proper treatment or not?

P-2: We are enough aware about their performances. We do monitor their job.

**I:** Who are those “we”?

P-2: For example I do monitor. There are few more people in this area.

**I:** Do you supervise them as a landlord?

P-2: No, I generally do it. I usually monitor them even before I have given this land and before the establishment of this community clinic.

**I:** Have you elected them only based on their efficiency of work?

P-2: yes, their efficiency is our center of attention.

**I:** Is there any other attribute other than efficiency for what they have been chosen?

P-2: They have got training.

**I:** How long will they work for? Is there any particular rule or particular working day?

P-2: Not really. They will always work. Whenever anybody needs them they will be available.

**I:** Suppose I require 1 or 2 volunteers who will give health treatment on antibiotic or who will be responsible for any other health issues. Now you will have to decide who will be those 1 or 2 volunteer from your community, who will have to work only 2 or 3 hours per week depends on that person. They will have to devote 2 or 3 hours in an entire week. Now whether he/ she will give this 2 or 3 hours in 1 day or 2 days that depends on him/ her. But he/ she must give this 2 or 3 hour.do you have someone like this among you? Can you provide us someone like this?

P-2: Yes we have.

I: Will they work according to this schedule?

P-2: Yes, they will work.

I: Which basis are you going to elect them? What kind of qualifications do they must have?

P-2: That person must have enthusiasm, educational qualifications, someone who have helping mentality.

I: Just you have said you have 2 volunteers who are working for you. What kind of stimuli they have got so that they are interested to work for others? Suppose I will work for you and to do so I must need something which will make me interested to do the work. What makes them interested to do it?

P-2: Whoever living in this area or mahalla, specially if a woman or child suffer from any illness or complicacy they will come to help us by their own wish.

I: I have got it. But as u said these 2 ladies are working for others from their own effort and will, they are giving their labor. What attribute actually makes them to do it?

P-2: They have friendly mentality.

I: What is that for?

P-6: Because they thing they will get blessings from God by doing it.

P-2: They do it for other’s welfare.

I: How will you encourage the person u have elected for this job? How will you bring out the internal motivation of that person or which particular factor will motivate that person?

P-2: we will talk to the person who will work for our health issues. We will encourage him/ her to work for the betterment of other people so that that they can be an idol. Like this other people will be interested in nursing others, they will work for health treatment. We will ensure that person if you do like this, you will be known as a good person, everyone will honor you.

I: Is there any link between health management and those who are working for health issues?

P-2: I have no idea about it.

I: Now you tell me how much area does community group cover? How many community support group do you have here? Could you please tell?

P-2: only 1 meeting has held since the clinic has been established. So I have no knowledge about it.

I: How long the community clinic has been established?

P-2: it is been 3 years.

I: Only 1 meeting had arranged about the clinic in these 3 years. Which topic have you discussed in that meeting?

P-2: Just we have formed a committee.

I: What was that committee?

P-2: Generally the committee was formed with the senior persons of the area.

I: What is the name of that committee? Is it community support group or community group?

P-2: Only community group.

I: Is not there any community support group?

P-2: Yes

I: How many members do you have in community group?

P-2: we were 7-8 members who have form this.

I: Well 7-8 members. How many members does community group have?

P-2: More or less like the same

I: Which means around 7-8 people.

P-2: we have never assemble later on so still the number of members are like this.

I: Does it mean there is no activity since the committee has been formulated?

P-6: We have noticed that people usually purchase medicines from here. So we also encourage people to come to community clinic.

P-2: We make the people aware about the initial treatment that has been given in community clinic. Or we let them know about the opportunities that the clinic and health workers are providing, like this we encourage them to contact here.

I: So you want to say this committee had formulate 3 years back.

P-2: This might be 3 years back or 2 and half years back.

I: So, committee has been formulated 2 and half years back and since then only community group has been formed and no other meeting has held on.

P-1: All the activities are going smoothly so no meeting has been arranged yet.

I: You could not say how many community support group you have now. You don’t have idea how many members are needed to form this either. Do you have any idea about the number of rightful position or the designation?

P-2: Here we might have chairman, vice chairman and then members.

I: Who is the chairman?

P-2: Chairman is our area member.

I: you are the vice chairman. What is the next position?

P-2: Member

I: Is there any particular group among members or everyone is just a member?

P-3: everyone is member.

I: How have you elected the members?

P-2: They are the honorable persons of the area according to their seniority.

I: Who have selected them?

P-2: We who are living in this area, we did it.

I: Which basis have you elected them?

P-2: We have found out they are interested to work.

I: IS there any particular rules and regulations of your work? Could you please tell me what the responsibilities are?

P-2: Responsibilities consist of monitoring the proper supply and storage of medicines, monitoring whether the patients are getting proper treatment or monitoring whether doctor is available or not.

I: Why didn’t you arrange any meeting after that? Why didn’t you monitor the activities properly? What do you think the possible reason can be?

P-2: Since we have a committee and they are carrying out their responsibilities properly, even we have not got any complain that any patient has been neglected. And they are efficient enough to run this, though we monitor this on a regular basis.

I: Can you tell?

P-1: Actually they also never call us.

I: What do you mean by “tara”?

P-1: CHCP. If he/she used to call us we would definitely come. We have just formed a committee. We do not have further more idea about this.

I: Have you never thought to go and monitor the activities from your own interest since you have formulated the committee? Haven’t you wanted to know the condition of it, no matter anyone one calls or not?

P-1: If there was a system of arranging meeting per month or in every 2 months.

I: Haven’t you ever tried by your own?

P-1: Everyone is busy with their own work so could not do more than this. And there are around 7-8 doctors available at Kushiara bazar. Most of the patients visit them. Moreover the health treatment is also available here. There is no scarcity of treatment over here. That’s the reason we haven’t gather.

P-6: Our people are getting the medication properly. If they have complained that they are not getting medicines then there was a need of arranging meeting.

I: I am still not clear about why haven’t you assemble yet. But I do think that since you have formed a committee you should arrange a meeting at least once in a month.

P-2: We should do it, we didn’t do it is our failure

I: Why haven’t you do it?

P-2: We are associated with the committee since it established but we didn’t do it because everyone is busy with their own work.

I: If the community group of community clinic and as we said our volunteer will arrange meetings and you will mediate those, the volunteers will carry out the meeting and some of your people will coordinate with community clinic, do you think is it possible?

P-2: yes it is okay.

P-6: it is important.

I: You have said about chairman and vice chairman of the committee. Where have you found the form of community group? Who have made it?

P-2: we have made it by ourselves.

I: Where have you found the concept of chairman, vice chairman, and landlord? Is there any category or any form have you got?

P-2: No we have not get anything like this.

I: Who has initiate the idea then? Who has framed the idea of community hierarchy?

P-4: After 2- 3 months of the starting, we have formulate this committee.

P-2: CHCP has said we need to formulate a committee. During that time we did it.

I: How did you decide which position is suitable for whom? How did you elect the person for each position?

P-2: Whoever were present here that time had decided it.

I: Was there any guideline from the upper level?

P-2: No, no there was no guideline like this.

I: You have said you never arranged any kind of meeting.

P-6: It sounds wrong that we didn’t arrange meeting. Everyone was busy so that it was not possible to arrange any.

I: Dose she (Pointing the health worker) asks you to come?

P-6: She did. She asked us to come couple of times.

I: So health worker has called you.

P-6: Yes she did.

P-2: They called us. One person came and ask us to go. We went and talked.

I: But you have never arranged any separate meeting.

P-2: No we have never arranged any meeting or discussion like this.

I: You have said you came here whenever needed. What were those needs that u came here?

P-6: I came here 2 months back. I saw our Haji (who has performed pilgrim) was present here to discuss about some issues. Like this I have seen him discuss about different issues around 2 to 4 times. I have just recently joined here.

I: So far I understand, you could not attend the meeting because you were busy with your own work. Even the CHCP didn’t ask you to come and join meeting right.

P-2: He used to call casually in case of any problem. Or when they get medicines that time they let us know to go and check the medicines from boxes.

I: If any problem arises regarding community clinic, how do you resolve it?

P-2: Here we haven’t faced any problem yet.

I: Now I am going to say is, your this community clinic is not functional, it’s not active. What do think, from which point of view you are not active? What were the reasons that you could not be active? I would let u know the reason I am asking this is, I will try to figure out the lacking and correct those steps here after. In future when we will carry out vast activities or do elaborate researches, we will keep this at mind.

P-2: Here we had limitations of time so that we could not provide enough time for meeting. Some of us is not mentally prepared. Someone might joined the committee but have lack of professional mentality. We haven’t thought these issues like this. For example, presently our major failure is we could not devoted it from the committee. Actually today we have found out that we had lack of consciousness. We have got it we were reluctant. We should have mentality of observing, consulting, assisting them at our convenient. We have lack of coordination among us.

I: Tell me some more opinions?

P-1: Whatever they he is saying is right.

P-2: My bottom line is, you will tell us how we can carry out our future activities, how we can work for the welfare and betterment of people and how to strive forward.

I: We will try it. Since we have started this movement hopefully we will work together. This our initiative and we will go ahead. Okay I have reached to the last question, after this we will be done with this. As we talked about volunteer, now would you tell me if we elect the volunteers from you will it be good for you to elect male and female volunteer separately?

P-2: Yes, It will be good.

I: Why do you think so?

P-2: Male volunteer will talk about man related stuffs and female volunteer will talk about femal’s issues.

I: What if males are dealing with females and females are dealing with males?

P-2: In our society it is not acceptable.

P-6: It’s about environment.

I: Is it taken as rule here?

P-2: Not rule.

P-3: If u bring 2 females here they will not talk in front of us.

I: Why won’t they talk?

P-3: They will say males are present here, so they should talk.

I: So if men are present then women will not talk.

P-2: They will talk only if everyone is woman.

I: So if I invite women in a meeting at a time if men are present there, then those women will not talk because only men will talk, is it?

P-2: If there are only women are present then they will talk.

I: You mean to say if we arrange a meeting which is completely separated for women then only they will talk. It is nice to hear. What do you think who can supervise the volunteer while carrying out the regular meeting help in community clinic?

P-2: One of us can supervise that volunteer. One of us will have the opportunity to monitor the volunteer.

I: Who is that “One person”? Who is well qualified for this?

P-2: We will provide one person who will always be able to give time.

I: Will you elect him/her from you?

P-2: He who has house near to clinic and can keep contact with clinic can be the supervisor.

I: Is he able to keep the records and information whether the volunteer is working properly or not?

P-2: Yes he can. If we explain him properly he can do it.

I: Do you think any of community member is suitable for this position?

P-2: We will provide some one from outside if needed. If that person perform his duty properly we will elect from outside.

I: You can elect from committee or you can elect from outside also.

P-2: Yes. But make sure he will be always available. He will be able to observe in a regular basis. If we elect someone from committee and after that he could not make adequate time then it is problem. So we can look for someone who is not form committee. So we are expecting someone who will perform his responsibilities accordingly.

I: Do you guys have any other opinion or advice or anything else would you like to say? What do you think about this movement? What else we can do regarding this initiative?

P-2: Only advice is this community clinic should run properly and smoothly so that our area people who are deprived from treatment will get the proper treatment. And we need your cooperation.

P-6: We should regularly assemble like today in each month or in every 2 months.

I: You will have to elect that. Because it is not possible for us to come here on every month. Since you are the dweller of this area so you will have to arrange the meeting. You will tell that elected person to participate in the meeting.

P-2: Even after that also we will have to maintain a good relation among us.

I: And when he will think (the elected person) it is necessary to assemble he will call you. Or if u decide to make a routine that on a certain date of each month or in the first week of every month we will assemble, you can go for it. So fine It was pleasure talking with you.
