# Supplementary material for: A process for developing a sustainable and scalable approach to community engagement: community dialogue approach for addressing the drivers of antibiotic resistance in Bangladesh
Source: BMC Public Health. 2020 Jun 17;20:950. doi: 10.1186/s12889-020-09033-5 (PMC7302129; doi:10.1186/s12889-020-09033-5)
Supplement: Supplementary file 11 — Additional file 11. Union Parishad Chairman (1). Transcript of interview with union parishad chairman, region 1. [file 12889_2020_9033_MOESM11_ESM.docx]

| **Study Name:** **Community Dialogue for preventing and controlling antibiotic resistance in Bangladesh: Case for Support** | **Interview ID:**  **CC2 Chairman** |
| --- | --- |
|  | **Date of Interview: 11/04/2017** |

I = Interviewer

P = Participant

I: What is your name?

P: My name is Z.

I: What is your position here?

P: I am the Chairman of …

I: Please, tell me the administrative breakdown of your area that is how you manage the administration of this area. If I talk about union, ward or para--- what is next?

P: Then only Ward.

I: Could you please explain it?

P: There are 9 wards in this area. Three female Members have been allocated to the three wards and the male Members have been allocated to the remaining wards.

I: Are these 9 wards belong to this Union Parishad?

P: Yes, these 9 wards are within this Union Parisad.

I: Is there anything like ‘Para or Moholla’?

P: No. There is nothing exists like ‘Para or Moholla’.

I: What is the smallest unit of a Ward?

P: It is only the village.

I: Okay, how many villages are there in a ward and how many villages within these 9 wards?

P: Each of the wards consists of two or three villages---sometimes four villages. There are some villages which are big and considered as a ward.

I: Okay. Now, I would like to know about the community meetings that are held in your area. Could you please tell me, what these meetings are and what types of issues are discussed in these meetings?

P: Actually, these are coordination meeting where we discuss about the lacking of doctors in the community clinic. Here, the doctors had not been recruited as required. The health assistant is providing services to the poor people in replacement of a physician. The community people are lacking the health services from a physician.

I: Okay.

P: And we also conduct these meetings to provide awareness about primary health care, particularly to the pregnant mothers.

I: Hmm, I understand. Okay, could you please tell me who organizes these meetings and also who are the participants?

P: An officer of the Upazila Health Complex usually conducts these meetings. The physicians do not participate in these meetings.

I: So, the officer conducts meetings.

P: Yes.

I: Okay. Who participate in these meetings?

P: Usually, they call me, some sensible inhabitants of the community and arbiters of the community.

I: Who are these arbiters?

P: They are the elites of the ‘para’ or ‘maoholla’ (the smallest unit of the village).

I: Hmm.

P: For instance, the Member of the ward and some old inhabitants.

I: Okay. Now, tell me, do they conduct separate meetings for males and females?

P: No, there are no separate meetings for males or, females. Males and females both participate together.

I: How often these meetings occur?

P: Usually, these meetings are conducted once in every two or three months.

I: What time of the day these meetings are held?

P: Usually, these meetings are held at 10 or 11 am in the morning and last for an hour.

I: Where these meetings are conducted?

P: These are conducted at the ‘Union Parishad’ (the office of the UP Chairman) or sometimes at the Community clinic.

I: Do they conduct any courtyard meeting?

P: Those who are involved with conducting courtyard meetings, they can tell about this. Since I do not participate in courtyard meetings; I have no idea regarding this issue.

I: That’s fine. In your opinion, what motivates people to participate in these meetings? What they think about these meetings?

P: Information regarding the child delivery, primary health care---actually, these issues motivate them to participate.

I: Is it difficult for the community people to participate in these meetings?

P: No. I did not find anyone who had faced difficulties participating in these meetings. They had attended every meeting, whenever they had been asked to participate. It never happened before that they are not willing to participate.

I: Okay. I am interested to know who provides health services to the people of this community. Could you please tell me about this?

P: I think, ‘F’ (the CHCP) is providing health services to the community people. In addition, ‘P’ also provides health services to the pregnant women in this area.

I: Who is’ P’? How does she involve with the health service?

P: ‘P’ is a health assistant. She works at the field level and she provides health information.

I: What information do these caregivers provide to the people?

P: Yes, they provide information regarding various health issues to the pregnant women and adolescent girls.

I: Okay. Do they use any photographs or, pictures or, posters or, calendars or, anything else while providing information related to the health?

P: Actually, I don’t have any idea about this.

I: Okay. We are conducting a study in which we want to educate people about the correct use of antibiotics. So, we need some volunteers from this community to participate in this study who will provide information regarding the issue.

P: Okay.

I: So, we are thinking if you could give us some volunteers who are willing to participate and educate people would be better. We would involve some volunteers. Are there any volunteers exist in this area?

P: There are some young adults in our area who are willing to participate in social works if they get the opportunity.

I: At present, do you have volunteers who provide health education to the people of your community?

P: As per my knowledge, there is no volunteer in this area.

I: Okay. Now, in your opinion, what would be the criteria for a volunteer to be recruited?

P: They should be provided with training, so that they could educate pregnant mothers accordingly. They should be educated and should have the ability to explain the issues to others.

I: Okay. So, they should be literate and should have the ability to explain the issues to the people. Is there anything else or any skill?

P: They should be social.

I: Okay. Now, I am interested to know about the community group or community support group of the community clinic. Could you please tell me about this?

P: No, I do not have any idea about this.

I: Okay then, I am going to end this interview here. Thank you so much for your time.

*Note: Para or, Mahalla is not a part of the administrative breakdown and this could be a reason that the chairman being the part of the administration did not mention it. However, since Paras/Mohollas are a way that the local people follow to identify their locality therefore the chairman had mentioned it later.*
