# Supplementary material for: A process for developing a sustainable and scalable approach to community engagement: community dialogue approach for addressing the drivers of antibiotic resistance in Bangladesh
Source: BMC Public Health. 2020 Jun 17;20:950. doi: 10.1186/s12889-020-09033-5 (PMC7302129; doi:10.1186/s12889-020-09033-5)
Supplement: Supplementary file 12 — Additional file 12. Union Parishad Chairman (2). Transcript of interview with union parishad chairman, region 2. [file 12889_2020_9033_MOESM12_ESM.docx]

| **Study Name:** **Community Dialogue for preventing and controlling antibiotic resistance in Bangladesh: Case for Support** | **Interview ID: CC5 Chairman** |
| --- | --- |
|  | **Date of Interview:**  **07/05/2017** |

Information about the interviewee:

I = Interviewer

P = Participant

I: What is the name of your Union?

P: It is ...

I: Could you please tell me the administrative breakdown of your area? Let’s consider the district level first. Then what comes next?

P: Then comes the Upazilla.

I: Then?

P: Under the Upzilla there are Union Parishad.

I: How many villages are there under this Union Parishad?

P: There are 13 villages and 9 Wards under this Union Parishad.

I: What comes next under the Union Parishad; Wards or Villages?

P: Ward comes next under the Union Parisahd. Mostly, villages are referred to as Wards on the basis of the extent of the periphery.

I: So, there are 13 Wards under your charge, right?

P: No, there are 13 villages and 9 wards.

I: Okay, are there any small units under the villages like ‘Mahalla’ or, ‘Para’?

P: Yes, there are Paras or Mahallas. Each of the villages has ‘Paras’ or, ‘Mahallas’.

I: Do the people of the Wards under your charge conduct health related meetings?

P: Yes. For instance, the village doctors do this.

I: Who are they?

P: They are the village doctors. The FWA (Family Welfare Assistant) visit all the houses of the villages and provide health education related to pregnancy.

I: Do they provide the health education through courtyard meetings?

P: Yes, they do this by conducting courtyard meetings.

I: What are the health issues do they discuss in these meetings?

P: Mostly, they discuss about female health issues. They talk about the menstrual problems, urinary tract problems and the safety measures during pregnancy.

I: Who organizes these meetings?

P: Sometimes, these meetings are conducted by some of the NGOs and sometimes by the health workers.

I: Who are these NGOs?

P: Different NGOs conduct these meetings at different times. Workers of the NGOs like CCD or, SHISHU are involved in this.

I: Are there any NGOs who are also involved in conducting meetings?

P: Yes, ASHA is another NGO who are also involved in this.

I: Who are the participants of these meetings?

P: Females mostly attend these meetings.

I: Do males participate in these meetings too?

P: No, males do not attend these meetings. If males attend these meetings then the female participants would not talk as they would feel shy in front of the males.

I: Okay. Are there any separate meetings for males?

P: I have no idea if they conduct any meetings for the males separately.

I: How often do these meetings occur? Every month or, week? Usually, when these meetings occur?

P: I have seen that these meetings occur frequently but I don’t have any idea about the meeting schedules.

I: What time of the day these meetings are usually held?

P: Usually, these meetings are held in the evening or, after 12 noon.

I: How long do they last?

P: They last for an hour.

I: Could you please tell me, what encourage these women to participate in these meetings?

P: Women receive various information regarding the safety or, precautions during pregnancy which encourage them to participate in the meetings. Earlier, they had no idea about this. Besides, people used to have more children. Now, they are aware of family planning –some have one or two kids and some use permanent contraceptive method –this also encourages them to take part in the meetings.

I: Did they face any challenge in participating the meetings? Or, what prevents them in participating the meetings?

P: There is nothing like that. Nothing is preventing them in participating the meetings in this area. Though the people of this area are religious, they are very concern about the health issues. Sometimes, the Imam *(religious leaders)* also educates people about family planning by reciting some verses from the Hades.

I: Okay. In your opinion, what would be the best way to reach the whole population of this area if I want to provide them any health information or if I want to involve them for a particular task?

P: It’s the same. You can inform everyone through court-yard meetings.

I: How is it possible, please explain.

P: You can do this by selecting a person and giving him the responsibility to inform everyone. The person would inform everyone by saying that on behalf of the Government, a team will visit this area to create awareness regarding health issues through a courtyard meeting. Then everyone would participate. At least, seventy percent people will participate.

I: Who could inform the people?

P: The village police can do that. If you ask the village police or the Member of the Ward to inform everyone, they can inform the entire inhabitants of one or two villages in a day. Or, they can cover a Ward in a day.

I: Okay. Do you think that everyone including males and females would participate in the meetings?

P: It would be difficult for everyone to participate. Everyone is busy with their own work.

I: We might organize consecutive meetings.

P: Then there will be the likelihood that everyone would participate.

I: Earlier, you told me that people of this area receive health information from the NGO workers or from the FWAs. Apart from this, are there any other ways for the people to get health information?

P: Yes, they can also be informed by publicity.

I: Okay. But I was asking that alike the NGO workers or FWVs are there any possible ways that the people of this area would receive health information?

P: No, there are no other possibilities for the people to receive health information. Only literates would learn about the health related issues from the books.

I: You also told me about the Imams.

P: Yes. The Imams should also take the responsibilities for the betterment of the people. They can take the responsibility to discuss health issues with the men since they go to the mosque in every Friday to pray. It would be better if the Imams take the responsibility to publicize the issue. I think Imams would be the easiest group to execute such type of social work in our country.

I: Is there anyone else who could also be involved to educate people about health issues?

P: The social workers or the social leaders could also publicize health related issues through community meetings. These meetings are held once or twice in every month. They can create awareness among people by explaining the issues in each meeting for 10 minutes.

I: How often these meetings occur in your area?

P: Usually, these meetings are held once or twice in every week in my region.

I: Are these courtyard meetings?

P: These are not courtyard meetings, rather these are arbitration meetings. These meetings are conducted to settle the internal problems between inhabitants. We arrange these meetings when a problem needs to be solved.

I: Where these are conducted?

P: These meetings are conducted in someone’s house or at the school ground or, sometimes at the Union Parishad office.

I: You told me earlier that the NGO workers or the FWAs are delivering the health education. Now, could you please tell me in which format do they deliver the information? Is it through discussion or, any sort of printed material?

P: Sometimes they provide leaflets and sometimes they deliver the information through discussion.

I: What do you think people trust the most, both in terms of people delivering the information and in terms of the format of the information?

P: People like the discussion most. Those who are not be able to give time to discuss due to their work would prefer receiving leaflets or posters. I think both discussion and leaflets would play an important role to aware people.

I: Okay. I want to know one thing. As you said, there are health workers such as NGO workers or, FWAs who deliver health information to the people. So, are there any volunteers in your area who deliver health information or support people in health issues?

P: Currently, there are no volunteers in this area. Earlier, there were some volunteers. Now people are very busy with their regular work.

I: When was it?

P: There was a village doctor known as Doctor M who used to deliver and explain health-related issues to the people voluntarily. He was a retired lab technician. People of this area received information regarding health issues in his presence.

I: Do you have any volunteers now?

P: No. No one is working voluntarily.

I: How was he selected to provide health education to the people?

P: No one selected him. He used to do this in his own interest.

I: Could you remember how he used to deliver health related information?

P: He used to call us to sit with him to discuss family planning issues and preventive measures of various health related problems. I think the current village doctors could also do the same.

I: What do you mean by village doctors? Who are they?

P: They are the Government registered paramedics who practice in pharmacies. Some are not Government registered. Those who are registered practitioners can take this responsibility. For instance, there was another doctor who had a pharmacy at Kushiara Bazar and known as Doctor K, used to deliver health related information to the people voluntarily.

I: Was he a volunteer?

P: Yes, now he is not there anymore.

I: What had motivated both Mr. M and Mr. K to work for the people voluntarily?

P: Their motivation was only to help people of this area. They believed that their hard work would benefit someone and people would remember them.

I: Okay. Since, they were working as volunteers, who supervised their work?

P: No one supervised their work. They did this voluntarily.

I: We are interested in working on antibiotic resistance here. We want to discuss with people about the use of antibiotics and how inappropriate use of antibiotics could affect our family.

P: Alike family planning, not only antibiotics but also people should learn about the use of various types of medicines.

I: Yes, coming to your point, we want to educate people about the use of antibiotics. In order to do that, we need 2 or, 3 volunteers to be involved from your area who would spend 2 or 3 hours in every week to educate people about the use of antibiotics. He or she will educate every inhabitant through organizing meetings.

P: He or she will educate people about the correct use of antibiotics, that is, which antibiotics are good, which antibiotics should not be used and which antibiotics will work or, which will not etc.

I: We would provide the training to the volunteers. After receiving the training he or she will educate people of the villages in every week for 2 or 3 hours. Now, what do you think about this idea?

P: This is a good idea. If you do that then every single inhabitant of the Paras would receive information about antibiotics. It would be effective if you do this in each Para separately. For instance, you can conduct 2 meetings in two Paras in two alternate times. It is important to cover each Para separately.

I: So, if we want to do this, can you involve some volunteers from your locality?

P: Yes, obviously. There are some educated young in my area who are enthusiastic and willing to work voluntarily.

I: As you said there are some young willing to work voluntarily, in your opinion what are the skills they would require to be selected?

P: At least they should pass SSC or HSC examination and it would be better if the candidate is a graduate. Those who are literate will understand it very well. They would be able to explain the health issues to others. We will need such volunteers.

I: Yes, since you are going to choose them from your locality, it would be better to set selecting criteria based on which they will be invited to be involved.

P: Hmm. He or she should be educated and should be accepted by all. Some people participate in the meetings willing and some don’t. We have to select someone who will be accepted by all and everyone would participate in the meeting whenever he or she asks them to.

I: In that case, do you think male and female volunteers need to be chosen separately?

P: Since the issue is about antibiotics, I think we can choose both male and female volunteers together. And if the volunteers have to discuss the issue with the community people then it will be better to select male and female volunteers separately.

I: Why do you think that? How would this selection benefit the community?

P: Woman trusts another woman. Sometimes, they don’t trust man. So, if we select them separately, it would be acceptable by all.

I: Considering the earlier discussion on the method of delivery of the health education, you told me that people prefer discussion. Why is that?

P: The illiterate people trust discussions since they are unable to read. If you provide them any leaflet, they might through it away. They will memorize everything you ask them to do. Oppositely, educated people would read the leaflet. So, it would be better to discuss the issue with others who are unable to read.

I: Okay. Suppose, you have involved two volunteers separately, that is male and female volunteers. Now, in your opinion, who should supervise their regular activities?

P: You can do this. If you do not want to do that then the Chairman or, the Member of Ward can supervise their regular activities; whether they are performing it properly or not.

I: Suppose, they have to conduct 4 meetings in each month. So, do you think the member or the chairman can supervise these meetings? Or, anyone else should take the responsibility?

P: The member can take the responsibility to supervise the volunteers. There are nine villages in this ward. If you select two volunteers from each of the villages then there will be eighteen volunteers. You can also select one from these eighteen volunteers and can give the responsibility to supervise the rest.

I: Okay. Now, please tell me about the community clinic in your area. Did you visit the clinic?

P: Yes. In order to, understand their activities I have visited the clinic twice.

I: Do you have any idea about the community group and community support group?

P: Yes, I have seen that people are treated very well and they are also given free medicines from the community clinic. The people of this community are very happy.

I: Yes, but I want to understand if you have any idea about the community support group or about the community group; that is, how many members are there in the community group or community support group?

P: No, I do not have any idea about the community group or community support group.

I: You don’t have any idea who is looking after the community clinic or who supervises the clinic?

P: No, I don’t have any idea.

I: Hmm. Okay. Since, you do not have any idea about the CG or CSGs of the community clinic; we are going to end our discussion here. Thank you very much for your time.

P: Thank you too.
