# Supplementary material for: A process for developing a sustainable and scalable approach to community engagement: community dialogue approach for addressing the drivers of antibiotic resistance in Bangladesh
Source: BMC Public Health. 2020 Jun 17;20:950. doi: 10.1186/s12889-020-09033-5 (PMC7302129; doi:10.1186/s12889-020-09033-5)
Supplement: Supplementary file 13 — Additional file 13. CHCP CC1. Transcript of interview with community health care practitioner, region 1. [file 12889_2020_9033_MOESM13_ESM.docx]

| **Study Name:** **Community Dialogue for preventing and controlling antibiotic resistance in Bangladesh: Case for Support** | **Interview ID: CC1 CHCP** |
| --- | --- |
|  | **Date of Interview:**  **09 /04/2017** |

Information about the interviewee:

I = Interviewer

P = Participant

**Part One: Antibiotic Use**

I: Please can you tell me what people in this community do when they are not feeling well?

P: People usually visit community clinic, Upazila Health Complex, Dhaka hospitals, Gauripur Bazar where medical officer and paramedics are available. There are almost 80 private clinics in Gauripur Bazar.

I: Why do they do this?

P: In Community Clinic, this is a place for the rural people where they get free medicines. Patients visited Upazila health complex when I referred to them or when nearby their home. Elite people who can afford visited Gauripur Bazar or referred in special cases or if the diseases are critical. When Community Clinic closes after 3 pm they visited to the paramedics or when they live nearby or during an emergency. Elite people visited Dhaka.

I: Do different types of people do different things like children aged 1-5, other children, and pregnant women, women of reproductive age, men of working age, older women, and older men ?

P: I think people do different thinks for children , also depending on severity and socio-economic status. In Community clinic they immediately visited here. I referred to Upazila Health Complex if required. If people are rich or the case is severe then I refer to Dhaka or Gauripur Union. For adults this is also depend on socio economic status and referral. People with higher socio economic status are referred to Gauripur Bazar.

I: Do you always prescribe medicine?

P: Actually I prescribes medicines in case of cold, cough, and fever.

I: Please can you tell me which antibiotics you most often prescribe, to whom and for what conditions?

P: I provide Amoxycillin 250 mg and Cotrimaxozole for cold, cough, wound, fever, infection, ear-ache

I: When you do need to prescribe antibiotics, what do you usually tell the patients about taking their medicine?

P: I generally instigates fear inside the patient, so that the person understands the importance of taking the full course of medications. I tell them, that if they don’t complete the course, it could reduce the effectiveness of the drug in future, and thus, the antibiotic wouldn’t be effective for the same complaint. I also explain the process of taking medicine to the person.

I: Do you provide any advice about sharing medicines with anyone else?

P: I always advises that the same medicine cannot be used for different people, as they could have different conditions and also advices that same medicine can’t be used for the children.

I: Do you provide any advice about keeping leftover antibiotics if they didn’t complete the course?

P: I advises them to complete the course. . In case of leftover medicines, if the patient asks me whether I can have those, I tells the patient to bring the medicines, and checks for the expiry date. If the drugs have not expired, I asks them to have it, if they can be consumed as per their condition.

I: Do you provide any advice about the correct dosage of medicines for how many days and how many times a day?

P: I explain about the doses of medicines and how to consume.

I: If the supply is inadequate, what do you tell the patients to do?

P: I provide full course of Antibiotics for 5 days because if I provides them only for 3 days they might not come again to complete the full course. In case the medicines are not available, I refers the patients to the UHC. In earlier cases when I used to give medicines for 3 days, and if the medicines were not available at the cc, I would refer the patient to the Upazila health complex, with a referral slip, where I would mention the name and dose of the drug, and the condition it was given for, in order to prevent confusion.

In some cases, I also asked them to take the empty blister packets to the pharmacy, if the medicines wouldn’t be available at the cc.

I also mention that to take vegetables.

I always advised the patients to take another person when he/ she visited the Community clinic because the patient may forget the drug, but the attendant can help them to remember the medicines.

I: If patients do need antibiotics, then where do they get them from?

P: Community clinic, Upazila Health Complex, Paramedics, Gouripur bazar, from Pharmacy Antibiotics can be purchased easily at the pharmacy if the name of the antibiotics can be mentioned.

I: Can you tell me whether people can acquire antibiotics without a prescription and how easy or difficult that is?

P: At Community Clinic – it is given to them for a period of 5 days, as they wouldn’t come back after 3 days. At Upazila health complex - on having a written prescription. At pharmacy adjacent to the Paramedics, people can get it if the paramedics have prescribed. At Gouripur bazar some get antibiotics, after having a written prescription, some get if the name of the antibiotic can be mentioned, even if the prescription is not written.

I: Is a prescription always required?

P: The pharmacy can give the antibiotics to the patients, without prescription, if the patient can mention the name of the antibiotic. The patients have been advised not to ask for medicines by name. Rather, I advised them to explain the symptoms, and the drug would be given to them accordingly.

I: Can you tell me about how patients respond if you tell them that they do not need antibiotics, do they accept your explanation?

P: There are some who’d listen to the advice of me, and understand that the medicines haven’t been prescribed because they were not needed.

I: Do they insist or become angry?

P: In my opinion, some patients would get angry/agitated. In some cases, I try to explain the reason to the person. However, in some cases, if the patient don’t listen, and usually the patients who are old, then I checks his/her blood pressure, takes a history, and may give vitamin supplements, or even saline.

I: Do you think that they go somewhere else and, if so, where do you think they go?

P: When I refer.

I : Do you think they use leftovers or share with other people?

P: Actually I think that they shared leftover medicines, mainly general medicines. I also thinks it can happen also in antibiotics.

I: Do you think that patients do as you advise them to do? Why or why not?

P: As per my opinion maximum patients complete their courses. However, there are some who stop taking medicines once they start feeling better.

I: Do patients keep medicines for later use (for themselves or others), do they share them?

P: I am not sure Patients may share their medicines with other persons of their home if they get sick. Maybe it’s possible.

I: Do you think they use leftovers?

P: They may, if they start feeling better.

I: Do you provide any advice about the correct dosage of medicines for how many days and how many times a day?

P: Yes, Patients follow my advice about doses of general medicines. But for antibiotics everyone does not follow my words because of lack of knowledge about antibiotics.

I: Have you ever heard the term “antibiotic resistance”? Can you tell me what you know?

P: I know about what is antibiotic resistance and I creates fear among people. It means that bacteria have changed in response to the medicines that we use to treat the infections that they cause.

**Part two : Potential Intervention**

I. Can you tell us whether the people in your area are given health related education in any other way?

R: in any other way....I don’t get your point.

I. Suppose like, the court yard meeting......

R: There is a matter of the court yard meeting but it is not organized separately.

I. Who is responsible for organizing this court yard meeting?

R: It is actually the responsibility of my community group and community support group members.

I.If they do not organize those meetings, then who will do it?

R: These are sometimes I have to do but basically they are doing now

I. Is this community group and community support group members able to conduct court yard meeting properly?

R: In my opinion, they can do it properly otherwise so many patients did not come here (in the community clinic).

I. That’s great. Now can you tell us, where and how they arrange any court yard meeting for village people?

R: They usually organize those meetings to go to different homes. At that time, if they found 4/5 people were available at someone's house, and then started discussions there. No place is chosen for this meeting separately.

I. Do they start talking straight away?

R: No, just a discussion cannot be initiated like this way. Firstly, they tell the people what they want to talk about. After managing everything they will proceed according to their topics. For example, they say that on such a day, worm tablets or vitamins capsules will be provided in such a place. On that day you will bring your children to feed them.

I. This is awareness. But do you know whether there is any court yard meeting with health education?

R: Actually it's about to happen. The things I used to tell them they are instructed to say those things at court yard meeting. I asked them to with general people about high blood pressure, diabetes. What are the symptoms of those diseases, what are these diseases, what food they eat if they become affected by those diseases? I also tell them that this is how you know the information that you will tell others in the same way in the village.

I. Well, your community group and community support group meet people at their home yard, but have you ever arrange any meeting with all the people of village at your community clinic?

R: We don’t have such meeting here.

I. Well, if there is an accident, when everything is to be told together or you have to say something by miking then what do you do?

R: There has been no incident so far but I cannot say what will happen in the future.

I. That's good but you do not have to do any such job during the national elections?

R: No, we have no relation with any work of national election.

I. Well how many of your meetings are arranged at time interval and how long the duration of those meetings?

R: It depends on the topic, but usually the meetings are for 30 minutes.

I.Are there any other activities related to community groups and community support groups, except for talking about various health issues organized by the meeting?

R: Yes they have, Such as, delivering various health messages to people. But I cannot tell each other alone. They can go to people's house and deliver them the messages.

I. Are these meetings organized for men and women separately?

R: No, meetings were arranged with men and women all together

I. Brother, you said that information about the antibiotics is not reaching to the more than 6,000 people. For this reason, if some volunteers are recruited from the government, then to assist in community group and community support group members. Do you think it will be okay?

R: I don’t get your point. Would you please repeat once the whole thing?

I. The whole thing is like that, we will recruit some new volunteers and then trained them like community group and community support group members and then send them to the community to disseminate the information about antibiotics?

R: I think it will be great

I. So do you think these volunteers are better off taking from community group and community support groups or better if they are taken from the public?

R: According to me, community groups and members of community support groups know a lot about it. Now if you think that they need to be more skilled then you can train them up more. There is no need to take people from outside. But if you feel that knowing about Antibiotics equally important to community group and community support group members as well as outside people, then you can take volunteers from general people.

I. Actually, I think that the responsibilities of community members and Community Support Group members are much more. So we want to recruit those volunteers to reduce their workload. But community groups will monitor all volunteers’ work. Then what will be the whole thing?

R: It should be good, however, to keep in mind that community groups do not dominate volunteers. If this happens, the whole thing will turn the other side.

I. Now I understand. Would you please tell us what will be better to execute the whole process? See, you have a village, a house, a ward, so how is it better to choose volunteers? Because we do not have any idea about this, but there is a desire to implement the plan. So, we want to know your opinion.

R: You can make the whole process like us. As you say, you may choose an Imam as a volunteer and told him to tell the common people about antibiotics. He is praying 5 times a day. After every prayer, if he tells this information to the person, then many people will come together to know. And people respect the Imam, so they will listen to him.

Besides this, if you choose a teacher as a volunteer, then people will listen to him because he is a respected person. If he will say something to someone he/she will take it seriously. Or you can request the local politicians to be a volunteer of your programme. He might be influential in this matter.

I. Actually, I want you to know that if we want to reach out to 6000 people with some health related information. But members of community groups and community support groups are not able to reach everyone this information. Only to depend on them, the whole work may stop. So we want to get some volunteers to help them. Now our question is how can we choose these volunteers or on what basis?

R: In my opinion, it would be better to take the respected person of the society because everyone listens to them

I. Well, if we tell the person that you have to discuss health related issues with general people for 2 hours every week. Do you think he can do that?

R: He cannot do this because 2 hours a lot.

I. So, how much time would be better for the meeting?

R: In my opinion, it would be better to arrange each meeting twice a month without meeting every week. Then they may be able to give time.

I.Who will be able to conduct a meeting in the week?

R: None other than the common man will do this work every week.

I. If you have to choose a volunteer from the general person, then what do you think will be good to choose based on?

R: Among the general people, I want to mention about farmers, kindergarten school teacher, NGO’s employees. Because after doing their own duties they have time. At that time they can say these things to the people.

I. You talked about NGOs. Which NGOs are working here?

R: There is no other NGO except BRAC. BRAC staff is very aware. They can do any duty very well. You can take some volunteers from BRAC.

I. I want to know based on what these people can be selected?

R: Since this person has to talk about antibiotics, that person needs to be educated. So I think educated people will have to choose.

I. In your opinion, it is good to select a boy or a girl as volunteers?

R: I think it would be better to choose both the boy and the girl because it is very difficult for the boys to send a message to the girls, but a girl can do it easily. Moreover, it is difficult for girls to go to remote areas, which is not very difficult for a boy.

I. Do you have a volunteer system in your community clinic?

R: We do not have any volunteers but there are volunteers in the immunization program.

I. Do you know how those volunteers are selected?

R: So far I know, they have free training then they are selected as per need.

I. If we take volunteers like this, how can we encourage them to do their own duty?

R: Always keep in touch with them. They have to train for at time interval. In order to encourage them for training you can arrange transportation or tea breaks. You can also arrange some interesting activities for them.

I. Interesting activities!!! Would you tell us more about this?

R: You can arrange entertaining dance /songs for them occasionally. Suppose one day after training you can organize an indoor game-show such as a pillow game. In that match, you can arrange small rewards for the winner.

I. Do you know what the volunteer means?

R: Yes I know. A person will do any humanitarian work without payment.

I. That’s true. The whole process depend on his/ her motivation whether they will continue this volunteer work because you know at the end they will not get anything except mental satisfaction. But how long that satisfaction will sustain that is a big challenge. So my question is what we can do to encourage them to do their own duties without any expectations?

R: In my opinion communication is the most important thing; what I want to say, maintaining good relations with the volunteers. We have to prioritize their opinions while arranging any meetings. Besides, I cannot provide tea and breakfast for the members of my community group and community support group after meeting or training because the government does not give any money for this purpose. But this small arrangement encourages them much more.

I. We want these volunteers to enter the health system that means they should always be in this system. So we want someone to monitor their activities. In your opinion, who would be best to give supervisory responsibility?

R: That's right, Apa, their activities must be supervised. In my opinion, the chairperson of the Community Group will be best given the responsibility because he/she is also a public representative.

I. Brother, the facilities you are offering to the Volunteers, do you think those facilities can link to the community group with the Volunteers?

R: Yes I think it could be possible. There is training for Community Group and Community Support Group members every month. Then we could involve the volunteers with them.

I. As you have arranged those meetings, whether you have recorded them anywhere?

R: We keep records of everything in the registry.

I. Who has kept the records? Do you?

R: Yes, I am.

I. If you want to record the work of these volunteers, then what would be better to do?

R: It seems easy for us to record documents. Then these papers will be in the specified file. And the volunteers have to set the deadline for completing the work. For example, these ten people will be served with information in a specific time. As well as the name and mobile number of those ten people will be collected, so that later on, it will be known that the volunteer is working properly.

I. Do you think that going to everyone's home would be better to say this information or to gather all the people together?

R: It is not possible to tell everyone at home, I think it would be better to gather some people and then to say those information.

I. Do you think it would be better to promote this information by talking or in any other way; For example, by showing different types of pictures or by any other means?

R: If you present this information in a practical way I think that would be better.

I. in a practical way!!! I don’t get your point-

R: It's like the Inner Roll Play. For example, a Volunteer will act as a doctor and another volunteer will as patient then they will talk about health issues. In this way people will remember these words very easily. Besides this, you could present that information by showing picture or through a video presentation.

Thank you so much for your kind co-operation.
