# Supplementary material for: A process for developing a sustainable and scalable approach to community engagement: community dialogue approach for addressing the drivers of antibiotic resistance in Bangladesh
Source: BMC Public Health. 2020 Jun 17;20:950. doi: 10.1186/s12889-020-09033-5 (PMC7302129; doi:10.1186/s12889-020-09033-5)
Supplement: Supplementary file 14 — Additional file 14. CHCP CC4. Transcript of interview with community health care practitioner, region 4 [file 12889_2020_9033_MOESM14_ESM.docx]

| **Study Name:** **Community Dialogue for preventing and controlling antibiotic resistance in Bangladesh: Case for Support** | **Interview ID: CC4 CHCP** |
| --- | --- |
|  | **Date of Interview:**  **04/05/2017** |

I = Interviewer

P = Participant

**Part One: Antibiotic Use**

I: Please can you tell me what people in this community do when they are not feeling well?

P: People usually visit community clinic, Union Health Complex which is 20 bedded hospital, Daudkandi, Pharmacy. There is no traditional healer in our community.

I: Why do they do this?

P: When Community Clinic closes after 3 pm they visit to the pharmacy when they live nearby or during an emergency. Educated people visit Union health Complex for better treatment and also feel that they will get better service from there.

I: Do different types of people do different things like children aged 1-5, other children, and pregnant women, women of reproductive age, men of working age, older women, and older men ?

P: I think children and women mostly visit the community clinic. Men (age 20 to 50) mostly visit Daudkandi because they work over there; so they visit UHC and seek treatment over there if needed. I refer to Union Health Complex if required.

I: Do you always prescribe medicine?

P: Generally, I prescribe medicines in case of cold, cough, and fever, headache, allergy problem which is very common in our community. I suggest paracetamol for normal sickness such as children’s cold, cough for one day and counsel them. If not cured after 2 days I advise to come back again with children.

I: Please can you tell me which antibiotics you most often prescribe, to whom and for what conditions?

P: I provide 5 types of antibiotics such as Penicillin, Doxycyclin, Metronidazole, Amoxycillin and Cotrimaxozole for cold, cough, wound, fever, infection.

I: When you do need to prescribe antibiotics, what do you usually tell the patients about taking their medicine?

P: I generally advise to complete the 7 days course. At first I provide 5 days medicine, then suggest to come back after 2 days to take the medicine. I always advise patients to understand the importance of taking the full course of medications for 7 days. I tell them that if they don’t complete the course, it could come back again. Sometimes patients misunderstood the medicine doses and side effects. I advise to take paracetamol, Salbutamol and amoxicillin for 5 days and advice to come back after 5 days and also share the side effects of salbutamol and also advise that if the side effects occur from salbutamol, please stop only that medicine. But she (indicating the patient) often continue to take medicines for 3 days but not 5 days because of the side effects of salbutamol. Bur she stops salbutamol and amoxicillin both. But this situation happens rarely. Illiterate persons are mainly doing this sometimes.

I: Do you provide any advice about sharing medicines with anyone else?

P: I always advise that ‘do not share the medicines with other members of the family or anyone else. Please complete your full course. Do not stop taking medicine in the middle of the course.’

I: Do you provide any advice about keeping leftover antibiotics if they didn’t complete the course?

P: I advises them to complete the course. No one admit that they share the leftover medicines to anyone.

I: Do you provide any advice about the correct dosage of medicines for how many days and how many times a day?

P: I explain about the doses of medicines and how to consume.

I: If the supply is inadequate, what do you tell the patients to do?

P: If the medicines are not available at community clinic, I refer the patients to the UHC and pharmacy to collect the medicines.

I: If patients do need antibiotics, then where do they get them from?

P: From Community clinic, people usually get 1^st^ generation medicines, that’s why sometimes they visit UHC to get 3^rd^ generation medicines. Antibiotics can be purchased easily from Pharmacy, if the name of the antibiotics can be mentioned. UHC sometimes referred patients to the pharmacy when they have inadequate medicines. Patients can buy medicines even without prescription. From Paramedics patients can get 3rd generation medicines, in that case patients get cured within 2 days. This doctor provide medicines without prescription when patients describes his or her health condition.

I: Can you tell me whether people can acquire antibiotics without a prescription and how easy or difficult that is?

P: Patients can get medicine easily from pharmacy and village doctor.

I: Is a prescription always required?

P: The pharmacy provides the antibiotics to the patients without prescription if the patient can mention the name of the antibiotic or the symptoms of the disease.

I: Can you tell me about how patients respond if you tell them that they do not need antibiotics, do they accept your explanation?

P: Most of the patients feel that CHCP is only for giving those medicines not for counselling. They need only medicines. I try to counsel them but if they do not understand sometimes, and send them to UHC. In some instances, they showed adverse attitude or got agitated.

I: Do they insist or become angry?

P: In my opinion, some patients would get angry/agitated, behave rudely. In some cases, I try to explain the reason to the person.

I: Do you think that they go somewhere else and, if so, where do you think they go?

P: When I refer.

I: Do you think they use leftovers medicines or share with other people?

P: Some of them shared leftover medicines with their family members. For example, one patient came for her coughing problem, I provided her capsule but she also wanted a medicine for her daughter too. Then I advised her to bring her daughter at Community clinic but the patient asked whether she can share her medicines with her daughter.

I: Do you think that patients do as you advise them to do? Why or why not?

P: As per my opinion maximum patients complete their courses because I instigates fear inside the patient.

I: Do patients keep medicines for later use (for themselves or others), do they share them?

P: Patients might share their medicines with other persons of their home if they (indicating other family members) get sick too.

I: Do you think they use leftovers?

P: I assume that there may be some patients who share the leftover medicines with their family members but patients never tell this to me.

I: Do you provide any advice about the correct dosage of medicines for how many days and how many times a day?

P: Yes, Patients follow my advice about doses of general medicines. But for antibiotics everyone does not follow my words because of lack of knowledge about antibiotics.

I: Have you ever heard the term “antibiotic resistance”? Can you tell me what you know?

P: I know about what is antibiotic resistance. It means deteriorating effectiveness of antibiotics.

When antibiotic fails on microbial efficacy on germs, it is called antibiotic resistance. I always try to explain the patients that how germs works and how antibiotic work over germs also.

**Part two : Potential Intervention**

I. Can you tell me what other work you do as a CHCP?

P: I usually deliver health education on the way to going home or on the way to coming back from Community clinic with the neibours or my other people.

I: Do you deliver any the court yard meeting or ‘uthan boithok’?

P: No generally when large number of patients come in the community clinic then we sit all together and discuss about some issues such as health related, social, community clinic related issues including drugs, early marriage, pregnant women, and vaccination.

I: What types of issues are discussed in meetings?

P: Given the distance from Daudkandi to community clinic, I advise pregnant women to shift to Daudkandi one and half months before her delivery. We discuss about hand washing before taking food, early marriage, drug use, social issues.

I. Tell me about the community group and community support group for this clinic and who belong the group?

P: Each community group contains 17 members and community support group contains 51 people. Imam, Union Porishad member, Teacher, Widow, Businessman, Social worker, Homemaker, Student, Landlord, Freedom Fighter, Retired person, Family Welfare Assistant are members of community group.

I: Who belong to the groups?

P: Generally Community group members and community support group members belong to the group.

I: How are they being selected?

P: At first I talk to the chairman about selecting community group members and prepare a list who would be eligible for this role and then sort it out. Community group members select community support group members through discussion with each other. When we were selecting community group members, we gave them some criteria, such as what type of member will be in this group, for example Imam, Freedom fighter, women etc, and these criteria had been given from Upazila Health Complex. Community Support Group members were selected in a similar way like community group members, such as teacher, social worker, and influential person from the area.

I: What are the regular activities of these groups?

P: Role of Community group members include checking cleanliness of community clinic, maintenance of community clinic. They discuss about some social issues such as early marriage, drugs abuse. In case of Community support groups, the meetings held with community groups with me is being discussed with CSG and they implement the discussion and decisions. I and CG members advise to CSG members to communicate the other locality of the area as much as possible as they can.

I: What if someone doesn’t want to belong anymore?

P: Then we select another person. But if someone is not interested or is not so active then CHCP reports to UHC. Then he or she might be changed after 1 year.

I: Who monitors the work of these groups?

P: Health inspector, medical officers from Upazila health complex monitor Community group and CHCP herself and support group members and sometimes Upazila health inspector monitor Community support groups.

I: What types of issues are discussed in these meetings?

P: Health related issues, social issues, community clinic related issues such as drugs, and early marriage, pregnant women, and vaccination- these are mainly discussed in the meetings. Sometimes we discuss about natural calamities and we also discuss how to create awareness on health issues among the people.

I: Who initiates the meetings?

P: I generally initiate these meetings.

I: Who is responsible for organizes them and mobilizes participants?

P: I generally organize and mobilize participants.

I: Who are the participants of these meetings?

P: Usually Community group members and community support group members join these meetings.

I: Are there separate meetings for male and female?

P: In general community group and support group member’s meetings are held with both Male – female. If any female related issue arises then only female participates participate the meetings with CHCP.

I: How often and how long do they occur?

P: With Community group member it will happen every 1 month. With Community Support group it is held in every 2 months and continues for one and half an hour at primary school which is beside the community clinic.

I: What motivates people to participate in these types of meetings?

P: Generally I motivate the people to join these meetings.

I: Can you tell me if there are any existing volunteers in this area who work to deliver health issues in the locality?

P: No, there is no existing volunteers in our locality.

I: What will be the criteria for selecting the volunteers?

P: If volunteer should be recruited they should be well educated, able to speak well. In addition, they must have time, who has experience of counselling, is an influential person, should be humble and well behaved and have the ability to explain health issues in a proper way. Powerful person such as Imam can facilitate through miking,can announce that ‘we can discuss some important issues’.

I: Is it useful to recruit both male and female volunteers?

P: Male and females can be selected but I personally feel that male has time to convince others and people can accept their advice soon.

I: Who will supervise the volunteers?

P: Me or Community group member, influential person can supervise them.

I: How long will they work on a week?

P: May be 2/3 hours is possible for volunteers to work in every week.

I: What will motivate volunteers to work?

P: If the volunteers are motivated and understand the issue, they could motivate the local people.

I: Can the volunteers be linked to the health system?

P: The volunteers can be linked by making the facilitators a part of the meeting, which is conducted among the Community Group and Community Support Group. He can also join the Dhaka meeting.

I: Do volunteers receive any kind of incentives for their work?

P: I have no idea about it.

I: Who might be able to keep simple records of the activities that take place at the regular meetings?

P: I can keep record myself first and send to the focal person who will monitor the volunteers.

I: Can you tell me how you think that you could be involved in this intervention?

P: I can be involved through giving them training and to make them understand the importance of the issue.

I: Who delivers health information of this area?

P: Family Welfare Assistant. High School teachers of this area also support through informing students about hand washing, vaccination, drugs.

I: What is the format in which the information is provided to the people?

P: Given through Poster, Hand Leaflet, verbally in the community clinic, Shonali Alo

I: What do you think- will people trust this delivering system of the information?

P: Yes, people will trust the information provided through poster and verbal information. Because the information is given by Government.

I: Do people prefer photos or drawings?

P: They prefer picture and verbal information together.
