# Supplementary material for: A process for developing a sustainable and scalable approach to community engagement: community dialogue approach for addressing the drivers of antibiotic resistance in Bangladesh
Source: BMC Public Health. 2020 Jun 17;20:950. doi: 10.1186/s12889-020-09033-5 (PMC7302129; doi:10.1186/s12889-020-09033-5)
Supplement: Supplementary file 15 — Additional file 15. CHCP CC5. Transcript of interview with community health care practitioner, region 5. [file 12889_2020_9033_MOESM15_ESM.docx]

| **Study Name:** **Community Dialogue for preventing and controlling antibiotic resistance in Bangladesh: Case for Support** | **Interview ID: CC5 CHCP** |
| --- | --- |
|  | **Date of Interview:**  **07/05/2017** |

I: Interviewer

P: Participants

I: Firstly I would like to know from you about the health seeking behavior of the people of your community. If they feel sick where do they usually go for treatment?

P: When the people of this community feel sick they come here first. I ask them that before coming here have they taken any medicine for sickness. They usually say no and also say that they have come to me for advice and treatment purpose. If I refuse them to give medicine then they will go to the doctor nearby. But they don’t go to the other place without my referral. If I can understand that the medicine I have won’t work for the disease, I tell them that and refer them.

I: Where do you refer them for better treatment?

P: I only refer to Upozila health complex, not anywhere else. I tell them to avoid other places because I am sending them to Upozila Health Complex where they will be treated by an MBBS doctor who is better than other health service providers.

I: Can you please tell me the other places where people use to go for treatment when they feel sick?

P: People of this area use to go to village doctor, to homeopath. It depends on their faith on the different providers. There is a Family welfare centre on the opposite direction of my clinic. People also go there and Upozila Health Complex too.

I: Is there any traditional healer in this area?

P: I think there are traditional healers but very few people go to them. Most of them come to me first for the treatment. No matter how serious the disease condition is, they will come to me first.

I: Why do people go to Family Welfare Centre?

P: I think it depends on the distance. The people nearer to Family Welfare Centre go there for treatment.

I: Is any paramedics practicing here?

P: We call the paramedic doctors as village doctors. I am not sure about the number. There might be one or two paramedics in this area.

I: Who goes to village doctors for treatment?

P: Transportation problem is the main problem in our area. It is very difficult to go to Iliyatgong by walking. For now they go to Kushiara.

I: What about pharmacy? Do people go to pharmacy for treatment and medicines?

P: I think a very few people go to the pharmacy. There are 2/3 doctors who are the owners of pharmacies.

I: What are the locations of pharmacies?

P: Pharmacies are in the bazaar. Kushiara .

I: Tell me about the health seeking behaviors of different people of different age groups especially pregnant women and children under 5yrs?

P: Pregnant women usually come to my clinic first for treatment and go to satellite clinic every month. Family Welfare Visitor comes here once a month, so pregnant women come here for monthly check up. The FWV do three to four satellite clinics in this area. I advise people to go to other satellite clinics if my one is far from there place.

I: Then what about the children? Where do the go?

P: In case of infant, they are taken to the upozila health complex. In some serious cases the children are also taken to the upozila health complex. Otherwise people come to my clinic for their child’s treatment too.

I: Why do people go to homeopath and traditional healers?... with what symptoms?

P: They take their children to the homeopath when they are suffering from common cold and fever. Women go to the homeopath for tumor or tonsillitis treatment. In these cases they have full faith on homeopathic medicines. They don’t want to follow our advice.

I: Do they go to the traditional healer for the same reason?

P: No. They don’t go to the traditional healer to seek treatment but there are some superstitions among the people about some spiritual matter. They go there to get relief from the problem.

I: What type of diseases is usually seen in your area? Please name some common diseases.

P: Common cold, cough, fever, allergy are common, so far I have seen and experienced here. Skin disease is a very common problem in this area because most of the people are related to pisciculture. So water of all the ponds is contaminated with various chemicals and organisms. They use that water for bathing and washing cloths and utensils. That is why lots of people suffer from skin disease throughout the year. Asthma, arthritis, diarrhea, typhoid, dysentery, hookworm infestation are also common among the people of the community.

I: Are you treating every disease or some specific diseases?

P: I give the general treatment of few diseases. When I realize that I am not able to treat the disease, I refer them to upozila health complex.

I: Is this ever happened that people came to your clinic for treatment and you diagnosed that they only need rest and no medicine is needed?

P: It happened several times. When I said them that they don’t need medicine, they got angry. They think that I have enough medicine but I don’t want to give them. Even some people come here for medicines to keep them at their home and store them. I just explain them the bad side of irrational use of medicines. Some people do agree with me and some don’t.

I: What type of medicines do you frequently prescribe? And what they want more?

P: Painkiller like paracetamol, iron, folic acid, anti ulcerant, cotrimoxazole, metronidazole are commonly prescribed drug. But they want Iron for weakness. Histacin and antacid are also given.

I: Tell me about the antibiotics. Which antibiotic do you give them most commonly?

P: I try not to give antibiotics. But I give amoxicillin and cotrimoxazole most commonly.

I: When do you prescribe antibiotics?

P: In case of infections, severe diarrhea, dysentery I prescribe antibiotics. But I don’t give medicines in case of pneumonia, typhoid, and paratyphoid. They require test before treatment.

I: Do you give them any advice while giving the antibiotics?

P: I tell them about the side effects of the antibiotic such as rash, itching. If these happen to them I advise them to stop taking the medicine. I also tell them to take plenty of water and take the antibiotic on time. The course must be completed. One can’t stop taking antibiotic in the middle of the course.

I: Do you tell them anything about sharing the antibiotic with others?

P: Of course not because I never give them extra medicines to share with. Other people may not have the same disease from which he/she is suffering. So he/she can’t do that. I never give them extra medicines.

I: Do you provide them full course of medicines?

P: If I have sufficient medicines in stock, I give them a full course of it. But if not then I give them antibiotics for 3 days and tell them to come for a follow up after three days and on that time I give them rest of the medicines of the course.

I: What do you tell them about the left over medicines?

P: There is no chance of having leftovers. But in case he or she has leftover medicines in their home, I tell them to bring them to me. I strictly forbid them to advice any of the medicine to anyone. They are told to bring the sick people to me and I will decide the treatment plan.

I: What do you do if you don’t have adequate stock of medicines? Or out of stock?

P: If I don’t have antibiotics at all then I refer them to upozila health complex when I realize that they really need antibiotics. I refer them but I don’t have any idea about where they go from here.

I: While referring the patient to upozila health complex do you write a prescription or a reference slip?

P: I am not authorized to write a prescription. I just tell them orally. And for their better understanding I cut the medicine blisters as per their daily dose.

I: If someone thinks that he/she needs antibiotics from where he/she can get it?

P: They can get antibiotics from pharmacies of village doctors, Upozila Health Complex, Family Welfare Centre. There are other pharmacies too.

I: Do they go to other providers to verify treatment and medicines you provided?

P: No, they come to me to verify other’s treatment or ask for the medicines they are prescribed.

I: If someone thinks that he is serious ill, he needs antibiotic but you have given only some advices such as takeing rest and eat healthy food then what does he do?

P: They think that I am storing medicines and intentionally not giving them medicines.

I: Who usually reacts more?

P: Women react more than men. Men are quite understanding in this matter.

I: You said that you don’t write prescription. So from where a person can get antibiotics without prescription?

P: If he says about his problems in FWC, he will get antibiotic according to his disease. From pharmacies too.

I: How does he get antibiotic from pharmacies?

P: If someone can tell the mane of the antibiotic, the pharmacist will give it.

I: What happens if he can’t recall the name of the medicine?

P: Then the pharmacist gives a medicine according to his problem.

I: Okay.. People get antibiotics from several sources without prescription but do other people give instructions and advices to them about taking antibiotics?

P: I don’t know. But I tell them.

I: Do you think people complete the course they are given?

P: Most of them do. I always tell them that if they stop taking antibiotic in the middle of the course, the organisms will not be killed. If they will take the same medicine for the same problem after some days, that antibiotic will not work.

I: What about the others? What do they do?

P: When few people feel better after taking 2/3 doses, they stop taking it. They keep rest for the medicines in home and throw away after the date expires. But when they get prescription from the other providers, they usually they 4/5 tablets or capsules initially, not the full course of antibiotic at a time.

I: Do you think people share the leftover antibiotics?

P: No, I don’t think so.

I: Did you ever heard about antibiotic resistant?

P: No.

Intervention part:

I: Please tell me about your job responsibility.

P: I usually come to the community clinic before 9am and after cleaning the clinic I start serving the patients till 3pm. I keep records in the register book.

I: Do you give any other health service to the people?

P: I give them health education when they come to my clinic.

I: Can you give me some example?

P: I advise them to stay clean, to wash their hands before eating, before feeding the child and after coming from toilet.

I: Is it happened ever that you have given these educations to many people at a time? I mean in a gathering?

P: Yes, when the members of community support group come here for the meeting I used to spread these messages among them.

I: Okay. Then you don’t conduct any uthan boithak?

P: No. my work is limited into this community clinic. But Health assistant and Family welfare assistant usually conduct Uthan boithak,

I: Would you please give me a brief about the community group and community support group of your clinic?

P: The community group and community support groups of this clinic are quite active. If I request them to come for meeting, most of them attend the meeting but few of them can’t come. Meeting and discussion sessions are held here with them. We all discuss about the development of this community clinic.

I: Who are in this these groups?

P:Union Parishad(UP) member, female UP members, landlord, freedom fighter, adolescent, handicapped, poor, businessmen, religious leader, teacher, jobholder and people from other categories.

I: Who select the CG and CSG members?

P: We received a list of categories from upozila health complex. According to that list we select the community and community support group members.

I: You said we. Who are the other people who are responsible for selecting the members?

P: FWA, HA, UP member and I together selected the members. Few other respectable people of the community also helped us in this job.

I: What are the major responsibilities of CG and CSG group?

P: Their responsibilities are to solve the problems those arise in the community clinics. Sometimes they help the patients. They monitor the activities of the CHCP. Some time they deliver the information about vaccination, health educations. They also monitor the unpacking of the medicines those come from Upozila Health Complex.

I: Are the all members active?

P: Not all of them but most of them are active.

I: If someone doesn’t want to be a part of the group anymore, what action will be taken to resolve the matter?

P: If someone wants to withdraw himself from the group, we replace him with someone else who is from the same category.

I: Who supervises the work of CG and CSG groups’ activity?

P: Those people who come from the Upozila Health Complex for inspection usually.

I: How CC, CG and CSG are linked with each other?

P: They are closely related to each other. They are always ready to solve any issue related to CC. They try their level best. Upozila Heath Complex is also related to us. They monitor and supervise the activity.

I: How they resolve the issues?

P: Through meetings. We all sit together and seek for the solutions.

I: Where do the meetings take place?

P: In my clinic.

I: How often these meetings take place?

P: Meetings with CG in every month and meetings with CSG in every two months.

I: You told me earlier that most of the members of the groups are active. Would you please tell me what motivate them to be active?

P: They are indirectly working for the wellbeing of the people of this community. They work actively for the development and improvement of the clinic and people are benefited from the clinic. This is the main satisfaction and motivation.

I: But they are working as a volunteer. Why?

P: They know that people are benefited by them.

I: Have they ever faced any problem doing their own duties?

P: There is no chance to face any difficulty.

I: Okay. How do the people of this community get the health education or health related information?

P: They get the education from FWA and HA in the EPI camps.

I: Is there any other source?

P: They get to know about health related issues from this community clinic, Family Welfare Centre and Upozila Heath Complex too.

I: Is there anyone who goes to every Bari to give health education?

P: FWA goes to every Bari for this purpose.

I: Is there any organization or individual who arrange uthan baithak?

P: There are BRAC and PUSTI projects here. I saw them arranging the uthan baithak. But I haven’t seen them recently.

I: Do you have any idea about the topics of those Uthan Baithak?

P: Yes, any type of health issues, problems, its solution. 10 people get to know about this and another 10 people will know from those 10 people who attended the meeting.

I: Can you tell me what type of health education is given there?

P: Eating healthy food, maintaining the hygiene, hookworm infestation and its cure are the common topics of those meetings.

I: who facilitate the meetings?

P: Health worker of BRAC and female health workers from PUSTI project facilitate the meetings.

I: Are there any volunteer working in your area?

P: Yes. There are volunteers.

I: Who are the participants of these meeting?

P: Male and female both. Pregnant women attend the meetings of PUSTI.

I: How often these meetings are arranged?

P: I don’t have idea about that.

I: Can you give us any idea about the time and duration of the meetings?

P: Usually these are held in the morning within 10:00am to 11:00am and the duration is one hour to one and a half hour.

I: Why do people attend this type of meetings?

P: Women attend these meetings because they bring up the children. So from this meeting they get to know many health related information which are very helpful in bringing up a child in proper and healthy way.

I: Have they faced any problem to attend these meetings? They may have work on that specific time?

P: No. They have been informed about the time and date of the meeting.

I: That’s great. Now I want suggestions from you about our project. [Explained the objectives of the project and shared the idea about facilitator/ volunteer]. Will it be possible if we want to recruit some volunteers/ facilitators for our project?

P: Yes possible. It will be very good if you can do this. Lots of people will show interest to help the community people. The areas where there is no HA, many people work there as volunteer.

I: How many people are working as volunteer at present?

P: 5 to6 people.

I: Is it necessary to recruit both male and female volunteer?

P: It is necessary. Male volunteer for male participants and female participants of female participants. Men may not take a female volunteer seriously.

I: Would you please tell me who are responsible for selecting the existing volunteers?

P: Those who supervise the activities of volunteer usually involve in the recruitment procedure.

I: Who supervise those volunteers?

P: Those organizations have their own staff for supervision. They come here frequently to supervise the volunteers work.

I: How the volunteers are linked with the health system?

P: They get the training according to a curriculum. So somehow they are linked with health system.

I: In your opinion what should be our way of selection of volunteers/ facilitators?

P: You must have a list of eligibility criteria. Select those who will meet the criteria.

I: What should be the qualities of a supervisor?

P: They should be educated and should have the capability to convince people and make them things understand easily.

I: How can we link our volunteers with your CC?

P: They will come to our area and work here, we will be introduced to them and they can share anything with us.

I: Will it be possible for you and CG, CSG members to supervise the work of the volunteer’s activity?

P: It won’t be possible for me because I have to work here from 9am to 3pm. But I don’t know about the group members. You can ask them. But I don’t think they will agree to do this because everyone has his own work.

I: Will you allow them to attend your meeting and report you about their activities?

P: Yes, this is possible. In our monthly meeting we can invite them to attend and share their experiences.

I: What can we do to motivate them? Why they will agree to work as a volunteer?

P: They will agree to work as a volunteer on their own interest.

I: What do you mean by own interest?

P: Remuneration… the existing volunteers are getting salary monthly and transportation fee too.

I: How many volunteer will be needed for your community?

P: Six thousand people are getting service from my clinic. You will need minimum six volunteers, three male and three female.

I: According to you monthly incentive motivates them. So how much money we have to pay?

P: As they will work all days in the month, so you have to pay 1500taka per month.

I: No no. they will work four days in a month and 3to 4 hours in a day or 4 days in a week and 1hour a day.

P: Oh okay.

I: We are at the end of the interview…Can you please tell me about the materials that the heath workers or volunteers use during meetings/Uthanbaihak

P: They have poster. They show poster, banner, and leaflet. They also discuss with the participants showing those materials.

I: Which materials do people like most?

P: They like pictures more. If I only explain them without showing anything then after some time they will forget what I said. But if I show pictures while explaining they will remember it for a long time.

I: Which type of picture they like? Photo or painting?

P: Photo.

I: Okay… Thank you so much for giving me you time.
