# Supplementary material for: A process for developing a sustainable and scalable approach to community engagement: community dialogue approach for addressing the drivers of antibiotic resistance in Bangladesh
Source: BMC Public Health. 2020 Jun 17;20:950. doi: 10.1186/s12889-020-09033-5 (PMC7302129; doi:10.1186/s12889-020-09033-5)
Supplement: Supplementary file 16 — Additional file 16. CHCP CC2. Transcript of interview with community health care practitioner, region 2 [file 12889_2020_9033_MOESM16_ESM.doc]

| **Study Name:** **Community Dialogue for preventing and controlling antibiotic resistance in Bangladesh: Case for Support** | **Interview ID: CC2 CHCP** |
| --- | --- |
|  | **Date of Interview:**  **11/04/2017** |

I: Interview

P: Participant

I: Have you understood the topics, we are going to discuss with you in the next?

P: Yes,

I: Please, would you tell us, when the people of this area get sick, then they do not feel well due to their ailments, then what do they do usually for their medication?

P: Usually then they visit the nearby community clinic or the Upazila Health complex.

I: What do you mean by nearby, would you please Specify?

P: Nearby means, we have now our area basis Community Center. They (patients) first come at this community Clinic. They reported to us first. Now they tell us their health problems. We listen first and then give them the medication or treatments.

I: In that Case, do you think the people of this area come in this clinic first.

P: They come at Shatpara Community Clinic.

I: Now please, tell me, why do they come here?

P: When they feel sick, then they come here.

I: Say- here is also the Upazila Health complex, is not it?

P: When the case is not manageable in this primary health care center, then we refer them to the UHC.

I: I wanted to know those reasons, why the people are coming here.

P: If the patient is manageable within the primary health care center, we treat them accordingly. But if it is out of our capacity then we cannot provide the treatment as the availability of drug is limited and then we refer them with a refer slip.

I: What do you mean by primary health care service?

P: Cold, cough, fever, and incase of acute, watery diarrhea, all these services are includes in primary health care. We provide treatment to the patients at the first stage and if fail in case of Complication-then refer them to............

I: To show them those, services are includes only?

P: To see and advise them. Give them healthcare education, then we check or examine the pregnant mothers with ANC, PNC, and check the increase of weight, height etc.

I: Please, tell me- what are the other services, you do offer to them from here?

P: We give them the medicine. We make them understand able about the treatment and cut the medicine strip in to pieces for their better dose schedule maintenance.

I: In this case, please - could you tell me, which group of people is coming here for frequent treatment? For example- Child, pregnant mothers, aged people or other?

P: Child patient frequency is more to visit here to take the treatment than the other areas. At the end of the month, almost 100 to 110 children get the services. Attendances of children patient are higher in comparison to others. General people and adolescent complain that they cannot eat the food or suffer in anorexia.

I: Who are those people most frequently visit here?

P: Adolescents, boys and girls, they don’t have the scope to visit the center due to schooling. Often they do send their mothers by telling them the health problems and we give them the treatment with medicine, if they can’t come. Most of the tine we inform them that the boy or girls should be sent to the center for proper medication.

I: In that case, who are coming here most?

P : It is often-mothers are mostly visited.

I: Is there any specific reason of coming female/male mostly?

P : Female patients come more than the male. Female are having problems with weakness, anorexia-these types of problems are more frequent. If they come to me with other problems but mentioned their problem as they cannot eat, physical weakness with headache. Specially when they stand up from their sitting position feel vertigo, which is the most common problem, they come with.

I: Now I just want to know from you that you already achieved an experience of giving medication or so far you being experienced in this regard, I would like to know about that experience. In that case, would you please, tell me, with what type of ailments, people are coming here mostly.

P : Most of them come here with pain problem, either waist or knee pain are common. Waist and knee pain are more frequent. But now a days, people are complaining, aged below 30 Yrs. of old, come here with frequent complain of pain and pain, I feel bad to listen such a complain of pain both from the boys and girls those who aged below 30 Yrs. People aged above 30 are having more frequent complain of pain. They use to come here with this type of problem more often.

I: Well then you are saying that people are coming to you for the treatment of pain problem. Please could you tell me for which ailments, you give them the medicine and for which ailments you don’t.

P : I give them the medicine, who comes here complaining pain with fever and the aged people suffered most in pain . Even than try to give them the medicine by checking their type of pain some of them suffer with knee pain and give them calcium and if other pain persist with fever , then I give them Napa or Paracetamol. Now we have seen that if someone continuously use this analgesic, which might harm the heart or Kidney. Then give them the health education and try to make them understand that excessive intake of analgesic may cause problem, we then even refer them to Govt. Medical Hospital for better treatment.

I: In the case of pain, you are telling that you give them the medicine after proper checking the cause of pain, But please, tell me a bit more about people, who are coming here with different type of ailments? Please tell me something about the diseases for which you give medicine to them and in which cases you don’t.

P: Cold, Cough, skin Diseases, In my area skin diseases are very high. The cause may be the high enough due to the ponds which are under fish cultivation. Most of the affected people come here with skin disease. And cold, cough is also common cause of ailments. I forbid them not to use the pond water because of problem and request them to use the tube well water. I distribute the medicine among them which is available here.

I: No Again-I Just want to know that you told most of the people come to you with skin disease, cold and caught?

P: Yes.

I : Do you give them medicines for two diseases?

P: If the people come with both, then I give them the medicine for both the diseases.

I: And what other case of diseases for which you usually do not give medicine but only advice.

P: Primarily, almost all the treatments we give to the patients.

I: Well you can not tell.

P : No-I understand the meaning at first the patents told that he is suffering in fever or cold, then I try to gauge the fever with my hand by touching his body and if I feel, it is more than 98^0^ F, then I give them the medicine. If it is not exceed 98^0^ F, I do not give medicine but advise them, when the body temperature is increased, then I give, them the medicine, I advise them in the next, If the body temperature raised more than 98^0^ F, tell them to come at the center and them I give them the medicine.

I: So-we see, people are suffering from cold, cough, fever and then skin diseases. And in case of diarrhoel disease, you are also giving them the medicine. You have mentioned meanwhile that if the body temperature remains below 98^0^ F, you do not give them the medicine. In such a case, what are those diseases you do not give them the medicine, please tell us something about.

P : It may be the antibiotic, example in most cases, People come to me with the complain of cold and cough and often they themselves like to play the role of a doctor. Then I try to help them to understand about the medicine antibiotic of cold and cough. If the Problem is severe then I give them the normal medication and after giving three days if the patient is not improve. If the patient feels better within three days, then it is ok and if it is not, I tell them to come after three days. Then I checked him again and give him the 7 days antibiotic course by cutting the strip to case the dose schedule.

I : The medication you have given is all right and we need that information .Is it happen that when people come here with diseases to you for treatment but you think , it may not necessary to give them the medicine , And you do not give him the medicine but tell him to leave after giving him some advice only . Are there any people come here for treatment with such ailments? What could be the type of medicine, you give them? Please, Could you tell me some name of the medicine; otherwise we will ask you the next question.

P: Those people come to this community clinic with diseases for treatment, I think that they come here for primary healthcare only. Say – they told me a single health problem which may not to give any medicine but advice. Then they bring another health issue to me. For example: I told that you need not to take any medicine for this problem, and then they bring another issue. In that case – so far I have been experienced, there no alternative than to give them some medicine.

I: So – you told that advice may be enough to them in some cases. What are those diseases?

P: Think that some younger people come here almost equal of my age and complain of pain. Then I tell them pain is not the problem. You shall have to keep on moving properly. Try to swim and then it would be all right. I can tell you from my experience that they do not follow the instructions. And say Madam – Please, give some medicine. Disturb me again and again. Sometime – I give them the medicine and sometime the advice only . All these are the stories of the services.

I: Well. Then mostly pain is the concern.

P: Yes.

I: So, then we can see two types of services are being offered here in case of pain management. Sometime you are giving medicine and sometime advice. What do you do in case of fever ?

P: Sometime , weak patient come and complain about their generalized weakness . Many of them take birth control pill i.e adopting family planning method . Those women take pill, they are usually suffer in weakness and I give them advice only .Sometime I tell them due to taking pill , you may feel weak .Eor this weakness, needs no medicine . Take or eat improved and healthy diet such as egg , milk etc then it would be all right , just advise them .

I: Many thanks to you for giving us the fruitful information . Now I want to know from you something about antibiotics . Meanwhile you informed us something about it . Eventhen I need to know a bit more information about antibiotic . When and how often , do you use the antibiotic for the patients ? So when and whom you give this medicine.

P: I do not give it always to every all patients . In most cases , first I ask them the cause of coming here .If they reply for cold and cough , then I ask them , how many days , they are suffering in it . If the reply is from today, I give them the normal medicine such as paracetamol and histacine .Those who come here with the complain of cold and cough within one or two days . Then I give them the normal medicine. Eventhen I told them to come again after three days. When they come after three days, then I check them again whether they cured or still suffering . If not cured – then I give them the antibiotics with 7 days dose schedule .

I: In which case of ailments , you give them the antibiotics ?

P: Mostly I give them the medicine in case of cold and cough and also some with cut injury patients . I give the cut injury patients from the very beginning . If it is any bigger cut injury , then for quick recovery .Then it is necessary to treat with the antibiotics .

I: In that case , have you ever been observed or known to you , what type of patients or age groups are mostly getting the antibiotics ?

P: If the patient is above 18 yrs. of old needs antibiotic and below may not be needed . If the patient is a mother and above 18 , may get the medication . Married father may also get the medication . But when they use to go for work, then the various problems may raise . And those mothers aged above 18 yrs. might have some more problems due to household work . Then the antibiotics work well . For this reason , We give it . Use of antibiotic is lesser for those below 18 yrs. of old . Except cut injury , needs not necessary ?

I: I want to know something more that you are giving the antibiotic as per the requirement to the patient and what else , you tell them ? For example: you told early that you give them 3 days medicine and tell them to come again and also advice them In case of giving antibiotic, what else you tell them ?

P: I tell them to take the medicine regularly and timely and if do not maintain the dose schedule, you may not get cured. So you may suffer in the next again. For this reason, you need to complete the 7 days antibiotic course otherwise you will not fully cure. Perhaps the intensity of the ailment may be reduced within 3 days but not will be cured completely. So – after 3 days, you will get some good effect but continuation of 7 days will give you relief from the disease .

I: Do you have any idea about the share of antibiotic ? Say- I have some antibiotic for my use but someone else gets sick meanwhile .Now I have seen that the disease , I am being suffering is as same as the another one is suffering . And then I share some medicine with him. I am telling you about antibiotic. So, in this case ,do you suggest them not to share it with others or you allow it ?

P: No, by no means , it can be shared with others because I forbid him. Certainly I tell it . There are some patients those who take medicine from me 2 days before but further again visit in the next with the complain of cold and fever . Because all the people are known to me of this community and then I asked her/ him , you took the medicine 2 days before and then she replied – Madam , my husband has taken that medicine . Then I tell her , why your husband will take that medicine ? I have given you the medicine for your treatment . Why you allow your husband to use it ? Then how you will be cured ? You will send your husband to me . I use to do lots of arguments with them in this regard .

I: In that case , did you tell her anything when you give her the medicine ?

P: Then and there , I told them while I give them the medicine whether it is for 3 days or 7 days course, need to be completed.

I: Did you tell them , the given medicine should not to be shared with the husband or others ?

P: Of course – I tell them . But they do not follow my instructions .

I: Well – that was the subject of sharing . Now if I tell you that he/she took the medicine from you as it a 7 days course to complete but it is seen that the patient has taken the medication for 3-4 days and then stopped. But he/she took the medicine from you . He / she keeps the rest medicine . What should be done in such a situation ? Should it be keep in this way or should not to be ? Should it be shared with others or not to be ? In such a case , do you give them any advice in this regard ?

P: When they come to take the medicine from me , I always give them this advice .Then I also give them the advice that the medication course should be completed and it is forbidden to share with others .

I: And what about the medicine that left unused ?

P: What would be left ? I am sure that I am giving them the 7 days medication course . They need to complete the course . If they do not complete the course , the disease will not cure . I am remainding them . So – there is no any chance to left the medicine .

I: Well. Perhaps you do not take it in that way of real sense in this regard .

P: Which way ?

I: I mean , it is all right about the given medicine but they say , they will use it . Now it is seen that he/she may not come to you for any reason but rest of that medicine remains to him/her . But you may not tell him/her anything about the use of that medicine .

P : No .

I : Well . It is all right . Thanks .

But, say – if you tell them that yes – you have here the full course of drugs.Do you think that eventhen they will move to some other places ?

P: Then they will not go . When I do refer them to there, only then, they go .But it is seen also that some people has gone there . No – but but it is seen that some people go in other places .Then later come to me and reported that their ailment is not cured. I told them that where have you gone ? Thet replied with the name of the certain places.

I: What is that certain place , you mean /

P: Some type of pharmacies .They Started the practice after completion of 3 months of medical course.

I: But – where have you sent them ?

P: I send them Upazila Health Complex.

I: Are there some patient like them ?

P: Yes. Usually – I get some this type of patients . They again come to me in the next And I ask them later, is the child yet not cured. They replied- no. I ask them. Why the child is not cured ? When have you gone there . Don`t you visit the UHC ? Then replied – no. We have gone at Shahidnagar and consulted with that certain Doctor .Then I tell them some for their goodness.And lamented- if you don`t go to the right place and your sufferings will increase. Then they alert about it .

I: From your information , I have understood that you told them to come to you after 2 or3 days later to take the medicine .But do they follow your instructions ?

P: yes – they do .

I: Is it happened that they do not come at times ?

P: Usually – It is not happen because there is the only Community Clinic in this area .

I: You are but advicing them that the drugs should be continued till 7 days. He/ She took the medication for 2 days .You told him/her to come after 2 days. But now he/she gets cure within 2 days medication.Have you seen such an incident that then he/she is not coming ?

P: No, no.- come. The community clinic is very nearby . And mostly we provide services to the women . Everybody come here first. If they wish , they can visit 10 times . There is no problem.

I: So- we have come to know that if here you have no antibiotic or short suppy of drug is reported, then you send them to Upazila Health Complex.

I: You have told me another information early that sometime such a thing happen that patients are telling about their ailment and you have given them the advice only .

I: You have understood that he/she needs no drugs and then he/she is telling you another ailment to take the drugs.

P: What you are saying is but very realistic .But it may be consider as the personal matter.

I : Okay. Understand .He/She has taken the drugs by telling of another ailment. Say – have you experienced such an incident that he/she complained to you about a certain disease and you have seen, no drugs or antibiotic is needed at all .But eventhen he/she is telling you for another health problem or this disease , he/she is forcing you to give him/ her the drugs .Have you face such type of problems?

P: Say – about the force means –

I: When someone comes to you for certain disease and forcing you with the demand to give certain drugs , I mean such thing.

P: Yes- I face such incident .

I: And what do you do in this situation even after your advice ?

P: Then he/she tells me that they also know some advice. Then I told that continuation or excessive use of this type of drug will harm you. It means. I tell and cautious them not to use so much of drugs. Some patients do agree and some are very egotic type and don`t listen or care to take the drugs .

I: In that case , what do you do , when they don`t care ?

P: when- I see the weakness problem. Then I consider and if not, try to make them understand .

I; I could not understand the weakness problem .

P: There are some female patient who complain about their weakness problem . Now you think, Someone comes with pain problem. Then I forbid them not to take the drugs and checked whether any other problems are having or not ?

I: You have said early that the term method ? What is that ?

P: Yes – right , I mean family planning services .I told that also about the pain problem . I told them for this type of pain , needs not to take any medicine .You will walk a while or exercise, then you will get relief .Then they told that no – you will give me some other drugs . If I ask them the real problem, then said – weakness or feeling bad. Then I give them some ORS.

I: In that case , those who wants only, you are giving them the ORS .

I; Please, tell me the real picture or scenario which is happened here very often ?

P: No, no .It is happened at times ., that`s all. AS I am working in this area , it is usal to give them this facility . It means, the medication, I offer to them which do not bring any harm to them.

I ; In that case , is he/ she understand it?

P: That is the drugs such as B-complex,ORS, Folic acid – I use to give them which is not harmful.

I: Does he/she know that the drugs are given to them is not antibiotic ?

P: Yes- Knows . I already mention it to them. And now-a days , most of the patients of this area know well what is antibiotic. They often request me, Madam – please, give some antibiotics.I ask them , why you will take the antibiotic drugs. What is the problem ? Then they replied that cold and cough problem. I ask them , how many days you are suffering ? Replied – from today . Is it necessary to take antibiotic, if the cough is being started today ? All right – these are the drugs given to you- take it properly . If then you are not cured within 3 days, visit me again . I shall talk with you after that 3 days .

I: So- in this case, you told that some patients told you about the antibiotic. Do they want it and specified by names of the antibiotics which they intend to get .Please- mention names ?

P: They can tell the name such as Amoxicillin which is available here . They can`t tell the name of other antibiotics so much .

I: Do you know well about amoxicillin ?

P: Yes .

I : So – naturally , they mention the name of the Amoxicillin.And do they mention the name of other antibiotic or know only Amoxicillin ?

P: No, no – mention antibiotic drug .They say- Madam, give me antibiotic if available . Do not mention the particular name . I always try to take the last case history.How many days the problems exist or which drugs they used during the treatment from the practioner . After knowing all these things- I try to select the drugs- that`s all? Many patients are under treatment and taking drugs in home, Even then , they come here for drugs . Then I give them some advice . The drugs, you have in your home which is given by your Doctor for the treatment should continue If your health condition is not improved, then you will contact me .

I: Now – I want to know further a bit more .

P: Please, tell me , what you want to know. I can remember some patients come to me with fever . Then I asked , when you are suffering from fever? Replied that from yesterday . Then I ask, are you taking any drugs and from where and which Doctor you have consulted ?Then they say , yes, Madam- I have brought the drug from the dispensary . Then I ask ,why you have come here ? First take that drugs. Then they take the drug . I tell them also if you are not cured after taking that drugs, then you will come here . I tell them the words in this way .

I: Then tell me something from the early discussion . You told me that you leave some of the patients after giving advice. Then after your advice, what they use to do ? Do you think , they go som where else for drugs because they don`t get any medicine from you. Are they gone some other places or they do follow your instructions ?

P: No,no. They use to listen my words or advice .

I: Well.You told that someone may need not to take the drug for specific ailment. Do you believe in this concept ? Say – you did not give him the medicine. But he/she could bring the drugs from the pharmacy or other places without informing you .

P: No, not at all . They did it themselves . They know that taking wrong drugs could bring harm . For this reason, what I told or advice, they follow .

I: Well. Another fact is that some people try to force you to give them drugs . Who are those people and what age group , mostly you see .

P: Think that the age group in between 30-40 yrs , mostly involved in it .

I: Are they male or female ?

P; Mostly female .Those are male don`t do it .The male feel shy to do it .Most of the male patients keep on stand at the backside .First – I give them the medicine . The serial of female patients is considerably more .

I: Say- apart from all discussion, I want to ask you a easy question.We have been discussed so many things so far .Now tell me specifically, you send people to Upazila Health Complex for antibiotic .

P: If not available here , then I send .

I: But they are getting some antibiotic from here . So- we talked about two different sources . Do you think , they or the people of this area may get the antibiotic and from which source or places , they may collect the antibiotic from this area ?

P: Can`t get .

I: Can get it .

I: The people may get the antibiotic from the place or source may be the pharmacy , as you informed me few minutes ago.

P: Yes. There are some pharmacies .

I: What are the other source or places , from where they may bring the antibiotic.

P: No. There are some other pharmacies in front of the road side by which way you reached here .

I: What is there .

P: They buy the drugs from that pharmacy as per their wish or requirement.

I: In that case , do they need any prescription to buy or bring the antibiotic ?

P: No, I have seen. I asked them, what are those drugs, you have been given. Later they bring it from the home. But there is no prescription. Then I told them, you should not be treated in this way. Always I use to forbid them once and again not to take treatment from outside. But you don’t pay heed my words. After forbid them once and again, they don’t feel to like give its importance. You are taking medicine from the quake doctor from the village without consultation with Govt. Hospital’s physician.

I: Those drugs, they bring to you without prescription, in that case do they tell you from their memory. Do they tell you any name of the antibiotic.

P: They can not tell the name. They are the Simple and general people of the villages. And those, who can tell the name, they will not go to the Pharmacy.

I: What they say?

P: They just go to the pharmacy and telling them their various health problems such as cold, cough, watery diarrhoea, dyscentry, fever etc, and then the pharmacist give them the drugs. They get the drugs. which is available there. Because you see - the Gouripur Pharmacy, they keep all the drugs. But in local Village area, not so much drugs are available. They do business in remote village area with low capital. Those drugs are available there, usually treated the patients in this area.

I: Then tell me some more information which I want to know that you told , they follow your advice or words of this area. Now if I ask you, Why the people are listeing your advice or instruction about their health problems.

P: They said that we are at least completed 3 months of education course. After completing the course, I get the job here. They know that we are the doctor for the primary health care services of this clinic. We offer primary health Services for this reason, they do think, we are certainly doctor, so they have to listen our words or advice for this reason, they follow or obey our words. And I am also from this locality as a result whatever I say to the patients, they obey. Specially we will tell them the advice for their betterment.

I: In this case, do you think, most of the people listen to you?

P: Most of them listen. But some patient may not follow my words and bring drugs from the pharmacy. But when they are not cured, then contact with me again.

I: Why they do not listen? You have told that few patients do not listen your words, why it is so happened?

P: They use to go there without visiting me. If they could meet me, it may then possible to tell them don’t go to that particular place for medication. If they go there without informing me, then I have nothing to do.

I: Here could you differentiate the particular age group-those who listen your words and those who don’t?

P: No, no. There is no differentiate in age groups. But the number is lower and some people will go. It is also happened in case of sudden ailment and in that case they go in other place. It is also seen that my office hour is started from 9 am to 3 pm in this community clinic. But after 3 pm, if any health problem raise in this area, they usually do not get me available in office, then they shift to go in other places.

I: well: Madam- tell me something which we discussed about the antibiotic in details. you are informing me many things which is really necessary for me to know. It is also necessary for you too. Have you ever heard about antibiotic resistance ? Or you know it from some where or somebody who informed you in any way?

P: No. I think know nobody talks about it.

I: And have you ever heard about it/

P: Yes, I heard it but none told me.

I: well, If you have heard about it, then could you tell me, something about it?

P: It might be something about preventive diseases.

I: Exactly, What It might be? Can you tell me about it.

P: No. If you could explain. It could be helpful for me.

I: Well. have you ever heard like you have just told me, some cases, say for example, one take the medication for 3 days and stop taking medication for the next 3 days. Then after few days the patient feel unwell and then come to me with a complain of ailment.

I: Again the patient come back to you. Then what happen after coming again, that’s I ask you? Then his ailment recur as due not to completing the full course of the drugs. In that case, generally the bacteria exist in his system in latent condition. As the patient did not complete the full course, The bacteria was exist in a latent stage by changing his condition and adapt with the drug taken by patient in system. As the bacteria adapt with the drug and recur the disease again. Then when you again, give him the same drug with 7 days course to complete, then the drug does not work, have you understood ? what is suppose to do in the next, need to increase the power of the drug. Or than need to use other group of drugs. That is the drug, given at the first time is no more working now.

P: Really- I could not understand it. But we use the power of antibiotic which is available here is 250mg antibiotic.

I: That is we call that drug antibiotic. In this way, we know about antibiotic resistance. You think that the drug, you first given to them for 7 days with certain strength. But when the disease recur again in the next, then you treated with the same drug does not work any more.

P: You are right and the drug does not work then.

I: That is why, it is called antibiotic resistance.

P: Yes.

I: The drug is but antibiotic.

P: I understand as you tell it in Bangla.

I: Am I tell you in English.

P: No. Still – I could not understand the main topic.

I: O.K. Good. Now I just want to know from you that do you think, the people of this villages know it?

P: Yes. They know.

I: Do they know about the antibiotic resistance?

P: No, no. It is not known. But I tell them that if the disease is not cured you will com to me again. And it is seen that we give them the drug named by cotrimoxazol 960mg.

I: What have you said that ?

P: Cotrim, Cotrimoxazol which is in our given drug list.

P: Please, see it is here.

I: Well. It is cotrimoxazol, Good. What else, you want to say?

P: No. we give it . Then you say about to increase power ?

I: Well. Good. Here you have 120mg and 960mg antibiotic, O.K.

I: Now we have finished the discussion on antibiotic so far. what we will do now, I have some question again. In this case, we shall have to know your Idea , what would be the benefit of it, is that the job we will perform and in that case, we will utilize your idea. Well. In this case, we will do some work with some important information, which will be needed for you. For example-so far, you have been shared your experience and knowledge with us about antibiotic which is very beneficial to us all. And you tell me something about –

I: Do you provide any healthcare services else where then this community clinic?

P: Yes. I provide services out side of this clinic also.

I: How do you provide that services?

P: Say-as I am now staying near my own house. When I stay home, then many people come to me and say-they come with various health problems. Then I give them advice. As you are telling outside of my clinic works.

I: What is this advice ? what topics are the subject of this advice?

P: Say – the advice is on health related such as acute watery feces or diarrhoea etc. Perhaps these types of services .

I: Have you done it at your home ?

P: Yeas- it is usually in my home but may also on the way, when they call me for help.

I: At home or when do they request on the way to go their home.

P: Yes - I get such a request from many patients.

I: Do you perform it at their home?

P: Yes- often use to go their home ? Many patients are staying around my clinic while I stay here. One day, a patient suddenly fall down with vertigo, due to high blood Pressure. Then we have rushed their with BP machine and measure the BP but it was O.K. The problem was perhaps due to weakness. Then I advise him to take nutritious diet and ORS should be continued-I advised.

I: In that case , you are telling that sometime means after your official duty of this CC. You use to do that jobs on the way to go home. You have told me that you give them lots of advice and even you sometime arrange satellite sitting for advise them. You do it by calling some people of that area.

P: We do it inside the home with mothers.

I: But do you do it outside or someone’s home outside?

P: No. It is not done.

I: Well.

P: We stay at the clinic till 3pm and then we go home. For this reason – we can’t. But on the way, patient problem may be solved by giving advice. That is I do.

I: Well. Now- will you tell me something about this Community Clinic and provide us some information about the community group and community support group of this clinic. There is a support group and community group in this clinic and need to no some information about it. For example: Who are those people included in this groups.

P: First there is a community group in this community clinic local UP member is our chair of this group.

I: Is it included in community group?

P: Yes-in community group. And also Up member and the president of this clinic. That is our female Up member is also with us. 2^nd^ member is the owner of this land, who is the Vice president and 4^th^ member is the treasurer, who is a freedom fighter and a retired govt. Officer, 5^th^ member is the ACHCP, who is the secretary and then include to other teachers. One of them is male teacher and another one is female teacher. And then two other social worker and both of them are male. And then there is a health assistant ms shahina is working as a volunteer worker. She is also in our support group.

I: Is she in both support group and community group ?

P: Her name is not included in community group.

I: In support group only ?

P: First she was included but later excluded as she withdraw her name from the volunteer service. Now Ms Taslima is working as health assistant in our 3 no. ward. It has recommended buy our office to give the name to us. Taslima is in support group. But their is no health assistant in our ward.

I: In community group ?

P: Yes. We do not have health assistant in our ward . So we have proposed her name from 3 no. ward, Who is working here now.

I: And who else are working ?

P: And another widow is working. There are some adolescence and then family planning worker FWA is also include in that group.

I: How many in total they are ?

P: 17 persons.

I: And who are those in community support group?

P: The same number are also included in this community support group category?

I: The members of this category?

P: Yes. But their are some exceptional case in support group? say-we are the member secretary. I am working 4 no. and 5 no. positions. we have taken one member from community group to support the group. She leads the tem. 3 groups are leading separate 3 groups. we made 3 community groups in this area. We have given 3 female members name from this community group.

I: Well. How many community support group, You told?

P: Three

I: Three in number at Community support group.

P: In total we form it with 51 members.

I: And how many members are taken from community group to community support group.

P: Three people. Three members in three group.

I: Is there any rule that how many member should be included or how many member could be given in community support group?

P: It is advised that we shall have to form the community support group with 17 persons.

I: How many people are included from the community group.

P: They advised to take 3 persons.

I: Not more than that?

P: No. no. Because the member secretary will lead the group.

I: Who tell you that 3 persons will be taken.

I: We have contact with the office about that 3 persons. we have got a form from the office. It is written below the form about three persons.

I: Which office?

P: Upazila Heath complex.

I: Well. You tell me that the people those are included in the various category and how do they elected in that position.

P: That is first – I made the community group. Then I formed if myself, I have visited the area to select the people those who are eager to work and also having dignity in the area. Those who are eager to give services as volunteer worker. I selected them, those who are having leisure time. At first, I made the support group, with 17 members. Before that, than I called the president, vice president, treasurer and other members including me of that support group and informed them to make the support group. And the group would be formed with 17 members candidates and distributed the post among them. I form it with their help in my area.

The support group we formed in my area which includes two village. here we have one member from Sholopara, and three members form my ward. Among the three members, Two members are working in both the groups. And the other member is in the community group. That much, I done. But I contact with that member and also visited his area. And I have collected more 17 members form that area. Again a team has been formed in that area apart from this area. In this way – I made the three groups.

I: But mainly when you form the community group with the Up member Shaheb and who else the other one you mentioned?

P: He is the treasurer.

I: Who is that treasurer. Is he the land contributor ?

P: No, no. Vice president is the land contributor.

I: And who is the treasurer?

P: He, treasurer comes from the freedom fighter kota, that is all.

I: Well. Then they include the land contributor, member , treasurer and you, who helps to formed the support group by election and give them various advice.

P: Those who are having interest to provide services in this group, whom we know as they are from our locality. I use to visit their homes and talked and advice to join in this support group.

I: You have given me some very good information. They have been elected by you and now so far 4 groups are formed but what type of jobs, they usually perform in community clinic in this area.

P: We request them to attend the monthly meeting and they participated. And we discussed the current issues or problems as for example: We discussed in the last meeting about the electricity problem. We are discussing from the very beginning about the connection of electricity in the clinic. Now – We approved it in the last meeting . The meter will we come soon but the electrical wiring work yet to be done. You see, in this way , we call the meeting each month with a view to discuss about health related problem such as diarrhea, dysentery, watery feces. In each meeting , we discuss with any one dieses or the problems , we face on the spot come one priority basis . They use to join in various occasion in their local area of the villages . There they can talk about the cause of diarrhea. We observe that cleanliness and personal hygiene is very much needed to avoid the above mentioned diseases. Specially nail cut and hand wash before taking the meal, We shall advice in every houses. And later advice, all the people of this area come to know all these health education.

I: You see – you formed 3-4 community support group but what are other jobs they use to perform apart from health services ?

P: Say- I requested the Up member to fill up the low land area in front of the clinic. We all requested the Union Parishad to allocate some fund for our development work. They may allocate some rice or wheat which we can sale to raise fund for development work for the clinic.

I: What I have understood that you certainly called them for meeting. You usually discuss the various issues with them including health services and development work. Generally they use to do all these jobs for the community group. And what else, they use to do.

P: Specially they perform these types of jobs. They verify the drugs , we distributed it among the patients. When we bring the listed allocated drugs from the Upzila Health complex, then they also present and verify the drugs with signature .

I: Do they supervise the drugs you received.

P: Yes- They supervise the amount of listed drugs and whether we distribute it to patients properly. How we behave or react with the patients , that’s also they observed.

I: We need to listen all these things from you which you mentioned at last about their types of supervision.

P: Sometimes, when the crowd of patients are in very excessive amount, then our temper can not be checked often and the patients complaint it to them that Madam- behave bad. Then I ask him, who told you and give his name . Then I tell them , all right ,what was the cause of that bad behavior and who complain it. I often feel bad with all these type of incidents.

I: Where does the meeting held?

P: In our community clinic.

I: How long time does it continues?

P: Mostly it continuous up to one hour. We can not take more time as most of them are busy people.

I: How many times , does it held in a month.

P: Community group meeting held once in a month and support group meeting held twice in a month.

I: Is it that three support group meeting which held together.

P: Yes- that three together. When we invite to attend, they all come but I see not that all members are attend the meeting.

I: You invite all that three groups twice in a month.

P: Those who are present here in the meeting , we discuss and advice various topics.

I: And community group meeting once in a month .

P: Yes.

I: Now you tell me whether have you seen that some of them are very good member and active , some of them are not so much active and some of them do not work at all.

P: Yes, you said the truth. They all do not work is not true but try and due to poverty, I utilize them in some works. Those who are poor, I involve them to work in cleanliness.

I: Say- Is it happened that a member comes to you and inform that he or she does not want to stay any more in the group?

P: None of them express such a view. They always feel that it is good enough to be a member of a Govt. organization. Primarily they do think it. They are very much interested to work here.

I: Usually you elected the groups. You formed three groups. What are the duration of these groups.

P: It has been changed in each year.

I: After how many years, the new groups formed?

P: We formed the group after one or two year. But we observe that if any member is not eager to work or do not feel like to come in time or continue or even after calling them over cell phone, they don’t response. Then after the discussion, we terminated them by saying that the office has issued this order. We take the final decision after consulting with the president.

I: You told me that they terminated them from the job but who instructed you to do it.

P: It is instructed from the Upazila Health complex office. I informed them, that all. those who are not interested to work voluntarily in this community clinic or those who do not come regularly, that types of member face termination.

I: I have a question here. You told me that those who are not eager or often absent in work, then the Upazila Health complex office terminate them. But how does the Upazila Health complex office get this information that certain members are not eager to work or absent in clinic.

P: They do not get that information.

I: Then how does the work being supervised and who use to do it?

P: No, no. It is me who is justified it as a member secretary of this clinic. I lead all the function here. Now I can see the assigned jobs are not being properly done by certain member, when I call them for the work but don’t listen my words. Then I dissatisfied.

I: Do you report them?

P: Yes. Then they (UHC) suggested me to change the person after one or two years, when we change the group.

I: O: K. I understand. What else happen?

P: For example often such a thing has happened in each month that Headmaster of Boropara school is a teacher named by Mr. Alam is also a member but when I invite him , he kept busy with his own teaching business and can not attend at the clinic , then we decided to change him. A school teacher of a Kindergarden school, who shows his keen interest to work with us and then I nominated his name.

I: Now, I want to know that as you have said when any worker do not work, you take action against them. Now you have formed the community group and the community support group, those who are working here in many ways and other members are also working simultaneously. As you mentioned about group meeting and other works, who supervise jobs whether they are working it properly or not? Yes- you have formed the community support group but whether they are working properly or not, who usually supervise their activities?

P: It is supervised by the CHCP.

I: Usually, the CHCP look after it. But in fact, you look after their activities whether they are working properly or not.

P: No, no. Sorry, I look after the activities of the community group only. And the member of the community group looks after the activities of the community support group whether they are working properly or not.

I: Well. That is I wanted to know that you have formed three member community support group those who look after the activities of the community support group. And you look after the activities of the community group.

P: Yes.

I: O.K. good.

P: Do you think as it your opinion that the activities of the members of the community group or community support group are nearly up to the mark or not. How much they support your community clinic in respect to health, work and development .

I: Yes, they do as much as they can. The electricity connection in this clinic is their contribution and effort.

I: As your opinion , it means, they are very active.

P: Yes.

I: And I have also come to know the fact.

I: I shall bring the wiring line and fan inside the clinic with their help, which I already told, you see, some people contribute various helps in each year in the name Almighty Allah. I told them to give them contribution to this clinic so if we can collect some money in this way , then we deposit it as our fund in bank account.

I: Now you please tell me, as you mentioned that the members of the community group on the community support group are very hardy and active, why do you think so, please tell the reasons.

P: Whenever- I call them, get them available. And sometime patients come to us for drugs and creates trouble for taking more drugs, Then we told them the regulation of taking medicine for 3 days . But when they demand more drugs, we take help from our supporting members who make them calm.

I: Whom you call?

P: One or two members of the community group. we call.

I: To whom they counsel?

P: They told the patents that it is the governments rule to give the drugs for 3 days course. If then, you are not cured, you will get the drugs for more days. First you take the 3 days medication. Sometime, we face such situation,

I: Now I want to know about the responsibilities of the various community group for the three community support groups, those you have given the various responsibilities to perform their duties, do they ever face then any problems? Say- they have been assigned to do their duties in community clinic or some other things or health service about the people of that particular area. As you then present there, have you seen any problems , they do face?

P: No, They do not face any problem and those members are working here are respected people in this area. The people of this area obey them well.

I: It I do not misunderstand you that to see the problem as you are same, when a patient demanding more drugs once and again, then you call the members in this case to resolve the problem . It is but a duty of this clinic. But in case of community clinic and if we think the development or the healthcare service of the people of this community or the responsibilities , you given to them after you elected them , do you think, they face any challenges ? Do they think the given task is hard enough or problematic or they face any real challenges?

P: No. They do not face such a challenge here so far.

I: Well, is it even happen in general- say, when you move to solve a work or problem but which is not yet faced properly but you want to go ahead with that work further more and will have to be done. How do they face to solve the problem. Do you able to further proceed ? How do you perform the work with the help of the community group or the community support group, Am I clear to you about my question?

P: Yes. Understand. We never face such a problem as you mentioning .For this reason, it is not possible to answer your query.

I: Say for an example, you have informed that you are having trouble for the electricity supply in this clinic. Well – as you have said , you don’t have electricity here which is a vital problem . So , in that case , how do you solve the problem ?

P: I called the members and requested them to arrange to bring the electric line here and we applied for it . Then we have got electricity line within two months time.

I: How do you perform this jobs? It was a problem , but how did you solve the problem ?

P: When I called them , they come and some one has applied for it, whom we selected to perform the jobs because he has time. So – some one applied and the electricity problem has solved .

I: It means , you call them all and sit together in a meeting to solve the problem .

P: No. I sit and meet only with the community group.

I: And what does the community support group ?

P: We do not discuss this issue with the CSG.

I: But what happened in this case to solve the problem ?

P: We share it with others and then they know the matter.

I: But we discuss the matter with them and seek help whether they can solve the problem. Now you tell me something about the meeting and subject matter of the discussion in the meeting of the CGs held once in a month. And the meeting of the CSG held twice in a month and what are the subject matter of the discussion ?

P: Mostly we discuss the various diseases in the meeting which is our top priority rather than other subject. The development and the source of fund is always in our hand. For example, the diseases may have various type which we discuss about diarrhoea, watery feces etc. We also discuss about TB. If the patient suffer in cough more than 3 weeks, then we examine the patient’s cough and told the patient to do the test as soon as possible.

I: It means, you talk about various health issues and other matters.

P: Yes.

I: Who leads all these meeting or under who’s guidance it helds?

P: All the meeting under CHCP’s auspices.

I: Who are those people are usually participated in this meeting. Do the members of the CG also participated.

P: Yes.

I: Who else are present in that meeting. Is there any local people participated in that meeting.

P: Sometime the local people are also present those who are having good relation with the CG. members. They requested them to attend the local people as saying the meeting is ongoing at Shatpara CC.

I: I in this case ,do you ever arrange any meeting separately with the male and female.

P: No. They all use to attend together irrespectively male an female.

I: Could you tell me what is the real reason of getting so much interest to attend this meeting or what they have said them, so they attend the meeting . What are subject matter, they take more interest or feel encourage and feel necessity to attend the meeting . What is the real attraction that they must join the meeting .

P: I told them that they should attend the CC meeting as they are government official.

I: They listen your instruction or words as you requested them.

P: Yes.

I: Now, I want to discuss with you which I think could be the subject matter of antibiotic resistance. The patients those ,who are coming to you, in that case, you are giving them the medication from your position, those who are coming to you with such problem but the people of this area are not knowing the matter. Say – one or two patients come to you from a certain household and you told them about antibiotic resistance. So- he or she comes to know the matter but the other people live beside them do not come to know about antibiotic resistance. So we are thinking to recruit some volunteer workers from this community , those who are interest to do this work.

Now tell me , as we wanted to get some volunteer workers in this regard from this area. Do you think that this type of workers would be available those who eager to work in this field. They will visit each household of this area and aware them about health care services .

P: It may not be possible to go each household but the many shops are there to inform and there are many household in one family.

I: Is it that volunteers?

P: Yes.

I: They work for whom and under which media? Who will elect them?

P: Those who are from that community group. No- they are from support group.

I: You are telling then the members of the community support group are volunteers?

P: Yes.

I: Do they provide any health serivces?

P: I informed them many things from this clinic and we also discussed about the diseases which they informed it later to most of the household.

I: So – they inform it each household.

P: Yes.

I: It means you informed them from your clinic and then they inform it to others.

P: Say- Someone inform in his own home, then he or she informs other household, which he or she knows.

I: So- they are working as volunteer.

P: There was no Imam of the Mosque before, we called the Imam Shaheb usually in our meeting and discussed various issues and requested him to say something about our health issue program when gets scope to inform it to people in the mosque.

I: What is the topic, you usually requested him for telling .

P: For example:, we told him mostly to talk about the bad effect of smoking to the people.

I: In that case , do you tell him anything about health issue? What else you request the Imam to tell?

P: I told him to say that smoking is harmful for health and it may cause cancer and its bad effect also influence the wife’s health , specially if the women is pregnant, then the chance of risk is heavier.

I: It means you want to say, Imams are also helping to work as volunteer. The members of the CSG are also working as volunteer. But say- the total 51 members are not perhaps working all together.

P: Yes- true. Not.

I: In this case, you can do it in a way to involve some of the members out of 51, those who are very active and hardy as a volunteer worker. In your opinion do you selected these type of people?

P: Elected means –we do it in locally. We told them informally. As a people of this village and locality, whatever we say – they listen and tell it other.

I: Well. Now we want to know from you that the selection of such volunteer worker, what quality they should have in your opinion to consider them as volunteer.

P: Education is the first and top priority criteria because if they are not educated, they will not be able to understand or to help other to understand or circular the messages to others. First priority is the education and some of them involve in village

lawgiver are good to select . They are usually involve in the legal meeting to solve any mutual conflict in villages . Then there are lots of people are also gathered there and in that time , they can say something about our health issues such as smoking . They can urge or request the people not to smoke and can narrate its harmful effect on health which may cause cancer or if anybody continues to smoking may always feel sick and those do not smoke around them may affected by indirect smoking . To smoking in front of the pregnant women may cause their health hazard and even children may also be affected.

I: I want a new idea from you that we think the regular meeting will be held with the members of the community support group or community group and who will call that meeting. It may be called by the volunteer or the facilitator in this community but not in your community clinic. So- now, we have got some volunteers with some six thousand population including the community clinic. We will select the people or volunteer, those will come from middle place of the community clinic and community support group. So, it could be easier to link with both, the community clinic and the volunteer. What do you think about this idea? How do you feel about it.

P: Those who will work as volunteer worker usually keep contact with the community clinic.

I: In this connection, the support group will work as intermediated to keep the continuous contact, What do you think? You are the person of the community clinic and I am the volunteer. Now the contact between you and me should be continued which will be created . The contact would be intermediated by the community support group. What do you feel about this idea?

P: That is good idea. Because the members usually visit the community clinic and the contact as you are saying that the people are always coming to take the service as this community clinic. What we say them here, they inform that to others in the village.

I: In that case, do you think, the members of the community support group will supervise the work of the volunteer in this area. What do you think about it?

P: Members of the community support group?

I: Yes.

P: Yes- It can be done.

I: Those who attend the meeting regularly whether they are informing other people in the community . Do you think , the community support group can supervise them?

P: Yes- can supervise, Member can also do it. Because who is a member of this area, he is but respected person and it is natural the volunteer will also obey or listen his words.

I: In that case, are they able to keep the information or maintain the data record? Whether they are regularly attending the meeting or not? Every body is participating in the meeting or not, and it held timely or not. So all these data may deposited in this community clinic . Yes- how many meeting held in a month by those volunteer and who those attended the meeting and the issues discussed . All these information, they will submit to you or in community clinic. Do you think, it would be possible?

P: In my opinion, it would be good.

I: If we think here that the particular service, the volunteers should be separately deal with the male and female candidates. What do you think about it? Do you think, It would be good?

P: I think, it would be good.

I: Why you are thinking it would be good?

P: Many times male work together but female do not feel like to work in front of male. That is why, if they works separately it would be good. Then they will work in their own interest.

I: In this case, what could be the benefit of the people of this community. We will select one volunteer for the male and another one for the female. So what do you think about this benefit for the people of this area.

P: If the female volunteer talks with the women, then it will be good and easy to listen the problems and they will be more open to discuss any issue. That is why, it would be good.

I: The women will feel more comfortable, is it?

P: Yes.

I: Now- another think- I want to know from you that those CHCP are working here, how can you attach them with us with the work? Would you tell us your point of view in this regard?

P: What is that work?

I: Say- that work is to inform all the people of this area about health awareness via volunteers. Here- do you think that the CHCP can play some role.

P: First we will discuss the existing problems in each monthly meeting with the community group members. Again we will discuss the same issue with the support group members , when we will call them for meeting with-

I: In that case , is it possible that the volunteer would be present in that meeting. And during the discussion, they will get some information or guidance from you?

P: Yes- that would be possible.

I: Well. It is all right. In that case you or the CHCP can do another thing is that to monitor the works of the volunteer whether they are doing their responsibilities or work properly or not. And what types of jobs, they are performing that whether it is good or bad that information should be monitored by you or the CHCP.

P: Yes. It is possible to monitor by the mobile or by real super vision.

I: Well. So It is clear that you will monitor and supervise the volunteer work or responsibilities simultaneously. I want to know a bit more about the volunteer which you have said that they should be well educated and another thing, you have mentioned that those lawgiver in the village are equally important to include as volunteer.

P: So- It is seen that they can handle or discuss about the diseases which is very frequently occur in the country or in our area.

I: Well. Now I want to say that our decision is to tame them as volunteer, but what are the things that can be motivated or encouraged them to giving these services. What could be done for their encouragement in this work, so that they will show more interest to join to work.

P: Is it those who will work as volunteer and for their encouragement.

I: Yes. Those whom we want to involve in this work. Whatever you think that tell me please.

P: What should I say? Volunteer are working in the country usually but even then we see something else.

I: Say- Now it I request someone please do this work and may be particularly tell him to give us 2-3 hours time in a week and also inform it to all. When he will perform the work then I told someone, what could be done to encourage him for continuation of 2 – 3 hours work in a week. And he will confirm us about his service that he will continue.

P: Everybody work against money which may count as salary , so when someone will work as volunteer might have some expectation.You see – nobody likes to work without money .Sometime in case of my necessity, I pay them some money againt their work .The amount is not so high but it does not matter . ( P-49 b con.

I: Whom you pat then ?

P: That is the volunteer, those who help in my work.

I: Please, tell me, who are those people ?

P: Say – there are some poor volunteer, whom I use to pay against their work .

I: What type of work , they usually do as per your instruction?

P: The work may be in our community clinic . Say – when a visitor will come to see our clinic , then we call them and take their help to clean the bushy place around the clinic.Then we pay them some money.

I: Well.Then you said that you paid some money . What would be the purpose to pay – as it will count as incentive or their travel purpose or as they will work 3 hours in a week or as it will count as honorium, what could be good in your opinion ?As you told , they will work 2-3 hours in a week , so in tour opinion, how much should be given and what would be the process? Please- you see, I am telling you the truth directly that we are taking the services from them not against money as they will provide service as a volunteer. But in a sense , tell that we want volunteer .

P: That is the work usually done sometime for my personal interest because it is essential to clean the bushy place around my clinic, which I already informed you .

I: That`s I want to say that we want volunteer and they will get some money . But the thing is not like that .We want them as volunteer . Now the work they will do by motivation may need to giving them some financial benefit, so that will encourage them to work . But the thing is not like that we are giving him for that reason only . It is perhaps for his encouragement that yes – if they do some work, they will get some benefit . In that case, you can tell me that what should be the amount and media , in which you will pay ?

P: No, I shall not tell the amount ?

I: They will work 2-3 hours in a week.

P: Yes- they can . Why can`t ?

I: In that case, what is your opinion about the amount to pay, please – could you give me any idea ?

P: No. I don`t have any idea about it .

I: Well. It is all right .

I: What are the ways or media by which people of this area may get their home healthcare services ? You have mentioned one only that at their home. What are the other way to get that services ? Once you told that they get the services from one local pharmacy and is there any other health worker provide such services at home ?

P: Yes- provide. There is a Hindu person. Who provides the healthcare service by going at home

I: Is he working under any organization ?

P: He does not work under in any organization . But he has been doing work before my birth in this area .

I: Does he work in any NGO?

P: No. He does not . But I think – he has got 2-3 months training from somewhere which may be right .

I: What does he do ?

P: He does not do anything . But sale drugs by walking in this area .

I: He sales drugs by walking ?

P: Yes.

I: Does he a Herbal Kobiraj type or paramedics ?

P: Perhaps Kobiraj type doctor . He is not a paramedics.He may learn something before but I did not ask him .What I think – perhaps he learned something before about medication for some diseases only .He keeps his drugs in a bag and sale it by walking at home .He does not have the scope to sale everybody. Those who do not understand , they buy his medicine. When some patients come to me and inform me about him (Kobiraj ), I forbid them not to buy drugs from that Kobiraj.

I: What type of medicine , he usually sales . Is it allopathic or herbal type ?

P: No. It is allopathic type drug .

I: They bought it from him .

P: Yes

I: Does he sale Antibiotic ?

P: I could not see but may be sale .He might sale some antibiotic which is natural.

I: Well – it means, people may get antibiotic from other source.

P: I have got the news that there is a person who has a shop and often comes here to me in the afternoon.This incident may be only 3-4 days or a week ago .

I: Is there anything more that any women or TBA or any NGO provide this health services to pregnant women or children by going at their home .

P: There was a foreign organization worked here before. Now- there is a Surjer Hasi ( NGO ) at Shahidnagar in front of the road in which way, you come here .

I: Is that organization still working or it was gone away?

P: Still existing the Surjer Hasi Community Clinic. A woman, who is our neighbor working now there. She lives beside our home and giving health services to other women, those who seek medication to her. Male People usually do not visit her but women only.

I: Does she visit the each and every household?

P: No. She does not visit the household. You see- when women use to visit her at home are having various problems. Now- she has got the job in Upazilla Health Complex. Perhaps she completed a medical course. She worked few years in the Surjer Hasi Clinic. Now she is working there (UHC) as Nurse as she officially recruited.

I: Well. Tell me the distance of the Surjer Hasi Community Clinic from your clinic? How much time it may need to reach there?

P: It is not so far from here to reach if you use your vehicle may take 5 minutes, not more than that. The road communication is good.

I: So- I have understood that the people of this area may get services from that Surjer Hasi Community Clinic also as well.

P: Yes. May get.

I : Is there any more places to get health care service here?

P: There is a pond middle of the road, where another one shop is situated. A Hindu person, who also treats with herbal medicine there. He treated the patients with various type of syrup as we also give normal paracetamol syrup or tablet. He gives only syrup type medication.

I: What could be the distance from here?

P: It is not so far from here. Very near.

I: How much time, it may take to reach there?

P: It is a walking distance for 5 minutes for a male.

I: You told me about the Surjer Hasi Community Clinic and then Herbal medication shop, is there any Homeopathic, in an around in your locality.

P: Yes. There is a Homeopathic medical shop at Daspara which is not so far from here. People are also seek medication there.

I: Who are else that Paramedics or representatives?

P: There is no any other Paramedics here but in Shahidnagar. The boy is from our area who run the clicnic. He is studying and open a clinic where people are also visit for medication.

I: Is it a clinic?

P: Yes- Clinic.

I: How much time, it may take to reach there?

P: It is there on the main road by which you reached here. The shop is on the station by which you came here.

I: Do the people of this area use to go there for treatment?

P: Yes- they go. Though he is not a doctor but studying Paramedics. He is the boy from our area. His chamber is there, so patients visit him there.

I: Is there any more such thing in this area? Kobiraj?

P: Yes- There is a Kobiraj.

I: Where is that Kobiraj?

P: The Kobiraj is sitting in front of the chairman house. There is a building near that chairman house after a mosque, to that house the Kobiraj is working.

I: You told that people takes the medication from that place.

P: Yes.

I: You have informed me the name of the persons, so far- are they treated the patients with antibiotic?

P: No. Don’t.

I: Tell me, your opinion about the hearth care service, you are providing which will be more acceptable to the patients? What those ways, it could be done? It may be to inform it to them or discussion or by any picture showing or poster or any leaflet.

P: It is better if we use calendar as for example- when we have got it during our training. When the patients, come here, then we show it to them how to keep clean the house, how to clean the stencils before cooking and tell them also to clean the plate.

I: Do you think, they like your advice or the picture.

P: They also see the pictures. And here, they may not understand if we present it to them in other way. Because the people of the village will understand, if we present it to them in easy way.

I: You told that you would be able to motivate them. The way you selected them or how you will selected them for the work? You may bring some people from that 51 members already in your hand and whom you can request.

P: That is our strong and active members in our community group. When we will sit in a meeting together, then it could be done nicely. Whom we can share the work and who will perform it, then we both will come to know. It is possible to do the work with them.

I: Do you think that the way, you will select them, the people will like it? The people will accept it whether your selection is right or wrong?

P: Yes- They will accept and why they will not accept? Because it is a healthcare service. It is essential to all to know. Because many people know it and many are not. We provide the healthcare service in this community clinic and they will also be interested about it. But who are those do not come, they will not come to know. Those who works in the field with healthcare service, they will appreciated this program.

I: All right. Thank you.

I: How much time, the members of the community support group are specially spending in this case and behind the services of the community clinic. How much time, they (Community Support Group) are spending in case of supporting the community clinic.

P: I informed at first time that it is almost one hour.

I: Say- to come here to take some information or to know something from you by discussion. How much time do they spend in this purpose. Do they come regularly or how many days in a week or how long time do they stay here, when they come. And what are the topics they wanted to know?

P: You see, they do not come regularly. And sometime, they come in between work and ask the working condition and whether the services are giving properly or not. They take care about the availability of drugs. They wanted to know all this things.

I: How often, do they come in this way? could you gauge how many days, do they visit in this way.

P: You mean- in a week in this area.

I: It means, how many days, do they come here on average in a week and how much time do they spend here.

P: They do not spare more than 15-20 minutes.

I: And how many days, they visit in a week?

P: How many days, should I say in a week? They are usually come here always. You can assume 7-8 times in a week.

I: Is it in a day or week?

P: Yes.

I: It means 7-8 times in a day or it may also in a week.

P: They discuss various issues, when they come to take the drugs. Because they take the drugs from me, when they get sick. Then they also discuss the various issues of healthcare services.

I: The members of the community group come here 7-8 times in a week.

P: Yes.

I: The members of the support group do also the same?

P: No. They do not come so frequently.

I: How often, they do come?

P: They usually come often and not so frequently.

I: What do you mean by lesser time or often? Do they visit here weekly or monthly basis?

P: As per our schedule, we usually do the meeting after every two months. But sometime they come here, when they feel necessary. And when we call them in meeting, then they attend it.

I: Thank you for giving us the time. We discussed in many issues. O.K. Stay blessed.
