# Supplementary material for: A process for developing a sustainable and scalable approach to community engagement: community dialogue approach for addressing the drivers of antibiotic resistance in Bangladesh
Source: BMC Public Health. 2020 Jun 17;20:950. doi: 10.1186/s12889-020-09033-5 (PMC7302129; doi:10.1186/s12889-020-09033-5)
Supplement: Supplementary file 17 — Additional file 17. CHCP 14.04.17. Interview guide for community health care practitioner [file 12889_2020_9033_MOESM17_ESM.docx]

**IN-DEPTH INTERVIEWS WITH COMMUNITY HEALTH CARE PROVIDER: TOPIC GUIDE**

***Introduction***

*Welcome with the following:*

Good morning / afternoon, my name is ______ [and this is my colleague _____]. Thank you very much for agreeing to be part of this discussion. We are from ARK Foundation, which is a research organisation, and we are working on behalf of the government, particularly Community Clinics Unit. [within the Ministry of Health and Family Welfare, as well as two organisations from the UK - the University of Leeds and Malaria Consortium]. We are providing some technical support to the government to help your community. For example, we will provide some training and some materials that will help the government to provide some health education about the use of antibiotics. Participation in the study is an opportunity for you and other people to tell us your experiences, opinions and ideas. We value different points of view.

*Explain some key points of process:*

- If you would like to stop the interview at any time, that is absolutely fine and you do not have to explain why.
- We will not identify you by name in anything that we write, so please feel free to speak openly.
- Explain how the recorder is used
- Confirm that the participant has received and understood the information sheet
- Confirm that the participant has signed the consent form.

*Announce that the interview will now start*

*Switch on the recorder*

*Speak into the recorder the information below:*

Community clinic ID:

Name of interviewer:

Start time:

***Part One: Antibiotic Use***

1. Please can you tell me what people in this community do when they are not feeling well?

*Probes:*

- *Where do people in this community go (CHCP, UHC, pharmacy, medical representative traditional healer / homeopaths, family member or friend)?*
- *Why do they do this?*
- *Do different types of people do different things: Try to unpack for at least infants aged under 1, children aged 1-5, other children, pregnant women, women of reproductive age, men of working age, older women, and older men.*
- *If homeopathy or healers are mentioned, probe whether people are seeking care appropriately e.g. by asking what kind of symptoms they have when they access these sources of care*

1. I am interested in your experiences of prescribing medicine. Please can you tell me about the common conditions that people present with in this area? Please can you tell me which ones usually require medicine?

*Probes:*

- *Do you always prescribe medicine, and why or why not?*
- *Do people in this community have preferences for particular types of medicine, and why?*

1. Please can you tell me which antibiotics you most often prescribe, to whom and for what conditions?

*Note to interviewer, if the CHCP is unclear which medicines are antibiotics, then supply them with this list: (insert list here)*

1. When you do need to prescribe antibiotics, what do you usually tell the patients about taking their medicine?

*Probes:*

- *Completing the course even if you feel better?*
- *Sharing the antibiotics with anyone else?*
- *Keeping leftover antibiotics if they didn’t complete the course?*
- *The correct dosage i.e. for how many days and how many times a day?*
- *If the supply is inadequate, what do you tell the patients to do (e.g. return to collect the remainder of the prescription, or acquire from another source such as UHC, pharmacy, medical representative)?*
- *Why do you tell them these things (i.e. we are looking to understand whether they have any idea about antibiotic resistance)?*
- *If the patients are going to collect the antibiotics from another source do you write the prescription down for them?*

1. If patients do need antibiotics, then where do they get them from?

*Probes:*

- *If they get a full prescription from the CHCP, do they take accept this, or do they choose to go elsewhere (such as the UHC, pharmacy, medical representative) to acquire them and why?*
- *If they are offered a partial prescription from the CHCP, what do they do e.g. return to collect the remainder, or go somewhere else and if so where (such as the UHC, pharmacy, medical representative), use leftovers / share, or not come back)?*
- *If there are no antibiotics at the Community Clinic, what do patients do (e.g. nothing, go somewhere else and if so where (such as the UHC, pharmacy, medical representative), or use leftovers / share?*

1. Do you ever experience situations where people have symptoms that do not require treatment with antibiotics, but they ask you to provide them? Can you tell me about that and what you do in those situations?

*Probes:*

- *Does this happen more commonly with particular types of patients (women, men, parents)?*
- *Is it generally easy or difficult to manage patients in this situation and why?*
- *What specifically do you say to them i.e. do you have any particular explanations that you give?*
- *Do you sometimes give them antibiotics anyway, or any other medicine (and if so, which ones)?*

1. Can you tell me about how patients respond if you tell them that they do not need antibiotics?

*Probes:*

- *Do they accept your explanation?*
- *Do they insist or become angry?*
- *Do you think that they go somewhere else and, if so, where do you think they go?*
- *Do you think they use leftovers or share with other people?*
- *Is there a difference between how different types of patients react?*

1. Can you tell me whether people can acquire antibiotics without a prescription and how easy or difficult that is?

*Probes:*

- *Where can they acquire antibiotics without a prescription?*

1. Do you think that patients do as you advise them to do? Why or why not?

*Probes:*

- *Try to unpack the reasons for different types of people, especially asking about reasons that children under the age of five might not complete the treatment and if these reasons are the same or different than those for older children and adults.*
- *Ask specifically about whether they think patients complete the course of antibiotics and why or why not*
- *Ask specifically about what they think patients do with the antibiotics if they have not completed the course. Do they keep them for later use (for themselves or others), do they share them?*

1. Do you know what might happen if someone does not complete the full does of antibiotics that they have been given?

*Probes*

- *(They may say something about individuals, but probe if they have any idea what the impact can be on the population level)*
- *If they have an idea, ask them how they know this.*

1. Have you ever heard the term “antibiotic resistance”? Can you tell me what you know?
2. (If they CHCP understands antibiotic resistance, then ask: Do you think that patients understand what antibiotic resistance is?)

***Part Two: Potential Intervention***

1. Thank you very much for sharing your experiences and knowledge about antibiotics. We intend to develop a community based project to address the misuse of antibiotics. In addition to the work that you conduct within the community clinics, please can you tell me what other work you do as a CHCP?

*Probes:*

- *Do you deliver any health education in the community (like a courtyard meeting – uthan boithak)?*

1. I am very interested to learn about the community group and community support groups. Please can you tell me about the community group and community support group for this clinic?

*Probes:*

- *Who belong to the groups?*
- *How are they selected (probe especially for the members who do not belong to a specific category)?*
- *Who is responsible for selecting them?*
- *What are the regular activities of the groups?*
- *How much time to the members usually give to the activities of the groups?*
- *Are some members particularly active and if so, why, if not, why not?*
- *What happens if a member of the community group or community support group does not want to participate any more?*
- *Who supervises and monitors the work of the community groups and community support groups?*

1. I am really interested to understand more about the links between the community group, community support groups and the community clinic. Please can you tell me about that?
2. *(a) In communities where the CSGs are functional:*

I have learned that the community support groups in this area are very active. Please can you tell me about why you think this is the case? Please can you also tell me if this community support group ever faces any challenges in carrying out their responsibilities? How do they try to overcome those challenges and what more could be done to help to overcome those challenges?

*(b) In communities where the CSGs are partially functional:*

I have learned that this community support groups are quite active and that they are usually able to complete some or most of the activities that they are supposed to. Please can you tell me about what you think helps this community support group to complete their activities? Please can you also tell me what the challenges are that they face in carrying out their responsibilities? How do they try to overcome those challenges and what more could be done to help to overcome those challenges?

*(c) In communities where the CSGs are not functional:*

I understand that there are sometimes challenges in being able to complete the activities that the community support groups have been asked to complete. Please can you tell me what the challenges are that they face in carrying out their responsibilities? How do they try to overcome those challenges and what more could be done to help to overcome those challenges?

Please can you tell me about any meetings that are currently held within the community to discuss health issues?

Focus especially on the courtyard meetings (with health assistant?), the community clinic meetings (with CHCP), and the health education with members of the community support group. Use the probes below to find out as much as you can about these.

Use this section of the focus group discussion to find out how people learn about health issues.

Ask specifically if courtyard meetings are held and, if not, why not.

Probe:

- *What types of issues are discussed in these meetings?*
- *Who initiates the meetings? Who is responsible for organising them? Who is involved in mobilising participants?*
- *Who usually participates in these types of meetings e.g. men, women, older people, younger people?*
- *Are there separate meetings for males and females, or does everyone attend the same ones?*
- *How often do they occur (weekly, monthly)?*
- *What time of day are they held and is it always the same time?*
- *How long do they last?*
- *Where are they held?*
- *What do you think motivates people to participate in these types of meetings (probe for men, women, older people, younger people)?*
- *What sorts of challenges do you think prevents people from participating in these types of meetings (probe for men, women, older people, younger people)?*

Also, explain what we plan to do, and find out what they think is the best way to reach the whole population.

1. In the project that we plan to deliver, we will be interested in recruiting people who can facilitate regular meetings in which health issues will be discussed. So, I would like to ask you some questions about any existing volunteers in this area. Can you tell me if there are already people who work as volunteers in this area, who they are, and what they do?

Probe:

- - *How many male and female volunteers work on health (and other) issues? Is it useful to recruit both male and female volunteers?*
  - *Who identifies / selects these volunteers (please explain exactly how the process works, and who helps to identify / select them)? Do you think this process works well and people are happy with it?*
  - *Do you think that people are happy with the way volunteers are identified / selected? If not, what do you think could be done differently?*
  - *Who supervises the volunteers and the work that they do? Do you think that this supervision system works well?*
  - *Are the volunteers linked to the health system?*
  - *What do you think motivates volunteers to work?*
  - *Do volunteers receive any kind of incentive for their work e.g. a payment, or the costs of travel, per diems etc)*

1. *(a) In communities where the CSG is functional or partly-functional:*

I think that it might be possible for the CSG to be a link between the facilitators of the regular meetings and the community clinics. What do you think about this idea? How do you think this link could work?

Probe:

- - *Do you think that the CSG members could supervise the work of the volunteers that are facilitating the regular meetings?*
  - *If not, then who do you think could do so?*
  - *Do you think that the CSG members could keep simple records of the activities that take place at the regular meetings and feed them back to the community clinic?*
  - *If not, then who do you think could do so?*

*(b) In communities where the CSG is not functional:*

I need to think about who could provide a link between the facilitators of the regular meetings and the community clinics. What are your thoughts on this?

*Probes:*

- - *Who might be able to supervise the work of the volunteers that are facilitating the regular meetings?*
  - *Who might be able to keep simple records of the activities that take place at the regular meetings?*
  - *Do you think that this is something that the members of the CSG could do? What would help to make this possible?*

1. Can you tell me how you think a CHCP could be involved in this intervention?

*Probes:*

- - *Could the CHCPs provide any guidance to the facilitators, for example, if they do not understand an issue properly and need some support?*
  - *Could the CHCPs collect simple monitoring data and take responsibility for passing it to the person responsible for reviewing the data?*
  - *Could the CHCPs be involved in supervising the work of the facilitators?*

1. Finally, I would like to understand more about the ways that people in this area currently learn about health issues. Please can you tell me about that?

*Probes*:

- *Who delivers health information e.g. community health workers, volunteers, community health centre staff, private providers, village leaders, teachers, or through mass media such as television and radio?*
- *What is the format in which the information is provided e.g. discussion, printed materials such as posters, flip charts, leaflets?*
- *What do you think people trust, both in terms of the people delivering the information, and in terms of the format of the information?*
- *Do people prefer photos or drawings?*

Close the interview by asking the participant whether he / she has any questions or any further information that they would like offer. Thank him / her for their time.
