# Supplementary material for: A process for developing a sustainable and scalable approach to community engagement: community dialogue approach for addressing the drivers of antibiotic resistance in Bangladesh
Source: BMC Public Health. 2020 Jun 17;20:950. doi: 10.1186/s12889-020-09033-5 (PMC7302129; doi:10.1186/s12889-020-09033-5)
Supplement: Supplementary file 18 — Additional file 18. CSG 14.04.17. Focus group discussion guide for community support group [file 12889_2020_9033_MOESM18_ESM.docx]

**FOCUS GROUP DISCUSSIONS WITH COMMUNITY SUPPORT GROUP MEMBERS: TOPIC GUIDE**

***Introduction***

*Welcome the participants with the following:*

Good morning / afternoon, my name is ______ and this is my colleague _____. Thank you very much for agreeing to be part of this discussion. We are from ARK Foundation, which is a research organisation, and we are working on behalf of the government, particularly Community Clinics Unit. [within the Ministry of Health and Family Welfare, as well as two organisations from the UK - the University of Leeds and Malaria Consortium]. We are providing some technical support to the government to help your community. For example, we will provide some training and some materials that will help the government to provide some health education about the use of antibiotics. Participation in the study is an opportunity for you and other people to tell us your experiences, opinions and ideas. We value different points of view.

Ask participants to introduce themselves.

**Offer the participants a refreshment.**

*Explain some key points of process:*

- We would like you to do the talking. We would like everyone to contribute their ideas.
- There are no right or wrong answers. All your experiences and ideas are important.
- You can leave the discussion at any time and you do not have to explain why.
- We will not identify anyone by name in anything that we write, so please feel free to speak openly.
- Explain how the recorder is used
- Confirm that participants have received and understood the information sheet
- Confirm that participants have signed the consent form.
- In case a participant has not signed the consent form, because they are not literate, ensure that consent has been verbally recorded.

*Announce that the discussion will now start*

*Switch on the recorder*

*Speak into the recorder the information below:*

Community clinic ID:

Name of interviewer:

Name of note-taker:

Start time:

***Part One: Antibiotic Use***

1. I would like to learn about how people in this community access health care. We are going to create a picture or a “map” of the places where people can access health care. We will start this map now, but we will add to it during the discussion.

*Probes:*

- *Where do people in this community go (CHCP, UHC, pharmacy, medical representative traditional healer / homeopaths, family member or friend)?*
- *Why do they do this?*
- *Try to understand if there are differences for different types of people, with a focus on children under the age of five versus others.*
- *If homeopathy or healers are mentioned, probe whether people are seeking care appropriately e.g. by asking what kind of symptoms they have when they access these sources of care*

*Note for facilitator: This is, in part, a warm-up participatory exercise, but will also provide useful information to return to later in the discussion.*

*Provide a large piece of paper, and a pen for each participant (this could also be done, if preferred, using solid objects placed on the ground to represent the items on the map).*

*Ask the participants to start to draw a map by:*

*Asking them to draw a circle or place an object in the middle of the page to represent the village / setting that the FGD is taking place in.*

*Then ask them to indicate the different providers of health care that people in this area usually access. Draw them onto the map, approximately in the locations they are, relative to the village. Ask participants to use different signs or objects (which you can agree on) to identify different types of provider.*

*Make sure you probe for public and private providers (e.g. community clinic, upazila health complex, pharmacies, medical representative)*

*Do not restrict this only to formal biomedical providers. Allow and encourage participants to include informal (traditional) providers too if they choose to.*

1. I would like to ask you some questions about what you know about medicines. Are you aware about different types of medicines?

*Probes:*

- *Do people classify according to “strong” and “weak” and what belongs in different categories?*
- *Do people classify according to capsule, tablet, liquid, cream etc*
- *Do people classify according to antibiotics and non-antibiotics?*
- *(If “antibiotic” is mentioned, then ask for the name – to check if people have an idea about which medicines are antibiotics)*

1. Does anyone here know what the difference is between antibiotics and other medicines?

*Probes:*

*- Do you have any idea why people are sometimes given antibiotics and sometimes given other types of medicines?*

*Note for facilitator: Allow the participants to answer this question. Once they have offered their response, you can explain or reiterate that: when a person becomes ill, the illness might be caused by an infection with some small bacteria that attack the body. The body tries to fight against these bacteria itself but it sometimes needs some extra help. Antibiotics are a type of medicine that can help to fight the bacteria. Not all illnesses are caused by bacteria that antibiotics can fight and so it is very important to get the correct medicine for the illness.*

*Note to interviewer: Question and probes need to be adapted according to knowledge of antibiotics*

1. I would like to learn about what happens when people are given medicine / antibiotics. Please can you explain whether people usually receive any information about how they should take them?

*Probes:*

- *Completing the course even if you feel better?*
- *Sharing the medicine / antibiotics with anyone else?*
- *Keeping leftover medicine / antibiotics if they didn’t complete the course?*
- *The correct dosage i.e. for how many days and how many times a day?*
- *If the supply is inadequate, what are the people told to do (e.g. return to collect the remainder of the prescription, or acquire from another source such as UHC, pharmacy, medical representative)?*
- *If people are asked to collect the prescription from another source, is the prescription written down for them?*

1. I would like to return to the map that we have created of the different health care providers. Please can you show me on the map where people in this community go to get medicine / antibiotics like the ones I have shown you?

*Probes:*

- *If they are provided with the full supply, do they take accept this, or do they choose to go elsewhere (such as the UHC, pharmacy, medical representative) to acquire it and why?*
- *If they are offered a partial prescription, what do they do e.g. return to collect the remainder, or go somewhere else and if so where (such as the UHC, pharmacy, medical representative), use leftovers / share, or not come back)?*
- *If there are no antibiotics, what do patients do (e.g. nothing, go somewhere else and if so where (such as the UHC, pharmacy, medical representative), or use leftovers / share?*

1. Please can you tell me whether it is easy or difficult to get medicine / antibiotics?

*Probe*s:

- *Ask participants if there are some providers where it is easy to access medicine / antibiotics, and some where it is more difficult. If they identify differences, ask them why they think there are differences.*
- *Ask participants whether they usually or always need a written prescription in order to get medicine / antibiotics.*
- *Do you know whether people in this area prefer some of these medicines / antibiotics more than others and, if so, why?*

1. Please can you tell me how you think that people feel if they are not feeling well and they visit a health care provider and are not given any medicine? Can you tell me what people usually do in that situation?

*Probes:*

- *Do they accept the explanation?*
- *Do they insist or become angry?*
- *Do you think that they go somewhere else and, if so, where do you think they go?*
- *Do you think they use leftover medicines or share with other people?*
- *Is there a difference between how different types of patients react?*

1. I would like to learn about whether people usually follow the advice that they are given regarding the way they should take medicine / antibiotics.

*Probe*s:

- *Try to understand whether the issues are different for different types of people and different conditions.*
- *Try to understand the reasons that people do or do not follow the advice that they are given.*

1. Please can you explain whether people usually complete the course of medicine / antibiotics that they have been prescribed?

Probe:

- *Try to understand whether the response is different for different types of people and different conditions.*
- *Try to understand the reasons that people do or do not complete the course of medicine / antibiotics (note, that it is quite common for people to stop taking them once they feel better, or maybe the medicine has side effects, or perhaps it makes their children vomit)*

1. If people do not complete the course of medicine / antibiotics, please can you explain what they usually do with the medicines that are left over?

Probe:

- *Try to find out if they throw them away or keep them.*
- *If they keep them, why do they keep them and what do they do with them e.g. might they be used by the same person or a different person for a similar or different condition in the future?*

1. Do you know what might happen if someone does not complete the full does of antibiotics that they have been given?

*Probes*

- *(They may say something about individuals, but probe if they have any idea what the impact can be on the population level)*
- *If they have an idea, ask them how they know this.*

1. Has anyone ever heard the term “antibiotic resistance”? Does anyone know what “antibiotic resistance” means?

*Note for facilitator: Allow the participants to answer this question. Once they have offered their response, you can explain that: there is often a lot of confusion about what antibiotic resistance means. It means that bacteria have changed in response to the medicines that we use to treat the infections that they cause. Because the bacteria have changed, the medicines do not work so well (or at all) and this means that the medicines cannot help to treat or prevent the infection because they can no longer fight the bacteria.*

***Part Two: Potential Intervention***

1. I would like to understand this area. Please can you explain the administrative breakdown of the area?

*Probes*

*Ask about ward, mahalla / para etc*

1. Please can you tell me about any meetings that are currently held within the community to discuss health issues?

Focus especially on the courtyard meetings (with health assistant?), the community clinic meetings (with CHCP), and the health education with members of the community support group. Use the probes below to find out as much as you can about these.

Use this section of the focus group discussion to find out how people learn about health issues.

Ask specifically if courtyard meetings are held and, if not, why not.

Probe:

- *What types of issues are discussed in these meetings?*
- *Who initiates the meetings? Who is responsible for organising them? Who is involved in mobilising participants?*
- *Who usually participates in these types of meetings e.g. men, women, older people, younger people?*
- *Are there separate meetings for males and females, or does everyone attend the same ones?*
- *How often do they occur (weekly, monthly)?*
- *What time of day are they held and is it always the same time?*
- *How long do they last?*
- *Where are they held?*
- *What do you think motivates people to participate in these types of meetings (probe for men, women, older people, younger people)?*
- *What sorts of challenges do you think prevents people from participating in these types of meetings (probe for men, women, older people, younger people)?*

Also, explain what we plan to do, and find out what they think is the best way to reach the whole population.

1. I would like to understand more about the ways that people in this area currently learn about health issues. Please can you tell me about that?

*Probes*:

- *Who delivers health information e.g. community health workers, volunteers, community health centre staff, private providers, village leaders, teachers, or through mass media such as television and radio?*
- *What is the format in which the information is provided e.g. discussion, printed materials such as posters, flip charts, leaflets?*
- *What do you think people trust, both in terms of the people delivering the information, and in terms of the format of the information?*
- *Do people prefer photos or drawings?*

1. We are interested in recruiting people who can facilitate regular meetings in which health issues will be discussed. So, I would like to ask you some questions about any existing facilitators / volunteers in this area. Can you tell me if there are already people who work as facilitators / volunteers in this area?

Probe:

- - *What are the different types of facilitators / volunteers?*
  - *What do they do?*
  - *How many male and female volunteers are there? Is it useful to recruit both male and females s?*
  - *Who identifies / selects these facilitators / volunteers (please explain exactly how the process works, and who helps to identify / select them)?*
  - *Do you think that people are happy with the way facilitators / volunteers are identified / selected? If not, what do you think could be done differently?*
  - *Who supervises the facilitators / volunteers and the work that they do? Do you think that this supervision system works well?*
  - *What do you think motivates facilitators / volunteers to work?*
  - *Imagine if a facilitator / volunteer spend around 2-3 hours per week, then what kind of incentive for their work might be required e.g. a payment, or the costs of travel, per diems etc)?*
  - *Are the facilitators / volunteers linked to the health system?*

1. I am very interested to learn about the community group and community support groups. Please can you tell me about the community group and community support group for this clinic?

*Probes:*

- *Which areas does each CSG cover?*
- *Who belong to the groups?*
- *How are they selected (probe especially for the members who do not belong to a specific category)?*
- *Who is responsible for selecting them?*
- *What are the regular activities of the groups?*
- *How much time to the members usually give to the activities of the groups?*
- *Are some members particularly active and if so, why, if not, why not?*
- *What happens if a member of the community group or community support group does not want to participate any more?*
- *Who supervises and monitors the work of the community groups and community support groups?*

1. I am very interested to understand more about the links between the community group, community support groups and the community clinic. Please can you tell me about that?
2. *(a) In communities where the CSGs are functional:*

I have learned that the community support groups in this area are very active. Please can you tell me about why you think this is the case? Please can you also tell me if this community support group ever faces any challenges in carrying out their responsibilities? How do they try to overcome those challenges and what more could be done to help to overcome those challenges?

*(b) In communities where the CSGs are partially functional:*

I have learned that this community support groups are quite active and that they are usually able to complete some or most of the activities that they are supposed to. Please can you tell me about what you think helps this community support group to complete their activities? Please can you also tell me what the challenges are that they face in carrying out their responsibilities? How do they try to overcome those challenges and what more could be done to help to overcome those challenges?

*(c) In communities where the CSGs are not functional:*

I understand that there are sometimes challenges in being able to complete the activities that the community support groups have been asked to complete. Please can you tell me what the challenges are that they face in carrying out their responsibilities? How do they try to overcome those challenges and what more could be done to help to overcome those challenges?

1. *(a) In communities where the CSG is functional or partly-functional:*

I think that it might be possible for the CSG to be a link between the facilitators of the regular meetings and the community clinics. What do you think about this idea? How do you think this link could work?

Probe:

- - *Do you think that the CSG members could supervise the work of the volunteers that are facilitating the regular meetings?*
  - *If not, then who do you think could do so?*
  - *Do you think that the CSG members could keep simple records of the activities that take place at the regular meetings and feed them back to the community clinic?*
  - *If not, then who do you think could do so?*

*(b) In communities where the CSG is not functional:*

I need to think about who could provide a link between the facilitators of the regular meetings and the community clinics. What are your thoughts on this?

*Probes:*

- - *Who might be able to supervise the work of the volunteers that are facilitating the regular meetings?*
  - *Who might be able to keep simple records of the activities that take place at the regular meetings?*
  - *Do you think that this is something that the members of the CSG could do? What would help to make this possible?*

Close the focus group discussion by asking participants whether they have any questions or any further information that they would like offer. Thank them for their time.

If maps and diagrams were drawn on paper, then ask the participants if they would like to keep them. If so, ask if you make take a photograph for your records of the discussion and, if not, ask if you may take them paper with you. If maps and diagrams were created using objects, then ask the participants if you may take a photograph for your records of the discussion
