# Supplementary material for: A process for developing a sustainable and scalable approach to community engagement: community dialogue approach for addressing the drivers of antibiotic resistance in Bangladesh
Source: BMC Public Health. 2020 Jun 17;20:950. doi: 10.1186/s12889-020-09033-5 (PMC7302129; doi:10.1186/s12889-020-09033-5)
Supplement: Supplementary file 19 — Additional file 19. Union Parishad Chairman14.04.17. Interview guide for Union Parishad Chairman [file 12889_2020_9033_MOESM19_ESM.docx]

**IN-DEPTH INTERVIEWS WITH UNION PARISHAD CHAIRMAN: TOPIC GUIDE**

***Introduction***

*Welcome with the following:*

Good morning / afternoon, my name is ______ and this is my colleague _____. Thank you very much for agreeing to be part of this discussion. We are from ARK Foundation, which is a research organisation, and we are working on behalf of the government, particularly Community Clinics Unit. [within the Ministry of Health and Family Welfare, as well as two organisations from the UK - the University of Leeds and Malaria Consortium]. We are providing some technical support to the government to help your community. For example, we will provide some training and some materials that will help the government to provide some health education about the use of antibiotics. Participation in the study is an opportunity for you and other people to tell us your experiences, opinions and ideas. We value different points of view.

*Explain some key points of process:*

- If you would like to stop the interview at any time, that is absolutely fine and you do not have to explain why.
- We will not identify you by name in anything that we write, so please feel free to speak openly.
- Explain how the recorder is used
- Confirm that the participant has received and understood the information sheet
- Confirm that the participant has signed the consent form.

*Announce that the interview will now start*

*Switch on the recorder*

*Speak into the recorder the information below:*

Community clinic ID:

Name of interviewer:

Start time:

***Part One: Potential Intervention***

1. I would like to understand this area. Please can you explain the administrative breakdown of the area?

*Probes*

- *Ask about sub-district, union, ward, etc*

1. Please can you tell me about any meetings that are currently held within the community to discuss health issues?

Focus especially on the courtyard meetings (with health assistant?), the community clinic meetings (with CHCP), and the health education with members of the community support group. Use the probes below to find out as much as you can about these.

Use this section of the focus group discussion to find out how people learn about health issues.

Ask specifically if courtyard meetings are held and, if not, why not.


Probe:

- *What types of issues are discussed in these meetings?*
- *Who initiates the meetings? Who is responsible for organising them? Who is involved in mobilising participants?*
- *Who usually participates in these types of meetings e.g. men, women, older people, younger people?*
- *Are there separate meetings for males and females, or does everyone attend the same ones?*
- *How often do they occur (weekly, monthly)?*
- *What time of day are they held and is it always the same time?*
- *How long do they last?*
- *Where are they held?*
- *What do you think motivates people to participate in these types of meetings (probe for men, women, older people, younger people)?*
- *What sorts of challenges do you think prevents people from participating in these types of meetings (probe for men, women, older people, younger people)?*

Also, explain what we plan to do, and find out what they think is the best way to reach the whole population.

1. I would like to understand more about the ways that people in this area currently learn about health issues. Please can you tell me about that?

*Probes*:

- *Who delivers health information e.g. community health workers, volunteers, community health centre staff, private providers, village leaders, teachers, or through mass media such as television and radio?*
- *What is the format in which the information is provided e.g. discussion, printed materials such as posters, flip charts, leaflets?*
- *What do you think people trust, both in terms of the people delivering the information, and in terms of the format of the information?*
- *Do people prefer photos or drawings?*

1. We are interested in recruiting people who can facilitate regular meetings in which health issues will be discussed. So, I would like to ask you some questions about any existing facilitators / volunteers in this area. Can you tell me if there are already people who work as facilitators / volunteers in this area?

Probe:

- - *What are the different types of facilitators / volunteers?*
  - *What do they do?*
  - *How many male and female volunteers are there? Is it useful to recruit both male and females s?*
  - *Who identifies / selects these facilitators / volunteers (please explain exactly how the process works, and who helps to identify / select them)?*
  - *Do you think that people are happy with the way facilitators / volunteers are identified / selected? If not, what do you think could be done differently?*
  - *Who supervises the facilitators / volunteers and the work that they do? Do you think that this supervision system works well?*
  - *What do you think motivates facilitators / volunteers to work?*
  - *Imagine if a facilitator / volunteer spend around 2-3 hours per week, then what kind of incentive for their work might be required e.g. a payment, or the costs of travel, per diems etc)?*
  - *Are the facilitators / volunteers linked to the health system?*

1. Please can you tell me what you know about the community clinic, the community group, and the community support groups?

*Note to interviewer: If the union parishad chairman is knowledgeable on these topics, then ask him the questions below. If he is not knowledgeable, you can close the interview.*

1. I am very interested to learn about the community group and community support groups. Please can you tell me about the community group and community support group for this clinic?

*Probes:*

- *Which areas does each CSG cover?*
- *Who belong to the groups?*
- *How are they selected (probe especially for the members who do not belong to a specific category)?*
- *Who is responsible for selecting them?*
- *What are the regular activities of the groups?*
- *How much time to the members usually give to the activities of the groups?*
- *Are some members particularly active and if so, why, if not, why not?*
- *What happens if a member of the community group or community support group does not want to participate any more?*
- *Who supervises and monitors the work of the community groups and community support groups?*

1. I am very interested to understand more about the links between the community group, community support groups and the community clinic. Please can you tell me about that?
2. *(a) In communities where the CSGs are functional:*

I have learned that the community support groups in this area are very active. Please can you tell me about why you think this is the case? Please can you also tell me if this community support group ever faces any challenges in carrying out their responsibilities? How do they try to overcome those challenges and what more could be done to help to overcome those challenges?

*(b) In communities where the CSGs are partially functional:*

I have learned that this community support groups are quite active and that they are usually able to complete some or most of the activities that they are supposed to. Please can you tell me about what you think helps this community support group to complete their activities? Please can you also tell me what the challenges are that they face in carrying out their responsibilities? How do they try to overcome those challenges and what more could be done to help to overcome those challenges?

*(c) In communities where the CSGs are not functional:*

I understand that there are sometimes challenges in being able to complete the activities that the community support groups have been asked to complete. Please can you tell me what the challenges are that they face in carrying out their responsibilities? How do they try to overcome those challenges and what more could be done to help to overcome those challenges?

1. *(a) In communities where the CSG is functional or partly-functional:*

I think that it might be possible for the CSG to be a link between the facilitators of the regular meetings and the community clinics. What do you think about this idea? How do you think this link could work?

Probe:

- - *Do you think that the CSG members could supervise the work of the volunteers that are facilitating the regular meetings?*
  - *If not, then who do you think could do so?*
  - *Do you think that the CSG members could keep simple records of the activities that take place at the regular meetings and feed them back to the community clinic?*
  - *If not, then who do you think could do so?*

*(b) In communities where the CSG is not functional:*

I need to think about who could provide a link between the facilitators of the regular meetings and the community clinics. What are your thoughts on this?

*Probes:*

- - *Who might be able to supervise the work of the volunteers that are facilitating the regular meetings?*
  - *Who might be able to keep simple records of the activities that take place at the regular meetings?*
  - *Do you think that this is something that the members of the CSG could do? What would help to make this possible?*

Close the interview by asking the participant whether he has any questions or any further information that they would like offer. Thank him for his time.
